# A lesion‐aware automated processing framework for clinical stroke magnetic resonance imaging

**DOI:** 10.1002/hbm.26701

**Published:** 2024-06-22

**Authors:** Patrik Bey, Kiret Dhindsa, Amrit Kashyap, Michael Schirner, Jan Feldheim, Marlene Bönstrup, Robert Schulz, Bastian Cheng, Götz Thomalla, Christian Gerloff, Petra Ritter

**Affiliations:** ^1^ Berlin Institute of Health at Charité, Universitätsmedizin Berlin Berlin Germany; ^2^ Department of Neurology with Experimental Neurology, Brain Simulation Section Charité – Universitätsmedizin Berlin, corporate member of Freie Universität Berlin and Humboldt‐Universität zu Berlin Berlin Germany; ^3^ Bernstein Focus State Dependencies of Learning and Bernstein Center for Computational Neuroscience Berlin Germany; ^4^ Einstein Center for Neuroscience Berlin Berlin Germany; ^5^ Einstein Center Digital Future Berlin Germany; ^6^ Klinik und Poliklinik für Neurologie, Kopf und Neurozentrum Universitätsklinikum Hamburg‐Eppendorf Hamburg Germany

**Keywords:** automation, MRI, processing, reproducibility, stroke, validation

## Abstract

Magnetic resonance imaging (MRI) and MRI based computational modelling studies provide insights into severity and recovery of ischemic stroke patients. The presence of brain lesions, however, can heavily distort and impair state‐of‐the‐art processing frameworks due to abnormal intensity values and tissue distortions. In this study, we introduce and validate the novel “Lesion Aware automated Processing Pipeline (LeAPP).” LeAPP automatically processes clinical stroke MRI data while significantly reducing the impact of pathological artefacts on processing outputs such as structural and functional connectomes (SC, FC). This helps to advance the identification of biomarkers for recovery and mechanism‐based intervention planning using MRI as well as computational approaches. We extended existing frameworks, such as the Human Connectome Project (HCP) minimal processing pipeline, introducing correction steps, and implementing and modifying functional and diffusion processing to cope with MRI acquisition protocols more typical for the clinical context. A total of 51 participants (36 patients, 15 age‐matched controls) were processed across four time points for patients (3–5, 30–40, 85–95, 340–380 days after stroke onset) and one time point for controls. We validated performance using artificial lesioned brains (*N* = 81), derived from healthy brains and informed by real stroke lesions. The quality of reconstructing the ground truth was quantified on whole brain level and for lesion affected and unaffected regions‐of‐interest (ROIs) for brain parcellations and for SCs. Volume based agreement was evaluated using metrics such as dice coefficient, volume difference or ROI center‐of‐gravity distance while SC based agreement was defined as the difference in network metrics (e.g., node strength, clustering coefficient, or centrality). The observed deviations in reconstructed ground truth brain parcellations and structural connectomes from lesioned brains were significantly reduced for LeAPP, compared to the performance of existing pipelines such as HCP minimal processing pipeline. For instance, in the case of lesion affected ROIS we achieved a mean dice coefficient (where a value of one represents total agreement as defined by an exact overlap of both ROIs) of 0.81 with LeAPP, compared to 0.75 for HCP (*p* < .0001*). Additionally, the average measured ROI distance (where a value of zero represents no difference) for lesion ROIs was 0.87 for LeAPP in contrast to 1.7 for HCP (*p* < .0001*), indicating an overall superior performance of LeAPP. The pipeline generates standardized output files ready for brain network modelling for instance with The Virtual Brain software. This novel open‐source automated processing framework contributes to reproducible research and provides a robust framework for automated processing of clinical stroke MRI data, supporting the identification of brain network‐based biomarkers of stroke recovery.

## Graphical abstract

In this study, we introduce a novel lesion aware automated processing pipeline (LeAPP) for clinical stroke patient MRI data. The framework is available as a reproducible and easy‐to‐use software container. It was extensively validated showing improved processing performance, compared to current automated frameworks, in mitigating potential lesion bias and compensating reduced data quality often found in clinical MRI data.

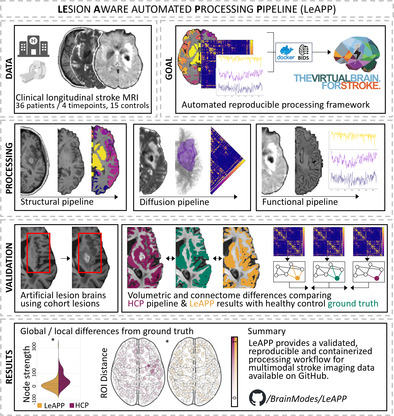

## 1 INTRODUCTION

Processing brain imaging data from stroke patients presents challenges for existing workflows used for standardized processing of multimodal brain imaging data due to a variety of reasons (Chen et al., 2021; Kaffenberger et al., 2022; Liew et al., 2022; Macintosh & Graham, 2013; Moura et al., 2019; Salvalaggio et al., 2020; Siegel et al., 2017; Volle et al., 2013). Particularly in magnetic resonance imaging (MRI), large lesions and their accompanying abnormal intensity values and tissue deformations can cause artefacts and result in performance loss and failure of common processing steps. Such steps can be, for example, surface reconstruction, brain tissue segmentation, or co‐registration of images (Piastra et al., 2022; Siegel et al., 2017; Solodkin et al., 2010). This leads to established neuroimage processing pipelines often resulting in failure or low‐quality outputs when applied to such data sets (Ito et al., 2018; King et al., 2020). Most stroke related MRI studies rely on customized tools and individually crafted solutions (Billot et al., 2022), report insufficient details on lesion specific adjustments made (King et al., 2020) or use standard processing pipelines originally developed for healthy subjects with potentially poor processing outcomes. Thus, a fully automated and reproducible image processing pipeline that correctly accounts for such abnormalities induced by stroke lesions is needed by the scientific and clinical stroke community (Ito et al., 2018). To address this need, we developed and validated a lesion‐aware automated containerized processing pipeline called LeAPP that performs structural, diffusion, and functional MRI processing. We applied this novel pipeline to a longitudinal dataset of stroke patients and healthy controls (Schlemm et al., 2020; Schulz et al., 2016). We used the human connectome project (HCP) minimal processing pipeline (Glasser et al., 2013) as the basis for our workflow incorporating established correction methods to cope with the challenges of stroke brains and adding specific processing steps to address limitations of data acquisition in clinical context.

Those additional correction methods include (1) cost function masking (CFM) (Andersen et al., 2010; Brett et al., 2001) for all co‐registration steps, which removes the lesion signal from the computation of the co‐registration, therefore minimizing lesion impact and improving overall accuracy, and (2) virtual brain transplant (VBT) (Nachev et al., 2008; Solodkin et al., 2010), which aims to approximate the underlying healthy tissue at the focal lesion by using contralesional hemisphere information, enabling downstream processing such as segmentation and surface extraction as well as further analysis of network disruption caused by the lesion itself. Both methods are available within existing frameworks (e.g., in FSL (Jenkinson et al., 2012) and fmriprep (Esteban et al., 2019) for cost function masking or BCBToolkit (Foulon et al., 2018) for performing enantiomorphic normalization) but are not integrated in a fully automated and comprehensive processing pipeline leading to the need for manual and non‐reproducible processing steps.

In addition, we performed an extensive validation of LeAPP and demonstrated significant improvement in reconstructing the underlying subject specific anatomy over the processing results obtained with the HCP pipeline. Furthermore, our pipeline automatically creates data that can serve as input to The Virtual Brain (TVB) (Ritter et al., 2013; Sanz Leon et al., 2013), that can be used to construct patient specific whole brain network models, thus facilitating further research into underlying mechanisms and disease patterns of stroke (Rocha et al., 2022).

## 2 MATERIALS AND METHODS

### 2.1 Ethics statement

The here processed human MRI data were acquired at University Hospital Hamburg Eppendorf (Schlemm et al., 2020; Schulz et al., 2016) and written consent was given by all participants for data acquisition for the original study which was approved by the ethical board of the Hamburg University Hospital (PV3777). The present study was further approved by the Ethics Committee of the Charité Universitätsmedizin Berlin (EA1/222/22).

### 2.2 Patients

A total of 51 participants (36 stroke patients (mean age (standard deviation) = 65.7 (12.96) years, 18 female) and 15 age‐matched healthy controls (mean age (standard deviation) = 69.2 (7.4) years, 7 female)) with complete datasets (see definition in Figure 3) at timepoint 1 (acute phase 3–5 days post stroke onset) were selected from a larger sample (*N* = 80 (43 male)). Where available, longitudinal data acquired over up to three follow up dates (30–40 days, 85–95 days, and 340–380 days post stroke onset) were processed as well (full description of the included data set in Table S5). The data set included structural MRI, task‐based functional MRI as well as diffusion weighted MRI data (Schlemm et al., 2020; Schulz et al., 2016). Initial inclusion criteria for stroke patients were first ever ischemic stroke, hand‐motor deficit without accompanying other functional deficits, and no MRI contra indicators. The full data cohort of healthy controls and stroke patients was used during the development process of LeAPP and processed with the final LeAPP framework, creating derivatives as described below (see Figure 3) and enabling brain network modelling with TVB. The full data set was further used during the validation of the introduced framework via creation of artificial stroke patients (See Section 2.4).

### 2.3 Pipeline

**FIGURE 1:**
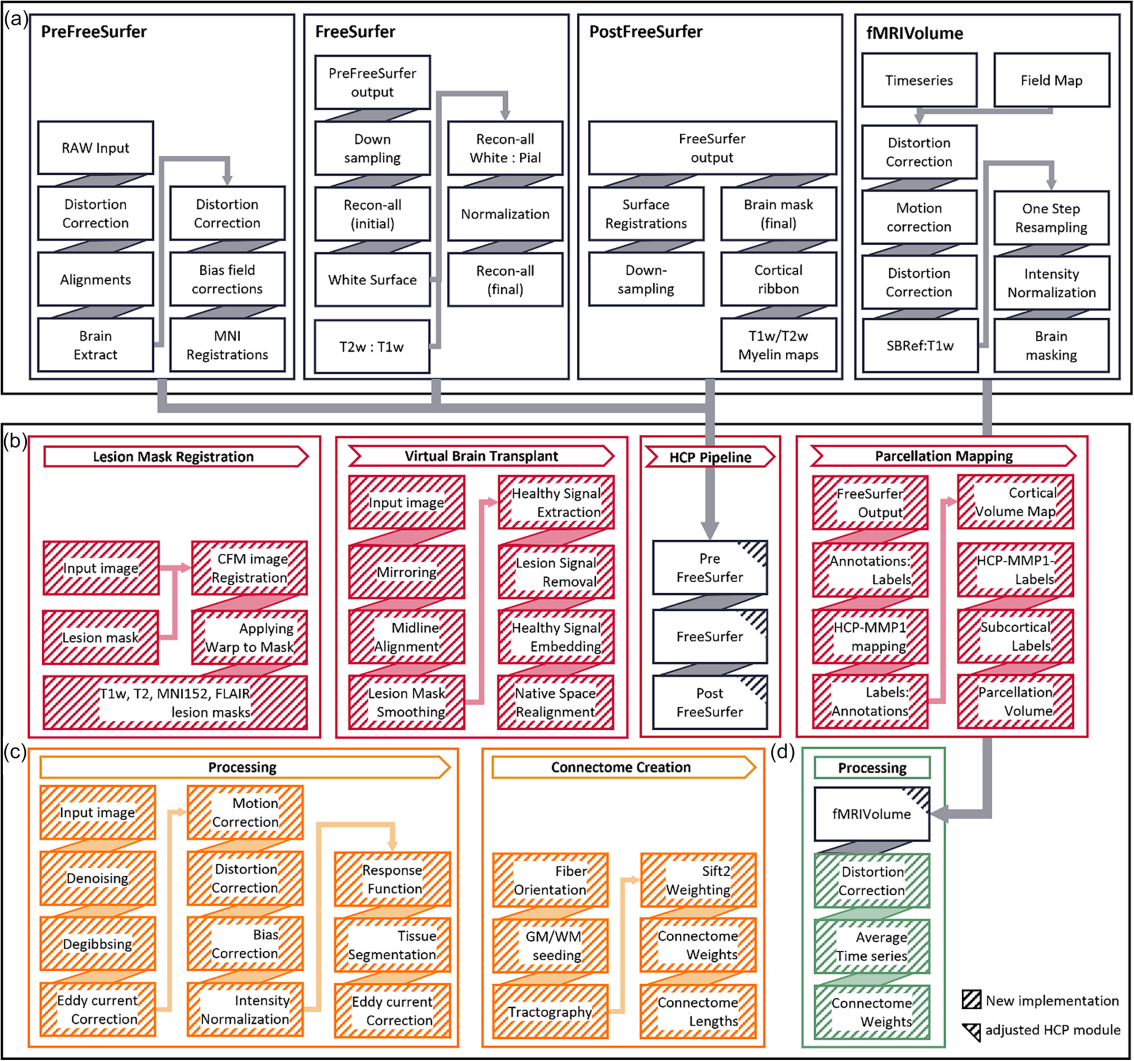
Lesion aware automated processing pipeline (LeAPP) The major processing steps and adjustments for processing of structural (red), diffusion (orange), and functional MRI (green). (a) The Human Connectome Project (HCP) minimal processing pipeline steps as used within LeAPP. Displayed are the integrated high‐level functions, for a more detailed description see (Glasser et al., 2013). (b) Structural processing as implemented in LeAPP includes three additional modules (Lesion Mask Registration, Virtual Brain Transplant, and Parcellation Mapping) which are built around the baseline HCP structural pipeline. The output required for subsequent processing are the fully processed anatomical images in native T1w and MNI space, the created brain parcellation volume, and lesion properties table containing local lesion load for all affected ROIs. (c) The diffusion processing workflow performs the main preprocessing steps, tractography, and connectome creation using the output from structural processing. (d) Functional preprocessing is performed based on HCP functional volume‐based processing module fMRIVolume followed by registration to T1w space for distortion correction and use of the structural processing results including brain parcellation, average time series extraction, and finally computation of functional connectivity.

**FIGURE 2:**
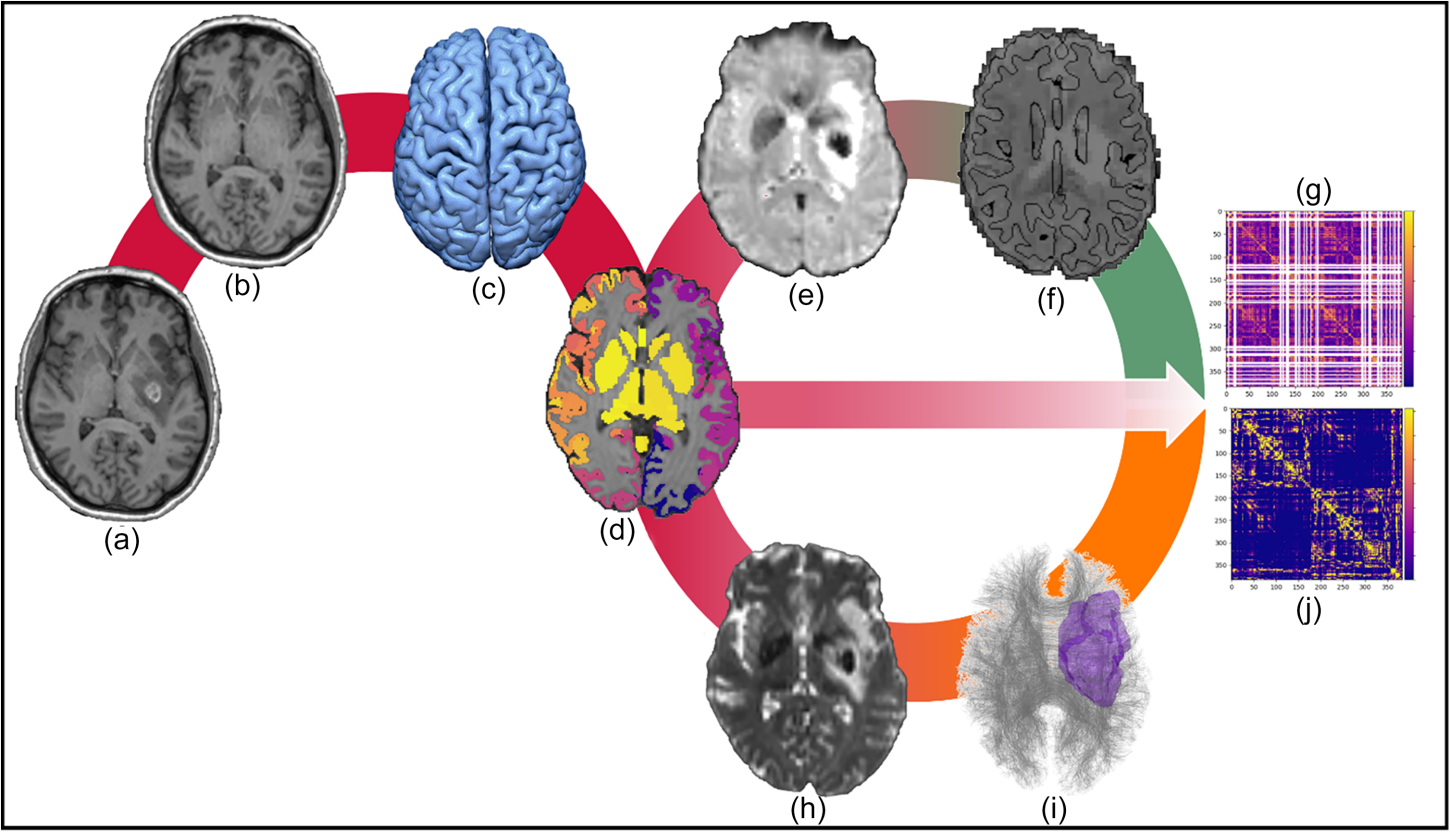
Dataflow showing exemplary processing results for a given stroke patient. The raw lesioned input T1w volume (a) is first processed during LeAPP's initial lesion mitigation steps resulting in a lesion free approximation of the underlying healthy tissue (b). Subsequently the FreeSurfer based processing steps perform surface reconstruction (c) creating a cortical ribbon that is being used to construct individual brain parcellation (d). The functional MRI input data (e) is preprocessed including registration to the processed T1w image (f). In combination with (d) the processed time series is then used to create the functional connectome (g). Similarly, the raw DWI input data (h) is preprocessed. Using the appropriate lesion mask during anatomically constrained tractography individual tractograms are created (i). Utilizing the individual parcellation (d) again the structural connectome is created (j).

**FIGURE 3:**
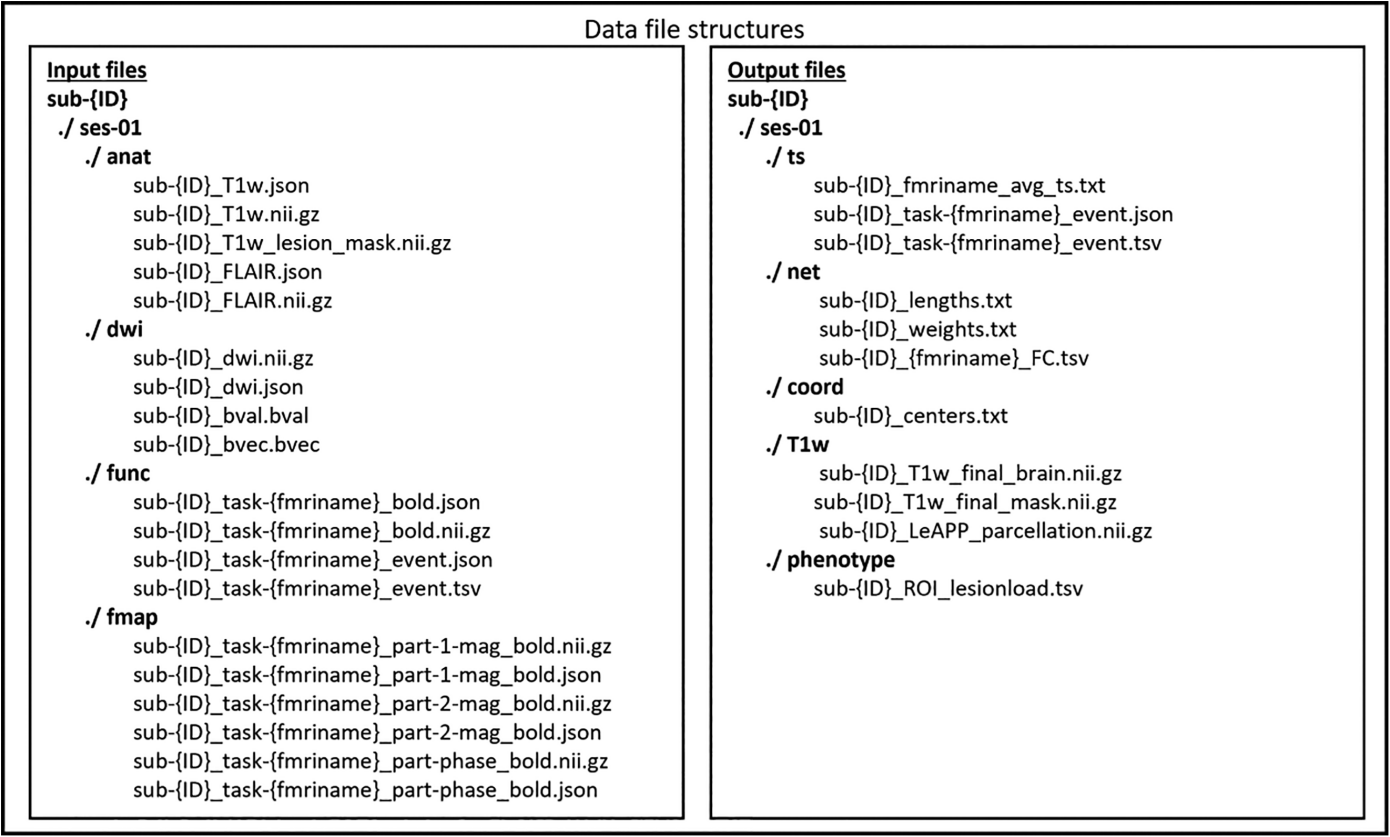
Data file structure. Data input is following BIDS standard for all input modalities (left) including T1w and FLAIR images, DWI images with corresponding bval and bvec files, functional MRI with task event files and corresponding fieldmaps. The main processing results created by LeAPP (right) are stored in a new directory including structural and functional connectomes, parcellation volumes, and corresponding ROI center coordinate file, final T1w images, and lesion mask as well as average ROI based time series with task event files following the BIDS computational modelling extension proposal and can be integrated into the virtual brain (TVB) simulation platform for individual whole brain network modelling.

Image processing was implemented in four steps along the available imaging modalities, structural image processing, diffusion weighted image (DWI) processing, and functional MRI (fMRI) image processing as well as output preparation. Lesion specific mitigation measures were integrated using a priori defined lesion masks. Such masks have been manually drawn using ITK Snap (Yushkevich et al., 2006) incorporating information from both T1w and FLAIR images at UKE Hamburg. Each processing step, as illustrated in Figure 1, was specifically adapted for the challenges of processing clinical stroke lesion MRI data. The data flow from raw input images to the constructed FC and SC matrices is highlighted in Figure 2. The minimally required data to run the structural processing module is a high‐resolution anatomical T1w image, a T2 based image, for example, FLAIR and a corresponding a‐priori defined lesion mask in the case of patient data. To run the modules for the fMRI and DWI modalities requires the structural processing results and, therefore, requires the mentioned anatomical images as well as modality specific volumes. The specific imaging volumes required for each module are shown in Figure 3.

#### 2.3.1 HCP minimal processing pipeline

The well‐established HCP minimal processing pipeline (Glasser et al., 2013) forms the basis of LeAPP. It's structural processing pipeline follows a three‐step composition (*PreFreeSurfer*, *FreeSurfer*, and *PostFreeSurfer*), built around the surface reconstruction framework FreeSurfer (Fischl, 2012) (Figure 1a). *PreFreeSurfer* performs initial correction steps, including gradient distortion and bias field corrections. It further aligns the images to standardized orientations and across modalities and provides an initial extracted brain for subsequent processing. The following *FreeSurfer* module uses the preprocessed images created by *PreFreeSurfer* and performs surface reconstructions. It first creates the white matter surface followed by the pial surface while repeatedly performing additional interim normalization and segmentation steps compared to the baseline FreeSurfer functionality. In a last step, it creates surface and volume parcellations including the estimation of measures of cortical thickness. The third structural module *PostFreeSurfer* subsequently creates standardized Nifti and Gifti images of the previous processing results including surface meshes and parcellation labels. It further creates the final alignments of the T1w and corresponding T2 images and performs a down sampling of the standardized surfaces. It finally produces an individual cortical parcellation volume based on the extracted white and pial surface distances.

In addition to HCP's structural processing pipeline, LeAPP also uses the *fMRIVolume* pipeline. This processing step first performs gradient distortion correction followed by motion correction via realignment to a single‐band reference image. Using available fieldmap images it performs distortion correction along the phase encoding direction and finally T1w and MNI registration including brain masking and normalization, resulting in a 4D timeseries volume.

#### 2.3.2 Structural imaging pipeline

The structural imaging processing pipeline (Figure 1b) extends the HCP minimal processing pipeline's structural processing (Glasser et al., 2013) to be more robust and broadly applicable when processing MRI data in the presence of stroke lesions. First, it incorporates CFM in all registration steps, which is necessary to ensure accurate registration of lesioned images (Andersen et al., 2010; Brett et al., 2001) by restricting the fitting of the co‐registration to voxels outside of the provided lesion mask, therefore reducing the influence of lesion‐based abnormalities on the alignment process. Three additional steps are included that add to the HCP structural processing pipeline: (1) the automated transformation of the a priori provided lesion mask, (2) the first fully automated implementation of VBT and (3) the creation of subject specific brain parcellations. To enable CFM across all processing steps within the pipeline for all structural input modalities including T1w, T2w, and/or FLAIR, as well as the necessary MNI template spaces and resolutions, the initially provided lesion mask is co‐registered to fit the given modality. Such an alignment is performed by first fitting the linear affine co‐registration using the corresponding base image (e.g., T1w image for a lesion mask created in T1w space) and registering it to either the corresponding modality or MNI template image and the resulting transformation is then applied to the binary lesion mask followed by inversion to capture all voxels not included in the lesion. This process ensures accurately spatially aligned lesion masks for all structural imaging modalities by enabling cost function masking (CFM) by masking the lesion voxels in both the input image as well as the reference image that the input image is co‐registered to. The final list of aligned lesion masks consists of the native T1w space, T2, or FLAIR space depending on data available, MNI152 for both 1 and 2 mm voxel resolution. During the application of CFM across all co‐registration steps, the lesion voxels that are encoded as zeros in the aligned mask, were, therefore, excluded from the registration fitting process. To enable downstream processing, following (Nachev et al., 2008; Salvalaggio et al., 2020; Solodkin et al., 2010), we integrated VBT via embedding of contralesional hemisphere signal into the lesion area, creating an approximation of the underlying healthy brain tissue at the lesion.

To this end, the co‐registration of the raw input to its mirror image is computed and the resulting transformation matrix is halved, resulting in a transformation that moves each point in alignment with the midline. The initial lesion mask for the corresponding modality is also aligned at the midline and inverted. Before extraction of both lesion and healthy signal, the border of the corresponding lesion mask image is smoothed by applying a Gaussian kernel (with 2 mm full width at half maximum) to ensure a more seamless integration into neighboring voxels during imputation. To extract the healthy brain signal from the mirror image and remove the lesion signal of the input image, the aligned lesion mask is multiplied with the midline‐aligned mirror image. The inverse of the lesion mask, containing values of one for all voxels but the lesion, is then applied to the midline‐aligned input image, removing the lesion signal. The healthy signal is then added to the lesion free input image to create an approximation of the underlying healthy brain tissue. To complete the implementation of VBT, the inverse midline transformation is applied to realign the transplanted image back into native patient space of the original input image. This is applied to both T1w and corresponding T2 or FLAIR images (for a detailed description, see Supporting Information Methods Virtual Brain transplant and Figure S1). Following VBT, the CFM adjusted structural processing steps PreFreeSurfer, FreeSurfer, and PostFreeSurfer of the HCP pipeline are initiated. Subject‐specific brain parcellations are created in the final step of the *structural image pipeline*. A total of 383 distinct brain regions‐of‐interest (ROI) are identified using a combination of the HCP‐MMP1 atlas (Glasser et al., 2016) for cortical and FreeSurfer's subcortical areas (Fischl, 2012). To ensure sensitivity for individual brain topologies this study follows (CJNeurolab, 2018) performing the mapping of HCP‐MMP1 regions on the cortical labels created during surface extraction using a previously published mapping of HCP‐MMP1 annotation labels in fsaverage space (Kathryn Mills, 2016) (see Table S6 for full list of ROIs). The resulting annotation files are then mapped back into volume space to create an accurate parcellation of the subjects cortical and subcortical regions at lesion free areas as well as a substantiated approximation of the underlying regions at the lesion location within high resolution individual subject T1w space. In a final step, the parcellation image is multiplied with the final lesion mask in T1w space to extract the lesion load per ROI, defined as the number of affected voxels divided by the total number of voxels for a given ROI.

#### 2.3.3 Diffusion imaging pipeline

Due to limitations in data acquisition in the clinical context, such as time constraints, potentially limiting the extent of available data, the existing diffusion weighted imaging (DWI) framework within the HCP minimal processing pipeline did not present a feasible approach for the clinical patient population of this study. This is most prominent in the lack of reverse phase encoding images in the present cohort data, which represents a clear requirement for the distortion correction as performed in the HCP diffusion pipeline. The HCP processing framework additionally expects high quality DWI data with regards to resolution (maximum 1.5 mm voxel size) as well as a large number of acquired unique directions (at least 128). Neither of these requirements are met within most clinical MRI data sets, including the cohort used in this study. Hence, a new DWI processing pipeline was implemented using the MRTrix3 (Tournier et al., 2019) software package. The main processing steps for the DWI pipeline (Figure 1c) are (1) preprocessing and normalization of raw input images, (2) tissue segmentation of the corresponding anatomical image and finally (3) tractography and connectome creation (The full list of steps is listed in Table S2). Preprocessing steps are comprised of denoising, degibbsing, eddy current and motion correction, co‐registration to T1w space for distortion correction due to the absence of reverse phase encoding data and lastly bias correction. The images are subsequently intensity normalized at the group level. The preprocessed T1w image, created during the structural processing module including lesion mitigation measures, is segmented into five tissue types using the *MRtrix3 5ttgen* function and the corresponding lesion mask is integrated as pathological tissue type. This enables anatomically constrained tractography (ACT) (Smith et al., 2012), after group level response function estimation, while integrating information about lesion pathology in the estimation of tract probabilities for voxels within the lesion mask. The default number of streamlines created is 100 million feasible tracks with the previously segmented grey matter—white matter barrier as the seed region. The individual brain parcellation created during structural processing is used to define ROI boundaries to construct the structural connectome using the SIFT2 algorithm (Smith et al., 2015).

#### 2.3.4 Functional imaging pipeline

Processing of functional MRI data (Figure 1d) follows the HCP's fMRIVolume pipeline processing step (Glasser et al., 2013) (Figure 1b) updated with CFM. It further integrates the additional steps of (1) co‐registration to T1w space and (2) the computation of ROI based average time series and the functional connectome. The preprocessed output volume of the adjusted fMRIVolume pipeline is linearly co‐registered to the preprocessed subject T1w image output from the *structural pipeline* using the created single band reference image and CFM. The resulting transformation is applied to the 4D time series data extracted during the fMRIVolume step to create fMRI data in subject native T1w space. Following this registration, the previously created brain parcellation volume is used to extract voxel time series for each ROI individually followed by spatial averaging within each ROI using *fslmeants* function (Jenkinson et al., 2012). Subsequently functional connectomes (FC) for the full time series data are created using Pearson correlation coefficient of the average time series across all ROIs.

#### 2.3.5 Output preparation pipeline

The final step of the LeAPP pipeline creates a comprehensive standardized collection of the final processing results within a single directory called TVBReady (Figure 3). This set of files includes the processed T1w image and lesion mask, the corresponding brain parcellation volume, functional connectomes and average time series for all processed tasks, structural connectome weights, and tract lengths as well as a list of ROI specific lesion loads and ROI center coordinates file. The format of the output files follows the current Brain Imaging Data Structure (BIDS) (Gorgolewski et al., 2016) computational modelling extension proposal (Schirner & Ritter, 2021), enabling further reproducible research into ischemic stroke patients based on MRI and a direct creation of brain network models via the integration of processing results into software packages such as TVB.

### 2.4 Validation

Following previous studies (Andersen et al., 2010; Crinion et al., 2007; King et al., 2020; Radwan et al., 2021), we evaluated the performance of LeAPP by creating artificial stroke patient brains. This approach enables the direct comparison of processing results of lesioned data with a given ground truth, represented by the healthy control brain without any lesion pathology. This comparison allows for a straightforward evaluation of the frameworks capability of reconstructing the underlying brain topology without artefacts and distortions introduced by the lesion signal itself.

#### 2.4.1 Artificial lesion embedding

**FIGURE 4:**
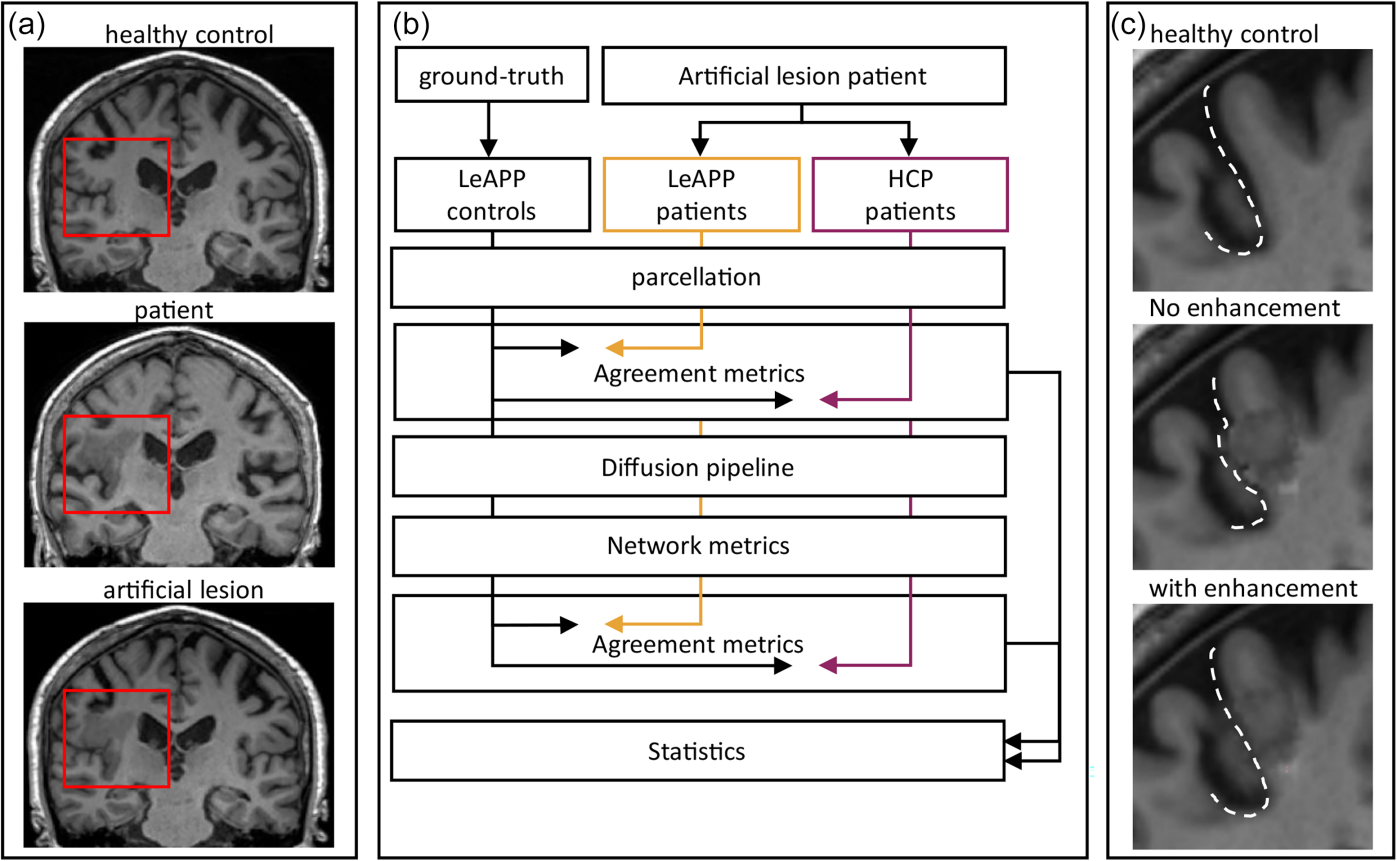
Validation framework. (a) Example of the implemented artificial lesion embedding (ALE) approach showing the original healthy control T1w image (top), the original patient data used for extracting lesion signal (middle) and the constructed artificial lesion patient in the same T1w (bottom). (b) The resulting ALE data set presents a realistic approximation of a virtual stroke patient as basis for a robust validation process. To validate processing performance, the created validation data set is processed creating three distinct cohorts (ground truth processed with HCP, ALE patients processed with LeAPP, and ALE patients processed with only baseline HCP structural processing). Based on the resulting parcellations global and local agreement measures are computed between ground truth and the ALE based results. The distributions of agreement measures are compared between LeAPP and baseline HCP based results. The brain parcellations are used for structural connectome creation using ground truth DWI data. A range of global and local network metrics are computed for the resulting structural connectomes and agreement between metrics of ground truth and the ALE based connectomes are computed. The distributions of these agreement measures are compared between LeAPP and baseline HCP (c) Details showing the enhancements effect during ALE of smoothing and thresholding the lesion mask to fit the underlying healthy control brain topologies more closely creating realistic artificial lesion brains.

To create the required validation data set brains, artificial lesion embedding (ALE) was performed by imputation of stroke patient lesion signal into a healthy control brain volumes (Figure 4a). The method is closely related to the above mentioned VBT method but differs in the following aspects: instead of imputation of healthy contralesional hemisphere signal within the same patient brain, lesion signal from a different subject (stroke patient) was used to replace healthy tissue of the subject (healthy control) which in turn represented a different co‐registration approach between subjects, as opposed to mirror images of the same subject as in VBT. To achieve seamless embedding of lesion signal into the healthy brain, the healthy control input image was first linearly co‐registered to the patient input image with CFM applied. To ensure meaningful artificial lesions, the individual anatomical topology of the healthy brain needs to be considered during the embedding. To this end, the patient lesion mask was adjusted by removing voxels whose intensity values in the co‐registered healthy image were below the threshold of the five percentile (Ito et al., 2018) representing intensity values usually found outside the healthy brain in anatomical modalities, that would not be affected from naturally occurring lesion manifestations. This helps to avoid the imputation of lesion signal to voxels that represent, for example, ventricles, fissures, or skull in the resulting artificial stroke patient brains. Maintaining the original topology of the healthy brain was necessary to allow for a meaningful validation of the processing framework as opposed to unrealistic artificial stroke lesions potentially introducing biases not representative of true patient populations as highlighted in (Figure 4c). The updated lesion mask—adjusted for individual topology of the healthy brain—was smoothed as in VBT and used to extract the lesion signal from the patient image and remove brain signal from the co‐registered healthy subject image. The extracted lesion signal was normalized by division through the maximum intensity value of the lesion and followed by multiplication with the maximum intensity value of the healthy tissue. This simplistic normalization ensures a similar intensity value range with the surrounding healthy tissue, while keeping the abnormal variance pattern of the lesion signal intact. Finally, the inverse transformation of the initial co‐registration to patient space was applied to create realistic artificial patient image for an existing healthy control subject. This was performed for both T1w and FLAIR anatomical images. Following the creation of artificial lesion brains, the validation data, therefore, consisted of three separate datasets (Figure 4b): the original healthy control brain data used to generate the artificial stroke patient brain data, which serves as ground truth (LeAPP controls); the artificial stroke patient brain data processed with LeAPP (LeAPP patients); and the artificial stroke patient brain data processed using the HCP structural processing pipeline (HCP patients).

#### 2.4.2 Reconstruction quality metrics

To evaluate the performance of LeAPP, we compared the processing results of LeAPP and the HCP processing pipeline and their ability to reconstruct the ground truth brain parcellations and connectomes, which were created from the healthy brains before their anatomical images had been artificially distorted through the artificial insertion of a stroke lesion. The goal, and hence quality criteria for pipeline performance, was the recovery of original parcellations and connectomes of the healthy brains—despite the challenges introduced by stroke lesions (Ito et al., 2018; Koch et al., 2019; Liew et al., 2022; Siegel et al., 2017) (Figure 4b). We chose the following metrics to first assess the agreement of the individual brain parcellations, providing information on three base errors of segmentation commonly evaluated in medical image segmentation: area, content and contour (Shi et al., 2013). To this end dice coefficients, Jaccard scores, volume difference (Bertels et al., 2019; Taha & Hanbury, 2015), and Euclidean distance of the ROI center‐of‐gravity in voxel space were computed to capture complimentary properties regarding the accuracy of reconstruction. Dice coefficient is a frequently used metric to validate medical image segmentations (Abbasi et al., 2023; An et al., 2023; Dhar et al., 2020; Kaffenberger et al., 2022; Radwan et al., 2021; Wong et al., 2022) and provides an estimate of the spatial overlap of two volumes. Jaccard score is closely correlated, as another overlap‐based metric of agreement (Taha & Hanbury, 2015). Together they approximate the base errors of area and contour capturing the main properties of agreement between, for example, two ROIs. A value of one represents total agreement as defined by an exact overlap of both ROIs. Volume differences (An et al., 2023; Andersen et al., 2010; Kuang et al., 2020; Taha & Hanbury, 2015) have been computed to assess the similarity in content, providing an estimate of potential distortions regarding the overall size of the volumes. To further capture, complimentary information about the overall spatial distance of the compared objects (Taha & Hanbury, 2015), the Euclidean distance between the center‐of‐gravities was computed.

**FIGURE 5:**
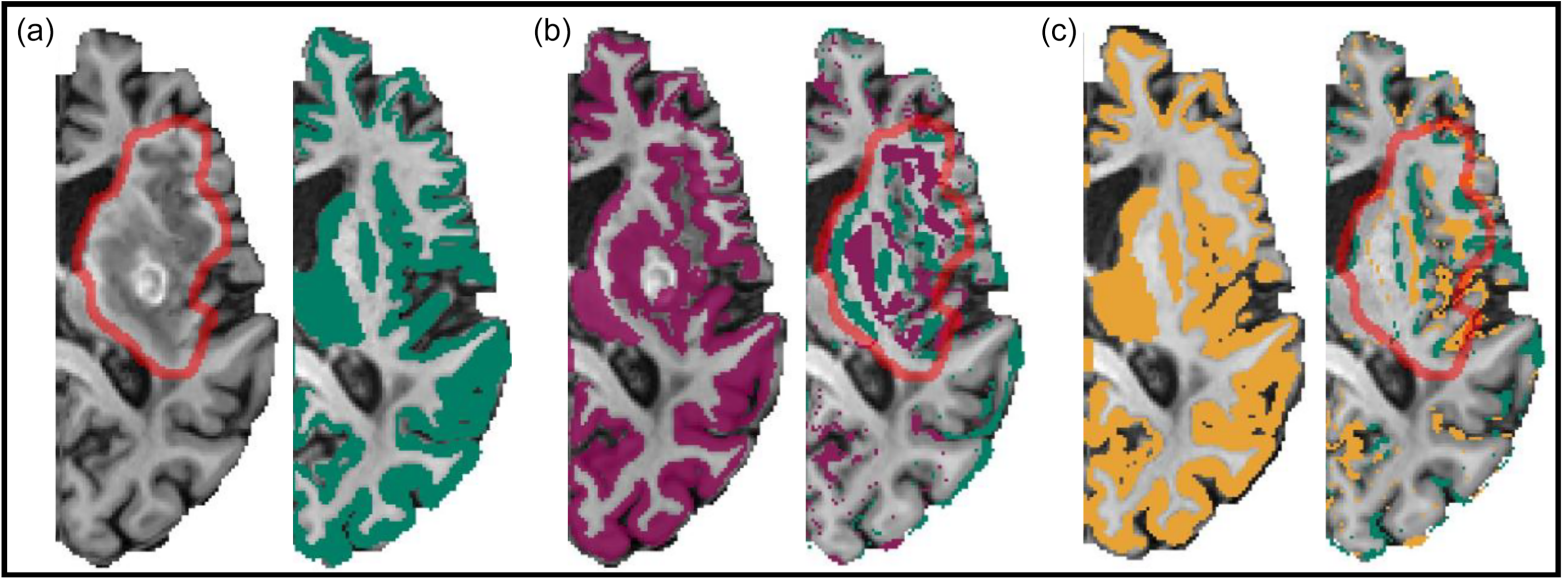
Parcellation masks. Example artificial lesion brain and corresponding Processing results. (a) ALE brain with imputed adjusted lesion signal (red outline) and ground truth parcellation created for healthy control used during ALE (green). (b) Parcellation mask created using HCP processing pipeline and parcellation mapping (purple) showing a clear reproduction of lesion topology during segmentation and surface reconstruction. The difference between ground truth (green) and HCP (purple) with color coding voxels that are only present in the corresponding parcellation (right). (c) Parcellation mask created using LeAPP (yellow) and corresponding difference mask (right). While a mismatch between parcellations for LeAPP and the ground truth pertains, it shows a reduced difference at the lesion (red outline) compared to HCP.

To first validate global volumetric differences between processing frameworks, the level of agreement was evaluated by comparing the binarized full brain parcellations, created during the structural processing step, containing cortical and subcortical brain areas within a single volume mask (Figure 5). Additionally, local differences were investigated by extracting the corresponding volumes from ground truth and both ALE based individual brain parcellations for each ROI individually and computing all agreement metrics.

In a second step, processing impact on connectomes was investigated focusing on the overall connectivity and integration of the created networks. Hence node strength, centrality, and clustering coefficient (Bullmore & Sporns, 2009; Falcon et al., 2015; Siegel et al., 2018; Yang et al., 2015) were computed for each structural connectome and differences in these metrics between processing pipelines were defined as the connectome‐based level of agreement. Node strength provides an estimate of the overall connection of a given node, here the ROI of the brain parcellation, with all other nodes of the network, incorporating not only the existence of a connection between two nodes but the strength of the connection as well. Betweenness centrality is commonly used to assess the relative role of a node within a network in respect to efficient information exchange due to the number of shortest paths passing through the given node. Clustering coefficient provides an additional measure on the presence of well‐connected local groups of nodes within the overall connectome (Bullmore & Sporns, 2009). Similar to the previous parcellation based validation, global impact was first investigated, using the averaged node strength and betweenness centrality over all ROIs as well as the clustering coefficient of the full connectomes. Local differences were subsequently evaluated by comparing the node strength and betweenness centrality for each ROI individually. Statistical analysis of reconstruction accuracy was performed on the differences in these network metrics between the ground truth and the given ALE‐based processing results, comparable to the volume‐based agreement metrics.

#### 2.4.3 Statistical analysis

Python software package (Van Rossum & Drake, 2009) was used for statistical analysis as integrated in the containerized validation framework of this study. One‐sided dependent t‐tests (alpha = .05) were computed to compare LeAPP with the HCP on both volume‐based and network‐based reconstruction quality metrics (Figure 4b) reporting both the p‐value for significance and t‐statistic as a measure of strength in difference between the compared distributions. Instances of a single lesion affected ROI in one subject were excluded, as distribution‐based comparisons were not possible.

## 3 RESULTS

### 3.1 Artificial lesion embedding

To create a sufficiently large artificial stroke patient data set with a known ground truth for validation, the described ALE framework was performed based on all healthy control participant data available in the present cohort. To avoid any selection bias in the combinations of healthy controls and stroke patients, a random subset of 15 brain volumes out of all available 36 stroke patients was created for each healthy control individually. This iterative approach resulted in a total of 225 artificial stroke brains. Visual quality control was performed to identify ALE data sets with unrealistic and non‐feasible lesion embeddings, such as lesions ranging into cerebrospinal fluid or skull, or similar features, representing lesion not manifested in real stroke patient populations, creating a clear distinction as artificial (see Figure S2). Such quality controls were necessary, as the random combination of subjects can lead to anatomical mismatches between source and target brain during lesion imputation, for example, in brain size, that can cause inconsistencies in the ALE brain not resolved with the previously described thresholding and smoothing. A total of 81 ALE samples remained for validation.

### 3.2 Processing performance

The introduced framework LEAPP showed its general applicability for processing of low quality structural and functional stroke patient MRI data as no stroke patients had to be excluded due to lesion topology causing the processing pipeline to fail as previously reported on the same data set (Schlemm et al., 2020), showing its direct relevance for clinical data. Validation comparing LeAPP with existing processing frameworks further showed superior performance in reconstructing ground truth brain topologies.

#### 3.2.1 Human connectome project minimal processing pipeline

**FIGURE 6:**
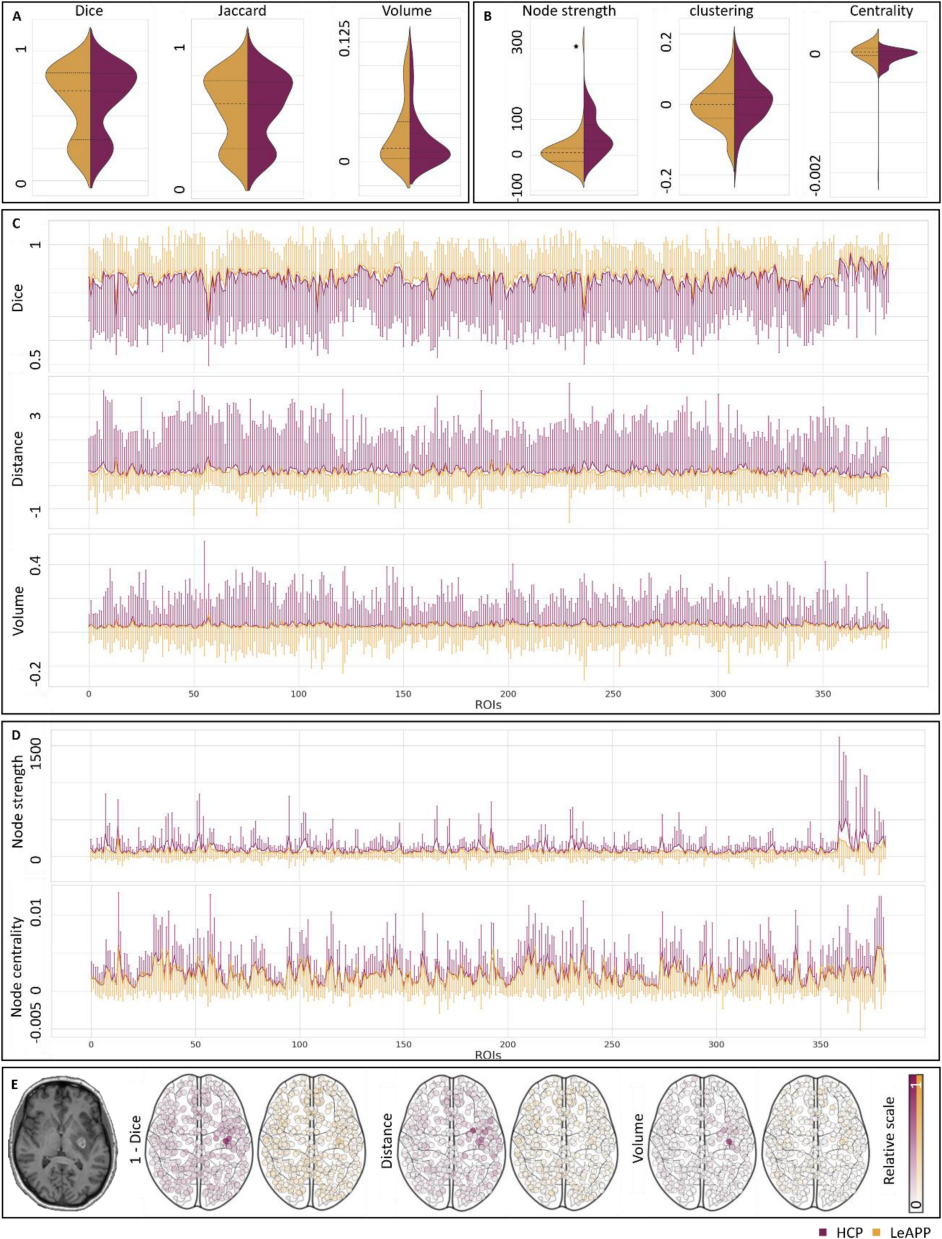
Reconstruction quality results (a) Metrics for LeAPP (yellow) and HCP (purple) showing similar distributions across global measures. (b) Differences between LeAPP and HCP in global network metrics show a significant reduction in average global node strength difference between LeAPP and ground truth compared to HCP and ground truth differences. (c) Median local ROI based agreement metrics (line) with standard deviation shown as error bars. For visualization, only one‐sided error bars are displayed. (d) Median differences in network based local metrics for all ROIs also showing significantly smaller differences for LeAPP over HCP (see Table 1). Local differences highlight the increased sensitivity of ROI based measures in contrast to global measures based on full brain properties. (e) Exemplary ROI based agreement metrics for a single subject. Anatomical T1w image (left) shows the clear lesion artefact. The normalized agreement metrics reproduce the largest differences in processing around the focal lesion visible in the MRI image. Dice score is shown as 1 − dice score for consistency in visualizations.

The LeAPP pipeline with its integrated correction methods showed improved processing performance, compared to the baseline HCP pipeline for brains with stroke lesion. While global parcellation‐based volumetric agreement measures show no difference between processing pipelines (dice score *p* = .76, Jaccard score *p* = .49, volume difference *p* = .56; Figure 6a), region‐wise comparisons found significant improvements of LeAPP across all volumetric agreement measures for directly lesion affected and not‐directly affected ROIs (dice score *p* < .001 and *p* = .0, volume difference *p* < .001 and *p* < .001; Figure 6c). We found a similar impact on the downstream processing step of creating structural connectomes. A significant difference in global average node strength was identified while the difference in full brain clustering coefficient and average centrality did not show significance (node strength *p* < .001, clustering coefficient *p* = .08, node centrality *p* = .19; Figure 6b). Like for the volumetric reconstruction quality measures for parcellations, we found a significant local impact of processing framework on network level as well, with differences in node strength for directly lesion affected and not‐directly affected ROIs and centrality for not‐directly affected ROIs (node strength *p* < .001 and *p* = .0, node centrality *p* = .25 and *p* = .02). Table 1 presents all test statistics comparing reconstruction quality metrics for LeAPP and HCP structural processing of the validation data set including effect sizes. While the lower number of directly lesion affected ROIs might contribute to the reduced effect sizes and lack of significance in, for example, ROI based node centrality compared to not‐directly affected ROIs, an additional interesting finding of this study is that all local measures showed significance for the subset of not‐directly lesion affected ROIs, highlighting lesion impact in downstream processing beyond the focal lesion. The feasibility of the results is further strengthened when investigating differences in reconstruction metrics on individual subject level, as shown in Figure 6e, displaying the largest differences between processing frameworks around the location of the lesion for an exemplary ALE subject.

**TABLE 1:** Test statistics for reconstruction quality.

Measure	Mean LeAPP (var)	Mean HCP (var)	*p*‐value (statistic)
Global			
Dice	0.69 (0.044)	0.69 (0.042)	0.52 (−0.05)
Jaccard	0.57 (0.055)	0.57 (0.052)	0.5 (0.002)
Volume	−0.011 (0.002)	−0.012 (0.001)	0.47 (0.08)
Node strength	9.47 (2205)	52.94 (2849)	<0.001 (5.67)*
Clustering coefficient	−0.0024 (0.0035)	0.0107 (0.0041)	0.08 (−1.8)
Node centrality	−3.01e−05 (6.7e−08)	−7.3e−5 (1.2e−08)	0.19 (1.31)
Local			
Dice lesion affected	0.81 (0.018)	0.75 (0.049)	<0.001 (9.34)*
Dice not affected	0.82 (0.017)	0.76 (0.046)	0.00 (39.09)*
Distance lesion affected	0.87 (0.77)	1.7 (7.42)	<0.001 (−10.53)*
Distance not affected	0.8 (0.69)	1.32 (3.74)	0.00 (−52.3)*
Volume lesion affected	−0.018 (0.026)	−0.02 (0.058)	<0.001 (−6.76)*
Volume not affected	−0.008 (0.022)	−0.019 (0.043)	<0.001 (−21.1)*
Node strength lesion affected	220.7 (71215.14)	635.3 (477593.4)	<0.001 (−19.37)*
Node strength not affected	87.1 (13587.4)	126.3 (24750)	<0.001 (−31.7)*
Node centrality lesion affected	0.0035 (1.6e−05)	0.0036 (1.5e−05)	0.32 (−0.46)
Node centrality not affected	0.0029 (1.1e−05)	0.0030 (1.1e−05)	0.037 (−1.92)*

*Note*: Differences between LeAPP and HCP processing were evaluated via reconstruction quality measures for created brain parcellations and differences in network metrics for structural connectomes. Global measures (top) did not show significant differences for brain parcellations but a significantly smaller difference in average global node strength between ground truth and LeAPP versus ground truth and HCP based connectomes. Local (bottom) differences were significant for brain parcellations across measures. Differences in local network measures were significant for node strength for affected and not affected ROIS. Centrality did not show differences in lesion ROIs but overall, in not‐affected ROIs.

* Significance.

#### 3.2.2 fmriprep

To further highlight the performance of LeAPP we also validated the impact of lesion pathology on processing performance in comparison to the established fmriprep processing framework (Esteban et al., 2019) showing again a higher performance of LeAPP to reduce lesion induced distortions in processing (see Figures S3–S6 and Table S1). Here the local impact of lesion signal, as determined via comparison of mean agreement values between lesion affected and unaffected ROIs, was significant for fmriprep for dice (*p*‐value <.0001*), volume difference (*p*‐value = .047*) and ROI distance (*p*‐value <.0001*). In the case of LeAPP such differences were not significant for neither dice (*p*‐value = .992), volume difference (*p*‐value 0.22) nor ROI distance (*p*‐value = .55), showing a significantly reduced impact of lesion pathology in the processing with LeAPP in comparison to fmriprep as well.

### 3.3 Processing pipeline

The main result of this study is the open‐source containerized processing framework LeAPP. It is freely available from the corresponding GitHub repository (www.https://www.github.com/brainmodes/leapp) and docker hub. LeAPP's modular approach allows for easy to use and automated processing by providing a simplified call structure. The code snippet below shows an example call using the docker containerization software running the structural processing pipeline for stroke patient data including lesion mitigation. The call structure is built around the processing modules described in Section 2. The necessary data to perform each processing step is described in Figure 3 for the raw input data. The full list of available submodules and corresponding dependencies of interim processing results is given in Table S4.

**Table:** 

Code	Comments
*docker run* *‐v “Path/To/StudyFolder”: /data* *‐e Steps=“structural all lesion”* *‐e SubID=“P001”* *leapp:processing*	Base docker command to run a container Mounting StudyFolder directory into container Processing steps to perform in a single string Subject ID following BIDS naming convention Name of container to be run by docker

*Code snippet* demonstrating a simple structural processing call for a given patient. Path/To/StudyFolder is expected to contain the data set for all subjects following BIDS naming conventions. Steps variable defines the processing module “structural,” the corresponding processing step within the module “all” as well as the flag for integrating the implemented mitigation measures “lesion.” The subject identifier “SubID” follows BIDS naming convention and is internally combined with the prefix “sub‐.”

## 4 DISCUSSION

### 4.1 Previous work

While MRI is the standard for noninvasively investigating brain tissue damage and has been widely applied in both research and clinical diagnosis (Burgess & Kidwell, 2011; Czap & Sheth, 2021; Li et al., 2023) most existing processing frameworks have not been systematically investigated for the impact of lesion pathology (King et al., 2020). A wide range of processing frameworks have been introduced for the automated processing of MRI data. The current gold standard for high quality multimodal MRI data, the HCP minimal processing pipeline (Glasser et al., 2013) has been used extensively for a large number of use cases. (Schirner et al., 2015) and (Frazier‐Logue et al., 2022) introduced processing pipelines with the specific goal of enabling brain network modelling with TVB also using multimodal MRI data. Even more frameworks have been introduced focusing on the processing of certain modalities such as diffusion MRI (Tournier et al., 2019) or functional MRI (Esteban et al., 2019). Most of the existing frameworks, however, rely on specific software packages such as FreeSurfer (Fischl, 2012) to perform surface reconstruction and related structural processing steps. Such algorithms have been previously shown to suffer from errors during surface reconstruction in the case of lesioned data (King et al., 2020; Solodkin et al., 2010) highlighting the need for artefact free high resolution imaging data for existing automated frameworks. In the presence of pathological data, previous works introduced several approaches to mitigate the lesion impact, in particular cost function masking (Andersen et al., 2010; Brett et al., 2001) and enantiomorphic normalization (Nachev et al., 2008; Solodkin et al., 2010) as integrated within the current LeAPP pipeline. These mitigation approaches have been previously integrated into processing software (e.g., CFM within fmriprep (Esteban et al., 2019) and enantiomorphic normalization within the BCBToolkit (Foulon et al., 2018)) and further developed, utilizing current advances in machine learning, introducing, for example, deep learning based lesion normalization measures (Radwan et al., 2021). None of these advances, however, resulted in a fully automated approach for the processing of multimodal acute clinical stroke data as needed within the stroke neuroimaging community (Ito et al., 2018).

### 4.2 Summary

Therefore, the objective of this study was to design and validate an automated reproducible processing pipeline that can specifically meet the challenges of processing multi‐modal acute stroke patient clinical MRI data, that also enables whole brain network simulations based on stroke MRI data using TVB.

In summary, we show that LeAPP leads to a significant improvement in retaining important anatomical brain information when processing MRI data that are affected by stroke lesions. The validation approach presented here shows that LeAPP is widely applicable from structural analysis to connectomics. An additional benefit of LeAPP is that it automatically creates TVB‐ready data to facilitate accurate brain network modelling (BNM) for stroke. LeAPP further allows for introduction of ROI specific information such as lesion load, that had been previously linked to network disruption (Griffis et al., 2019), into individual downstream analysis such as BNMs. The following part of this article moves on to discuss several aspects of such a multi‐modal processing approach and presents remaining challenges for the field to enable accurate mitigation of lesion induced artefacts, while at the same time minimizing methodological and data‐driven biases on processing and downstream analysis of stroke patient MRI data.

The clinical context of data acquisition for acute stroke patients often severely limits the availability of scanning protocols. This leads to the common practice of manual adjustments and corrections during processing for each subject, reducing the automation potential and reproducibility, or applying pipelines initially used for varying patient populations (King et al., 2020; Schlemm et al., 2020). Our results show the advantage of LeAPP over such prior frameworks, in processing low quality functional imaging data (Figure SV2) as well as low resolution FLAIR imaging, which is often acquired instead of a T2 image in the clinical context of stroke (Leiva‐Salinas & Wintermark, 2010; Li et al., 2023). It further represents, to the best of our knowledge, the first complete and automated multi‐modal processing pipeline incorporating advancements of correction methods, that have previously only been available as stand‐alone solutions (Radwan et al., 2021; Solodkin et al., 2010). These improvements, in addition to providing high quality reconstruction of volume and network information from MRIs, help to avoid excluding data due to poor quality or large artifacts, thereby facilitating new research with larger cohort and longitudinal studies in the future.

### 4.3 Limitations

Previous studies have shown an increase in accuracy of image registration (Andersen et al., 2010; Crinion et al., 2007) when applying CFM to lesion data. Nevertheless, a residual distortion of processing results after applying CFM cannot be avoided (Bey et al., 2022) leading to a baseline error in processing. Further research is needed to better understand the impact of CFM and account for its effects on group level comparisons.

The extent of lesion abnormality in MRI images, such as flattened intensity value distributions (Figure SV1), has previously been shown to depend on various factors, such as imaging modality and relative time of acquisition to onset (Madai et al., 2016; Scheldeman et al., 2022; Wouters et al., 2016). This creates the need for automated correction measures to enable appropriate processing for all modalities. While performing virtual brain transplant (VBT), as implemented in this study, offers such a correction for anatomical modalities like T1w, T2, and FLAIR, the application to EPI sequences, such as fMRI and DWI, is less feasible for recovering the information distorted in the affected region. A more thorough investigation of how such lesion abnormalities affect these modalities must be performed. In the current study, we followed (Horbruegger et al., 2019) and excluded lesion signal from tractography by incorporating the corresponding lesion mask as pathological tissue in the context of anatomically constrained tractography, therefore introducing a potential bias by limiting the number of streamlines created around and at the lesion. Current research investigates potential avenues for integrating lesion voxels into tractography (Lipp et al., 2020) and will need to be integrated in automated pipelines such as LeAPP in the future once it provides robust stroke pathology specific approaches. While we did not perform specific corrections to the fMRI lesion signal, the lesion‐induced abnormality did affect our decision not to perform independent component analysis (ICA) for denoising and motion correction (e.g., FIX‐ICA (Salimi‐Khorshidi et al., 2014)). Previous studies have evaluated the robustness of such frameworks in the presence of stroke lesions (Carone et al., 2017) showing a significant signal loss when applying automated complex approaches. It was further shown that lesion‐based variance patterns differ significantly from healthy tissue, even allowing for component‐based lesion identification (Hu et al., 2021) in resting‐state fMRI. Further research into our understanding of signal and noise sources as identified by ICA at the focal lesion is needed in the presence of task‐based clinical fMRI data as present in this study.

Lesion mitigation measures integrated within LeAPP currently remain limited to localized correction related to the a‐priori provided lesion mask. This limitation reduces the ability of LeAPP to correct for distortions and diaschisis effects caused by the stroke and further research is needed to facilitate appropriate processing and analysis methods for incorporating distributed lesion impact beyond the focal lesion. Furthermore, the need for a‐priori provided masks requires time‐consuming lesion segmentation by trained staff or the use of additional lesion detection software before the pipeline can be utilized. However, a recent review (Akkus et al., 2017) found that automated segmentation algorithms perform not yet sufficiently well, to justify integration into fully automated pipelines. While algorithms for lesion segmentation do exist for use across diseases (Federau et al., 2020; Liu et al., 2021; Maier et al., 2015; Narayana et al., 2020) and species (An et al., 2023), and datasets are made available to enhance and validate these segmentation algorithms (Liew et al., 2018; Liew et al., 2022), we concluded that the performance for human patient stroke lesion segmentation is not yet at a level justifying the integration in a fully automated framework such as LeAPP.

### 4.4 Conclusions

In summary, we found that LeAPP represents significant progress for processing multi‐modal neuroimaging patient data with lesion pathologies and its level of automation makes this workflow readily available to the scientific community. The pipeline can be used as a standard—well documented and versioned—tool for the processing of stroke imaging data thus ensuring a high degree of reproducibility and comparability of results. This is an important advancement as it can aid future studies in processing large cohorts of stroke data. Furthermore, LeAPP generates and outputs brain networks in TVB‐ready formats that facilitate dynamic brain network modelling of virtual stroke brains with the TVB software. This will further strengthen and allow for simulation‐based research into underlying recovery mechanisms, that so far have not been well understood.

## AUTHOR CONTRIBUTIONS

**PB**: Conceptualization; data curation; formal analysis; investigation; methodology; software; validation; visualization; writing—original draft preparation; writing—review and editing. **KD**: Methodology; writing—review and editing. **AK**: writing—review and editing. **MS**: writing—review and editing. **JF**: Resources. **MB**: Resources. **RS**: Resources; writing—review and editing. **BC**: Resources; **GT**: Resources. **CG**: Resources. **PR**: Conceptualization; funding acquisition; supervision; writing—review and editing; management.

## FUNDING INFORMATION

This work was supported by the Virtual Research Environment at the Charité Berlin—a node of EBRAINS Health Data Cloud. Part of computation has been performed on the HPC for Research cluster of the Berlin Institute of Health. PR acknowledges support by EU Horizon Europe program Horizon EBRAINS2.0 (101147319), Virtual Brain Twin (101137289), EBRAINS‐PREP 101079717, AISN—101057655, EBRAIN‐Health 101058516, Digital Europe TEF‐Health 101100700, EU H2020 Virtual Brain Cloud 826421, Human Brain Project SGA2 785907; Human Brain Project SGA3 945539, ERC Consolidator 683049; German Research Foundation SFB 1436 (project ID 425899996); SFB 1315 (project ID 327654276); SFB 936 (project ID 178316478); SFB‐TRR 295 (project ID 424778381); SPP Computational Connectomics RI 2073/6‐1, RI 2073/10‐2, RI 2073/9‐1; DFG Clinical Research Group BECAUSE‐Y 504745852, PHRASE Horizon EIC grant 101058240; Berlin Institute of Health & Foundation Charité, Johanna Quandt Excellence Initiative; ERAPerMed Pattern‐Cog 2522FSB904. BC, GT, and CG acknowledge the following funding sources: German Research Foundation (178316478, project C1, 178316478, project C2).

## Supporting information


**Data S1:** Supporting Information.

**Data S2:** Supporting Information.

## Data Availability

Where applicable, this study fulfills the TRIPOD checklist (Table S7) for description of the development and validation of the pipeline. The authors declare that the source code required for replicating the present study is made freely available and the described software is disseminated via containerized packages for the processing and validation pipelines described in this article (www.github.com/brainmodes/LeAPP). Input and result (derivative) data will be made discoverable via the EBRAINS knowledge graph (https://search.kg.ebrains.eu/). Sharing of these data is subject to the EU General Data Protection Regulations (GDPR) and requires the establishment of a research purpose of processing, conclusion of EU standard contractual clauses between controllers and processors and a data protection impact assessment (DPIA) approved by the relevant institutional data protection officers.
