# Supplementary material for: A lesion‐aware automated processing framework for clinical stroke magnetic resonance imaging
Source: Hum Brain Mapp. 2024 Jun 22;45(9):e26701. doi: 10.1002/hbm.26701 (PMC11193092; doi:10.1002/hbm.26701)
Supplement: Supplementary file 1 — Data S1: Supporting Information. [file HBM-45-e26701-s001.docx]

# **SUPPLEMENTARY MATERIAL – METHODS & TABLES**

A lesion-aware automated processing framework for clinical stroke MRI

Patrik Bey^1,2^, Kiret Dhindsa^1,2^, Amrit Kashyap^1^, Michael Schirner^1,2,3,4,5^, Jan Feldheim^6^, Marlene Bönstrup^6(*)^, Robert Schulz^6^, Bastian Cheng^6^, Götz Thomalla^6^, Christian Gerloff^6^, Petra Ritter^1,2,3,4,5^

^1^Berlin Institute of Health at Charité – Universitätsmedizin Berlin, Berlin, Germany, ^2^ Department of Neurology with Experimental Neurology, Brain Simulation Section, Charité – Universitätsmedizin Berlin, corporate member of Freie Universität Berlin and Humboldt-Universität zu Berlin, Berlin, Germany, ^3^Bernstein Focus State Dependencies of Learning and Bernstein Center for Computational Neuroscience, Berlin, Germany, ^4^Einstein Center for Neuroscience Berlin, ^5^Einstein Center Digital Future, ^6^Klinik und Poliklinik für Neurologie, Kopf- und Neurozentrum, University Medical Center, Hamburg-Eppendorf, Germany, ^7^Klinik und Poliklinik für Neurologie, Universitätsklinikum Leipzig, Leipzig, Germany

**METHODS - Virtual Brain Transplant**

Following (Solodkin et al. 2010) we implemented virtual brain transplant as introduced in the Methods section of this study. **Figure S1** highlights the main interim steps for an example patient T1w image. The input T1w image is first mirrored using *fslspawdim* along the sagittal plane to create a reference image for midline alignment. The input image is linearly co-registered to the mirror image using *FSL flirt* 6 dof function with cost function masking for the input and reference image. The resulting transformation matrix is saved, and the half transformations are computed representing two consecutive warps, first from input to midline and second from midline to mirror image. The first halt-transform is applied to input image and input lesion mask creating midline aligned version of these volumes. The second half-transform is inversed and applied to the mirror image creating a midline aligned mirror image. The aligned lesion mask is smoothed using a Gaussian Kernel with 2mm full width at half maximum around the lesion border. The smoothed border is concatenated with the original lesion mask to create a full mask with smoothed outline. The smoothed lesion mask is used to extract healthy transplant from the aligned mirror image and inverted to extract the healthy signal from midline aligned input image. The lesion free image and the transplant image are concatenated creating a full brain volume. The inverse of the first half-transform, now warping from midline back to original input space, is applied to create a healthy approximation of
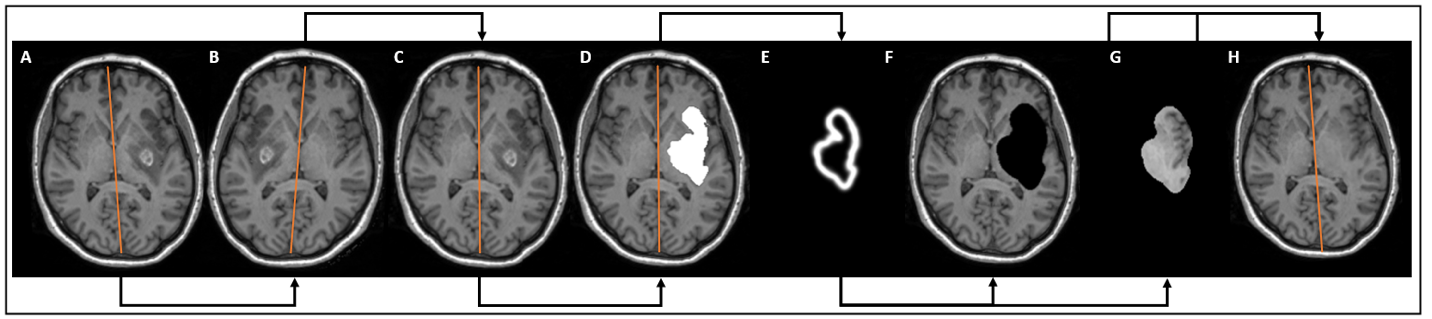
the underlying ground truth for the input patient image.

**Figure S1 Virtual Brain Transplant** Step wise description of the implementation of VBT in this study. First the lesioned input image (**A**) is mirrored (**B**) and registered to the mirror image. The resulting transformation matrix is saved, and the half-transformations computed and inversed. The first half transformation (from input space to midline) is applied to the input image and lesion mask (**C,D**) and the inverse second half transformation (mirror space to midline) to the mirror image. The warped lesion mask is smoothed by applying a Gaussian Kernel to the extracted lesion border (**E**) and concatenated with the binary lesion mask. This mask is used to remove lesion signal from the midline aligned input image (**F**) and extract healthy signal from the midline aligned mirror image (**G**). The resulting images are combined and finally the inverse first half transform (midline to input space) is applied for the final transplanted image in input space (**H**).

**METHODS – artificial lesion embedding quality control**

For the selection of meaningful artificial lesion embedded stroke patients, visual quality control was performed. Exclusion criteria consisted of unrealistic properties such as embedding within cerebrospinal fluid filled areas, the skull of the participant and similar unrealistic lesion manifestations not found in real ischemic stroke patient populations. The main reason for failed QC is the brain volume driven incompatibility of healthy controls and stroke patients. Large volume lesions might be more problematic to embed in healthy control brains with lower overall brain volume. **Figure S2** shows an example of a failed and passed QC for the same underlying healthy control ground truth brain volume.


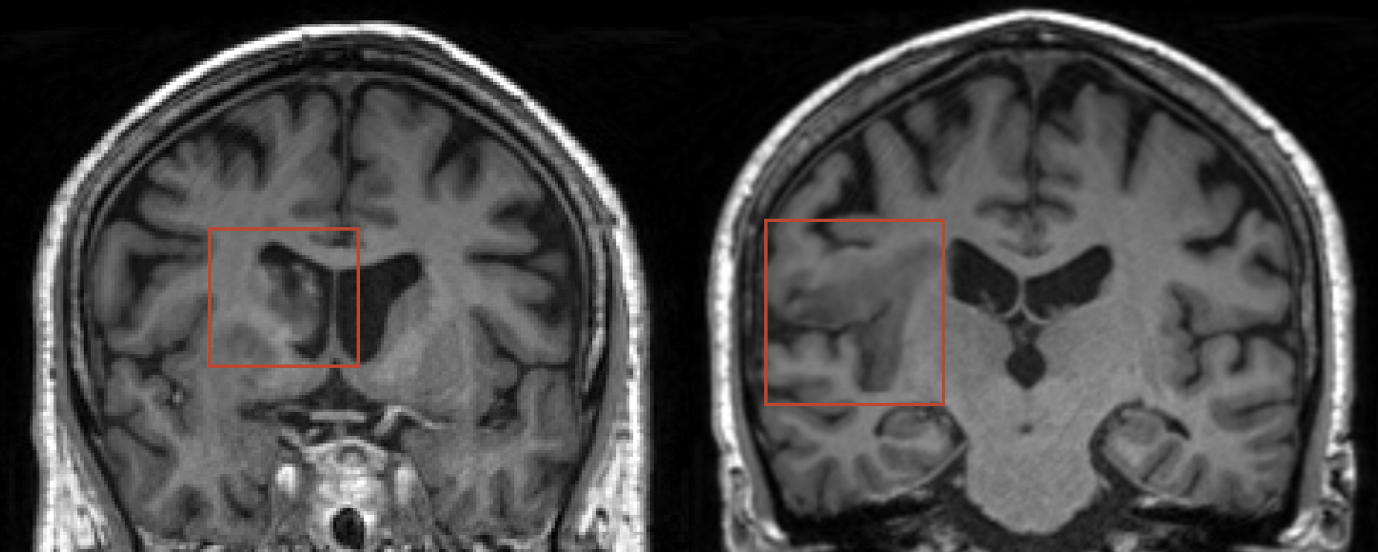


**Figure S2** Candidates for ALE patients from the creation of the validation data set. The failed quality control candidate (left) shows a clear embedding of lesion tissue within the right ventricle to a degree not found within real ischemic stroke patient populations. The successful QC candidate (right) represents a feasible ALE patient showing a lesion manifestation in cortical and subcortical tissue comparable to real stroke patient lesions found in the present cohort.

To investigate the impact of varying brain volumes we computed the brain volumes for patients and healthy controls as well as corresponding lesion volumes (**Figure S3**). We can see that while overall brain sizes are comparable between healthy controls and patients, due to the matching ages between groups, we see a total of 13 patients with brain sizes larger than the average healthy control brain size. This number is indicative of potential mismatches when randomly combining healthy controls and patients for artificial lesion embedding as performed in the present study.


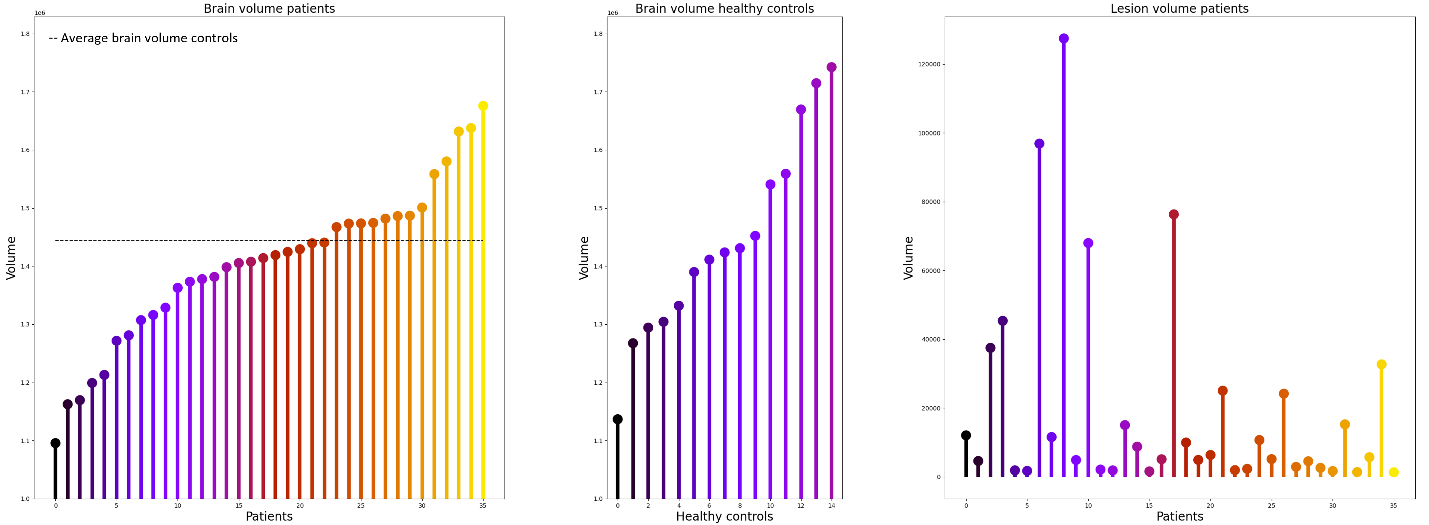


**Figure S3 Brain volumes** comparison between patient and healthy control population including lesion volumes.

**METHODS – Comparison with fMRIPrep processing pipeline**

To further validate the performance of LeAPP in processing multi-modal MRI data in the presence of stroke lesions compared to existing frameworks we also investigated the impact of lesion pathology when using the fmriprep (Esteban et al. 2019) processing pipeline. To this end we reused the previously introduced validation data set. Similar to the comparison with the HCP pipeline (Glasser et al. 2013) before we computed agreement measures between the ground truth and ALE patients processing pipeline outputs. As both functional and diffusion-based processing is dependent on the structural processing results we focus on the validation of structural processing as integrated in fmriprep and LeAPP.

In a first step we processed the existing validation data using the latest version of the fmriprep docker container available via dockerhub (poldracklab/fmriprep:latest). To enable comparison between LeAPP and the fmriprep based processing results we used the same parcellation mapping approach from LeAPP on the results created using fmriprep. As the latter is also based on FreeSurfer’s (Fischl 2012) surface reconstruction algorithm (recon-all) the necessary and equivalent files were readily available. The parcellation mapping performed afterwards only remaps the existing labels for the given parcellations created by FreeSurfer and does not therefore introduce any potential bias for the subsequent validation.

Using the created fmriprep-based parcellations, we again computed a range of agreement measures between the processed artificial stroke patients and the corresponding ground truth using the containerized validation framework introduced in the manuscript.


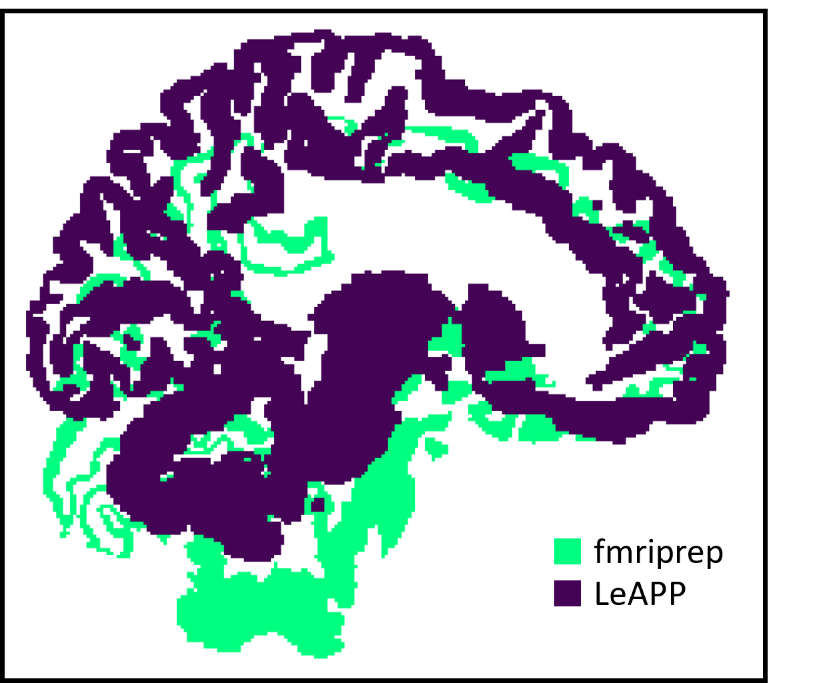
It is important to note that the results of fmriprep and LeAPP cannot be directly compared as they were between HCP and LeAPP in the manuscript. This is due to e.g. varying approaches in processing, for example different levels of complexity in the utilization of FreeSurfer’s surface reconstruction algorithm (a single continuous recon-all call during fmriprep compared to updated recon-all calls during HCP / LeAPP with additional interim processing steps The clear difference in processing results is shown in **Figure S4**, showing the processing result for the same ground truth data subject for both LeAPP and fmriprep.

We hypothesized that fmriprep would suffer from similar lesion pathology induced distortions during reconstruction of ground truth brain topology as the standard HCP pipeline, therefore leading to less accurate reconstruction of the ground truth compared to LeAPP. To assess the lesion impact on processing directly, we compared the average agreement for lesion affected and not-affected ROIs for each ALE patient and tested for reduced agreement. ROIs were defined as lesion-affected for the case of a non-zero lesion load, defined by the ratio of ROI voxels overlapping with the corresponding lesion mask.

**Figure S4** Comparison of final processing results for LeAPP and fmriprep for the same ground truth data subject.

**Results:**

**Figure S5** Ground truth comparison Processing results for fmriprep based comparison of ALE patients with ground truth healthy control data subject (left). Comparison with ground truth for both LeAPP and HCP as shown in the main manuscript (right). The binarized parcellation created with fmriprep shows similar distortion for the ALE patient due to abnormal lesion signal, compared to the standard HCP structural processing pipeline (right **B**) as performed during validation of LeAPP, highlighting the potential reduction in processing accuracy for fmriprep.

The processing of ALE patients with fmriprep showed similar performance compared to the HCP pipeline. The cortical ribbon created by FreeSurfer and used as the bases for individual brain parcellations is significantly distorted around the pathological lesion signal. The left column of **Figure S5** shows the **
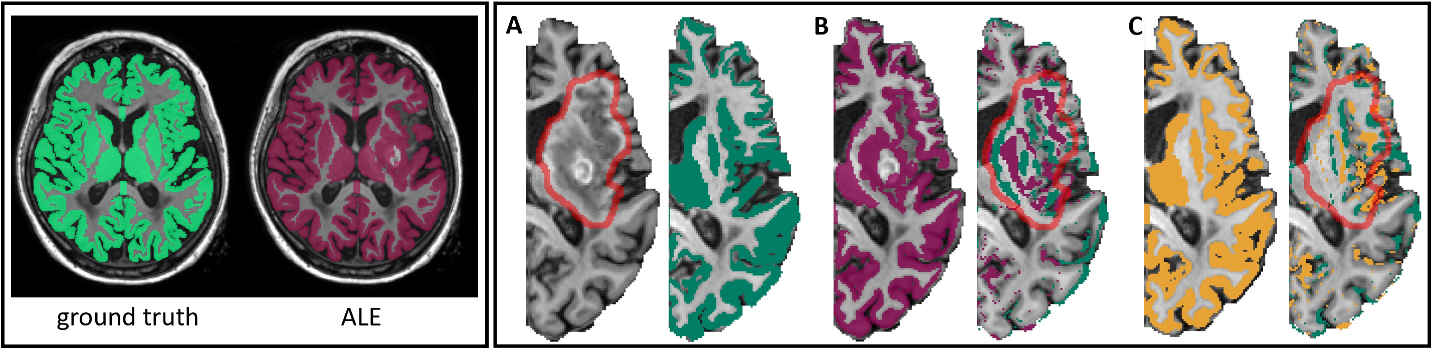
**binarized individual parcellation created using fmriprep for the underlying ground truth data subject (green) and for a corresponding ALE patient created from that specific ground truth participant (purple). As with HCP, we see a clear misclassification of voxels around the lesion, due to its abnormal intensity values (**Figure S5** (**B**)). **Figure S6** below shows the validation results across the three local agreement metrics dice coefficient, volume difference and distance of center-of-gravity, normalized to values between 0 and 1 for visualization purposes. In the case of fmriprep processing results (right), it shows a clear reduction in average agreement for ROIs that are directly affected by the lesion signal, similar to the reduction described in the manuscript for HCP pipeline (see **Table S1**). This impact is significantly reduced in LeAPP due to the additional integrated mitigation measures beyond cost function masking. The differences between average agreement values for not affected and affected ROIs is not significant for any measure in the case of LeAPP (p-values: dice=0.992, volume difference=0.22, distance=0.55) while significant for fmriprep (p-values: dice<0.0001, volume difference=0.047, distance<0.0001) see **Table S1**.

**Table S1** Local differences in agreement measures for fmriprep and LeAPP. Fmriprep shows a significant reduction in agreement metric for lesion affected ROIs while such differences are not significant for LeAPP.

| **Local Measure** | **Lesion affected** | **Not affected** | **p-value (statistic)** |
| --- | --- | --- | --- |
| **fmriprep** | | | |
| Dice | 0.75 (0.028) | 0.87 (0.0007) | <0.001 (-6.56) |
| Distance | 2.52 (4.96) | 0.87 (0.13) | 0.047 (1.69) |
| Volume | 0.007 (0.0035) | -0.004 (0.0001) | <0.001 (7.26) |
| **LeAPP** | | | |
| Dice | 0.84 (0.01) | 0.82 (0.01) | 0.99 (2.45) |
| Distance | 0.791 (0.376) | 0.798 (0.217) | 0.55 (-0.13) |
| Volume | -0.003 (0.002) | -0.008 (0.002) | 0.22 (0.78) |


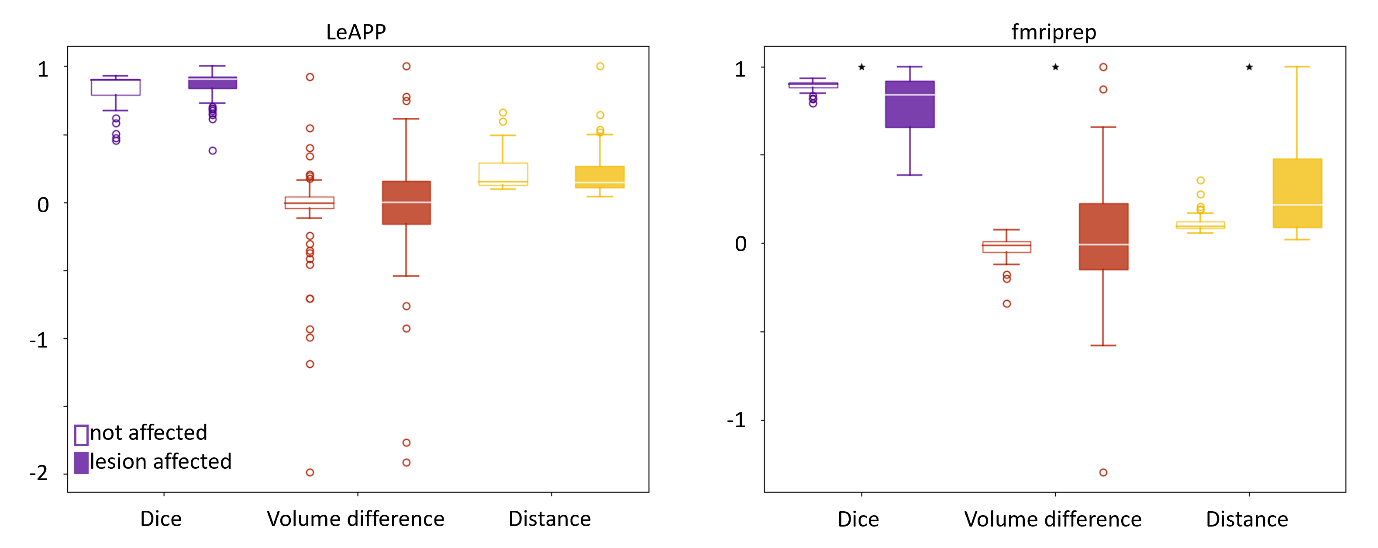


**Figure S6** The local impact of lesion pathology on agreement metrics between artificial stroke patients and the corresponding ground truth volumes. The shown measures are computed on a per region-of-interest (ROI) basis and grouped by direct versus no direct impact of lesion pathology. Lesion impact was defined as overlap of the ROI mask with the lesion mask. While we see larger variance overall in the LeAPP results (left) compared to fmriprep (right), potentially due to the increased complexity of processing in LeAPP, we see a significant reduction of agreement values for fmriprep from non-affected to lesion-affected ROIs, while this is not present in LeAPP. Dice scores represent a high agreement, i.e. reconstruction quality of the ground truth, the closer the value is to one. Volume difference and distance show high agreement, the closer the value is to zero. Volume difference can be positive and negative depending on the direction of the size difference compared to the ground truth.

To further highlight the local impact of lesion pathology in fmriprep processing **Figure S7** shows the same exemplary artificial stroke patient used for visualization in the main manuscript (**Figure 6**). It displays the ROI based agreement measures for all three processing results (LeAPP, HCP and fmriprep) with colormaps rescaled to the overall maximum value across results to enable comparability.


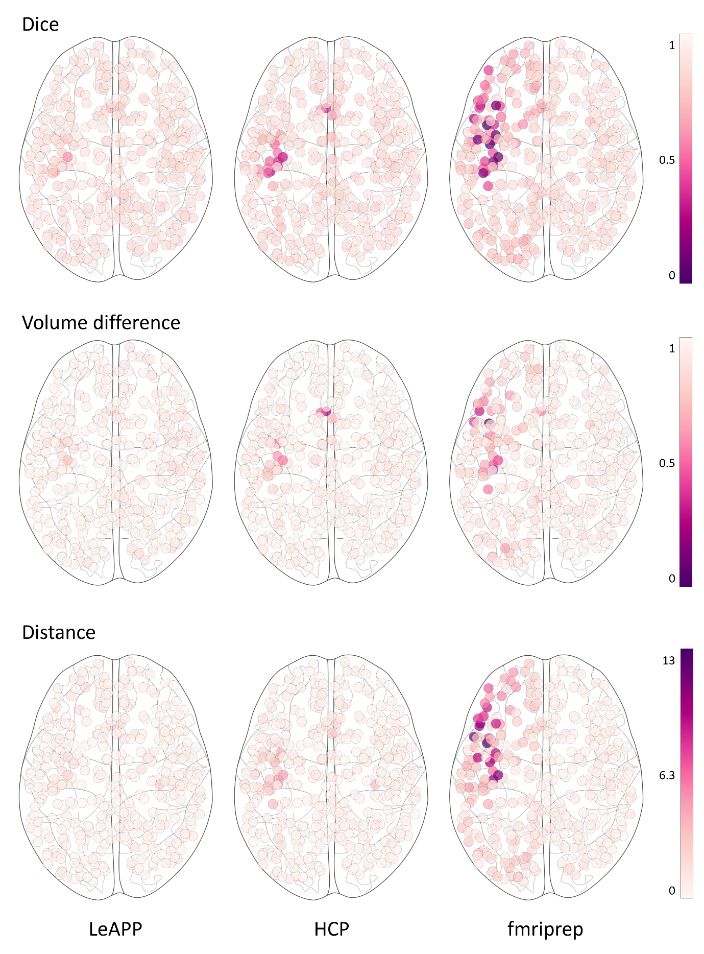
While fmriprep and other frameworks provide important functionalities to the scientific community, we could show the necessity to further extend such existing approaches for the best results when processing stroke lesion MRI data as provided with LeAPP. LeAPP therefore represents a major advancement, built upon prior contributions, towards comprehensive and standardized processing of patient MRI data with stroke pathologies.

**Figure S7** Example of local agreement across processing results for a single artificial lesion patient. All agreement measures show a high localized impact of lesion abnormality for HCP and fmriprep processing pipelines, with fmriprep showing the largest deviations from ground truth. Values were rescaled to the maximum value within each agreement metric across processing frameworks for visualization.

**METHODS - Diffusion weighted processing pipeline**

The full list of processing steps performed during diffusion pipeline is given in **Table S2**.

**Table S2 Diffusion processing steps**

| **Step** | **Processing** |
| --- | --- |
| 1. | Denoising |
| 2. | Degibssing |
| 3. | Dwipreproc |
| 4. | Distortion correction (by registration to T1w space) |
| 5. | Biascorrection |
| 6. | Intensity normalization |
| 7. | Five tissue type segmentation |
| 8. | Lesion embedding in 5tt |
| 9. | Response function |
| 10. | Average response function |
| 11. | Fiber Orientation Distribution tractography with GmWmI seed |
| 12. | Anatomically constrained tractography |
| 13. | Spherical-deconvolution informed filtering of tracks (sift2 - algorithm) |
| 14. | Connectome creation |

Processing steps as implemented in diffusion pipeline of the LeAPP framework of this study. Steps one to five concern general preprocessing (**Figure 1 (C)**) on individual subject level. Intensity normalization in step six is performed on population level to ensure group level comparability of intensity values. Five tissue type segmentation is performed on the fully processed T1w image output of the structural pipeline. Steps nine and ten represent the computation of response function for each subject and the averaging on group level. Based on the prior results tractography is performed in steps eleven and twelve before the connectome creation is completed in steps 13 and 14.

**METHODS – Magnetic resonance imaging acquisition parameters**

MRI data acquisition parameters of the present cohort has been previously described in (Schlemm et al. 2020; Schulz et al. 2016). We list them here in **Table S3** again for completeness.

**Table S3** MRI acquisition parameters The following abbreviations have been used in the present table describing the MRI acquisition parameters as used for the present cohort: TR: repetition time, TE: echo time, FOV: field-of-view, ST: slice thickness, IPR: in-plane resolution,

| **Parameter** | **Value** |
| --- | --- |
| Scanner | 3T Siemens Skyra (Siemens, Erlangen, Germany) |
| Head coul | 32 channel |
| **3D magnetization-prepared, rapid acquisition gradient-echo sequence (MPRAGE)** | |
| TR | 2500 ms |
| TE | 2.12 ms |
| FOV | 240 x 192 mm^2^ |
| Axial slice count | 256 |
| ST | 0.94 mm |
| IPR | 0.94 x 0.94 mm^2^ |
| **Fluid Attenuated Inversion Recovery (FLAIR)** | |
| TR | 9000 ms |
| TE | 90 ms |
| TI | 2500 ms |
| FOV | 230 x 230 mm^2^ |
| ST | 5 mm |
| IPR | 0.7 x 0.7 mm^2^ |
| Sequence |  |
| **Diffusion weighted Imaging** | |
| b | 1500 s/mm^2^ |
| directions | 64 |
| TR | 10000 ms |
| TE | 82 ms |
| FOV | 256 x 204 |
| ST | 2 mm |
| IPR | 2 x 2 mm^2^ |
| **Gradient Echo Planar Imaging** | |
| TR | 2000 ms |
| TE | 36 ms |
| FOV | 216 mm |
| ST | 3 mm |
| IPR | 2.3 x 2.3 mm^2^ |
| Flip angle | 90 degrees |
| Axial slice count | 26 |
| Slice range | Apex to Sylvian fissure |
| Volumes | 276 |

**METHODS – Reconstruction quality**

To estimate reconstruction quality across processing pipelines performed in the validation of the current study, we used a range of established measures capturing different properties of the underlying parcellation and connectome. For all local measures a separate analysis for lesion affected and non-lesion affected ROIs was performed to further investigate the local impact of the lesion.

**Agreement measures** aim to estimate the similarity of whole brain segmentations or individual regions-of-interest (ROIs). These measures represent the similarity between two objects (e.g. LeAPP based and ground truth segmentation) as their dyadic relationship resulting in a single value. Agreement measures can display limitations in accuracy and applicability regarding size and shapes of the corresponding objects. Following (Bertels et al. 2019) the present agreement measures were selected to account for such limitations and present a wholistic estimate of similarities.

*Dice coefficients* capture the overall overlap between two objects. This widely used metric (Taha and Hanbury 2015) enables a direct comparison of the general properties of the objects. It reports the ratio of the overlap of the objects over the sizes of both objects combined. Dice scores can be susceptible to minor differences along the contour of an object, depending on their overall size. This can result in similar values across comparisons with different properties.

*Jaccard score* is another overlap-based measure that is highly correlated with dice coefficients. It reports the ratio of the overlap over the size of the union of both objects. This can result in different value ranges compared to dice scores (Taha and Hanbury 2015), providing potentially additional validation information.

*Volume difference* was further evaluated to estimate the overall similarity in the sizes of the objects without incorporating the actual overlap (Taha and Hanbury 2015). It was defined as the difference in voxel count of each object with the size of the LeAPP or HCP object being subtracted from the ground truth size.

*Center-of-gravity* distance was computed via the Euclidean distance in 3D space between the center-of-gravity (cog) of both objects. The cog was computed as the mean coordinates of all voxels included in each object allowing for comparison in the case of non-continuous objects as well. It allows for a comparison of the general location of a given object independent of the shape of the object.

**Network measures** were defined as the difference in values of a given network metric for two connectomes. This enabled validation of network level differences for both processing pipelines. Where applicable differences in measures were computed on a local node level as well as global level for the full network. Definitions of network metrics used here follow (Bullmore and Sporns 2009).

*Node strength* was computed as the overall weighted strengths of connections incident to a given node. This value was averaged for the global network comparison. It allows for a general validation of the connectedness of a node to all other nodes in the network.

*Node centrality* was computed as the fraction of shortest paths of the network that run through the given node. This approximates the overall role of a given node as a crucial information hub within a given network (Bullmore and Sporns 2009).It was averaged for estimation of the global network measure.

*Clustering coefficients* were computed for all connectomes as an estimate of the overall structure of the networks. This metric captures the existence of local processing clusters as found in complex natural networks (Bullmore and Sporns 2009) such as structural connectomes of human brains.

**TABLES – Containerized processing modules**

**Table S4** Containerized processing modules Processing submodules callable via docker run command for containerized processing framework LeAPP.

| **Module** | **Requirements** | **Processing steps** |
| --- | --- | --- |
| *full* | None | Perform all implemented processing steps |
| *vbt* | Image volume  Lesion mask volume | Only performing virtual brain transplant |
| *lesion* | Lesion mask volume | Include lesion mitigation measure for all running processing steps |
| *nocleanup* | None | Do not remove temporary interim results |
| **Structural imaging modules** | | |
| *all* | None | All structural processing steps |
| *prefs* | None | Adjusted PreFreeSurfer step |
| *fs* | *prefs* results | Adjusted FreeSurfer step |
| *postfs* | *fs* results | Adjusted PostFreeSurfer step |
| *parcmap* | *postfs* results | Creating individual parcellation mapping based on PostFreeSurfer results |
| **Functional imaging moduls** | | |
| *all* | *parcmap* results | All functional processing steps |
| *preproc* | *parcmap* results | Adjusted fMRIVolume step |
| *connectome* | *preproc* results | Functional connectome creation |
| **Diffusion imaging modules** | | |
| *preproc* | *parcmap* results | DWI preprocessing (see **Table S1**) |
| *normal* | *preproc* results | Population based intensity normalization |
| *segment* | *normal* results | Five tissue type segmentation |
| *response* | *normal* results | Response function normalization |
| *connectome* | *response* results | Anatomically constrained tractography and connectome creation |

**TABLES - Data availability**

**Table S5 Data overview** Description of all included participants following a complete data set at time point 1 as described in the manuscript. Lesion volume defines the volume of lesion masks at acute phase and was extracted using fslstats. Values of “nan” describe missing data.

| **Subject** | **Age** | **Sex** | **Lesion Volume** | **Timepoint 1** | **Timepoint 2** | **Timepoint 3** | **Timepoint 4** |
| --- | --- | --- | --- | --- | --- | --- | --- |
| **patients** | | | | | | | |
| P007 | 62 | m | 3555,68 | complete | complete | complete | complete |
| P009 | 49 | w | 53721,63 | complete | complete | complete | complete |
| P012 | 69 | m | 25072,61 | complete | complete | complete | complete |
| P015 | 65 | m | 2750,63 | complete | complete | complete | complete |
| P016 | 73 | w | 26764,17 | complete | complete | complete | complete |
| P019 | 54 | m | 465,11 | complete | - | - | - |
| P020 | 73 | w | 5816,34 | complete | complete | complete | complete |
| P022 | 70 | m | 5477,26 | complete | - | complete | - |
| P023 | 77 | w | 9099,10 | complete | complete | - | - |
| P024 | 53 | m | 2187,50 | complete | complete | - | complete |
| P025 | 81 | w | 790,18 | complete | complete | complete | complete |
| P026 | 78 | w | 839,19 | complete | complete | complete | complete |
| P027 | 85 | w | 4988,15 | complete | - | - | - |
| P030 | 56 | w | 1265,29 | complete | complete | complete | complete |
| P031 | 72 | w | 950,22 | complete | complete | complete | complete |
| P032 | 49 | m | 1806,42 | complete | complete | complete | - |
| P033 | 63 | m | 842,19 | complete | complete | complete | complete |
| P034 | 70 | w | 74374,16 | complete | complete | complete | complete |
| P035 | 65 | m | 6569,52 | complete | complete | complete | complete |
| P036 | 85 | w | 16750,87 | complete | - | complete | complete |
| P037 | 79 | m | 2786,64 | complete | - | - | - |
| P038 | 78 | w | 1783,41 | complete | - | - | - |
| P039 | 78 | w | 2459,57 | complete | - | - | - |
| P041 | 81 | m | 579,13 | complete | complete | complete | complete |
| P042 | 76 | m | 1106,26 | complete | complete | - | - |
| P043 | 44 | m | 85870,81 | complete | complete | complete | complete |
| P044 | 78 | w | 722,17 | complete | - | - | - |
| P045 | 47 | w | 6991,61 | complete | complete | complete | complete |
| P046 | 54 | m | 1109,26 | complete | - | complete | complete |
| P048 | 77 | w | 5852,35 | complete | - | - | - |
| P053 | 87 | w | 987,23 | complete | complete | - | - |
| P055 | 47 | m | 2577,59 | complete | - | - | complete |
| P056 | 59 | m | 14342,31 | complete | complete | - | - |
| P057 | 50 | m | 50074,55 | complete | complete | - | - |
| P058 | 78 | w | 28226,51 | complete | - | - | - |
| P059 | 51 | m | 844,19 | complete | - | - | - |
| **healthy controls** | | | | | | | |
| H001 | 68 | m |  | complete | - | - | - |
| H002 | 72 | w |  | complete | - | - | - |
| H003 | 73 | m |  | complete | - | - | - |
| H004 | 79 | m |  | complete | - | - | - |
| H006 | 78 | w |  | complete | - | - | - |
| H008 | 53 | m |  | complete | - | - | - |
| H009 | 74 | w |  | complete | - | - | - |
| H010 | 76 | w |  | complete | - | - | - |
| H011 | 68 | w |  | complete | - | - | - |
| H012 | 76 | m |  | complete | - | - | - |
| H014 | nan | w |  | complete | - | - | - |
| H015 | 68 | w |  | complete | - | - | - |
| H018 | 69 | m |  | complete | - | - | - |
| H020 | 59 | m |  | complete | - | - | - |
| H023 | nan | nan |  | complete | - | - | - |

Data was acquired over four timepoints (3-5 days, 30-40 days, 85-95 days and 340-380 days post onset). The participants in this study were selected based on data completeness for all brain imaging modalities as required (**Figure 3**) to test full LeAPP pipeline functionality. A full description of data acquisition and experimental task design can be found in supplementary material of ^5^. One patient’s complete data set as well as timepoints from two patients had to be excluded due to failed quality assessment (QA). The failure of processing resulted in the inability of the software packages to register the corresponding T1w images, resulting in significantly distorted and rotated image volumes. After an extensive search it was not possible to determine the underlying issue causing this failure, but any lesion specific impact can be precluded as e.g. a previous timepoint of the same patient was processed successfully.

**TABLES - Parcellation mapping regions of interest**

The following list contains all included ROIs based on the HCP-MMP1 brain parcellation (Glasser et al. 2016) and subcortical FreeSurfer segmentations (Fischl 2012) that were combined in this study to accurately capture full lesion extend. For the mapping of the resulting cortical labels, we followed a previously described implementation (CJ Neurolab 2018) and added the existing subcortical labels from FreeSurfer’s segmentation.

**Table S6** Regions of interest

| **ID** | **Label** | **ID** | **Label** | **ID** | **Label** | **ID** | **Label** | **ID** | **Label** | **ID** | **Label** | **ID** | **Label** | **ID** | **Label** |
| --- | --- | --- | --- | --- | --- | --- | --- | --- | --- | --- | --- | --- | --- | --- | --- |
| 1 | lh.L_V1 | 51 | lh.L_1 | 101 | lh.L_OP1 | 151 | lh.L_V6A | 201 | rh.R_PIT | 251 | rh.R_10d | 301 | rh.R_STGa | 351 | rh.R_MBelt |
| 2 | lh.L_MST | 52 | lh.L_2 | 102 | lh.L_OP2-3 | 152 | lh.L_VMV1 | 202 | rh.R_MT | 252 | rh.R_8C | 302 | rh.R_PBelt | 352 | rh.R_LBelt |
| 3 | lh.L_V6 | 53 | lh.L_3a | 103 | lh.L_52 | 153 | lh.L_VMV3 | 203 | rh.R_A1 | 253 | rh.R_44 | 303 | rh.R_A5 | 353 | rh.R_A4 |
| 4 | lh.L_V2 | 54 | lh.L_6d | 104 | lh.L_RI | 154 | lh.L_PHA2 | 204 | rh.R_PSL | 254 | rh.R_45 | 304 | rh.R_PHA1 | 354 | rh.R_STSva |
| 5 | lh.L_V3 | 55 | lh.L_6mp | 105 | lh.L_PFcm | 155 | lh.L_V4t | 205 | rh.R_SFL | 255 | rh.R_47l | 305 | rh.R_PHA3 | 355 | rh.R_TE1m |
| 6 | lh.L_V4 | 56 | lh.L_6v | 106 | lh.L_PoI2 | 156 | lh.L_FST | 206 | rh.R_PCV | 256 | rh.R_a47r | 306 | rh.R_STSda | 356 | rh.R_PI |
| 7 | lh.L_V8 | 57 | lh.L_p24pr | 107 | lh.L_TA2 | 157 | lh.L_V3CD | 207 | rh.R_STV | 257 | rh.R_6r | 307 | rh.R_STSdp | 357 | rh.R_a32pr |
| 8 | lh.L_4 | 58 | lh.L_33pr | 108 | lh.L_FOP4 | 158 | lh.L_LO3 | 208 | rh.R_7Pm | 258 | rh.R_IFJa | 308 | rh.R_STSvp | 358 | rh.R_p24 |
| 9 | lh.L_3b | 59 | lh.L_a24pr | 109 | lh.L_MI | 159 | lh.L_VMV2 | 209 | rh.R_7m | 259 | rh.R_IFJp | 309 | rh.R_TGd |  |  |
| 10 | lh.L_FEF | 60 | lh.L_p32pr | 110 | lh.L_Pir | 160 | lh.L_31pd | 210 | rh.R_POS1 | 260 | rh.R_IFSp | 310 | rh.R_TE1a | 359 | lh.Cerebellum |
| 11 | lh.L_PEF | 61 | lh.L_a24 | 111 | lh.L_AVI | 161 | lh.L_31a | 211 | rh.R_23d | 261 | rh.R_IFSa | 311 | rh.R_TE1p | 360 | lh.Thalamus |
| 12 | lh.L_55b | 62 | lh.L_d32 | 112 | lh.L_AAIC | 162 | lh.L_VVC | 212 | rh.R_v23ab | 262 | rh.R_p9-46v | 312 | rh.R_TE2a | 361 | lh.Caudate |
| 13 | lh.L_V3A | 63 | lh.L_8BM | 113 | lh.L_FOP1 | 163 | lh.L_25 | 213 | rh.R_d23ab | 263 | rh.R_46 | 313 | rh.R_TF | 362 | lh.Putamen |
| 14 | lh.L_RSC | 64 | lh.L_p32 | 114 | lh.L_FOP3 | 164 | lh.L_s32 | 214 | rh.R_31pv | 264 | rh.R_a9-46v | 314 | rh.R_TE2p | 363 | lh.Pallidum |
| 15 | lh.L_POS2 | 65 | lh.L_10r | 115 | lh.L_FOP2 | 165 | lh.L_pOFC | 215 | rh.R_5m | 265 | rh.R_9-46d | 315 | rh.R_PHT | 364 | Brain-Stem |
| 16 | lh.L_V7 | 66 | lh.L_47m | 116 | lh.L_PFt | 166 | lh.L_PoI1 | 216 | rh.R_5mv | 266 | rh.R_9a | 316 | rh.R_PH | 365 | lh.Hippocampus |
| 17 | lh.L_IPS1 | 67 | lh.L_8Av | 117 | lh.L_AIP | 167 | lh.L_Ig | 217 | rh.R_23c | 267 | rh.R_10v | 317 | rh.R_TPOJ1 | 366 | lh.Amygdala |
| 18 | lh.L_FFC | 68 | lh.L_8Ad | 118 | lh.L_EC | 168 | lh.L_FOP5 | 218 | rh.R_5L | 268 | rh.R_a10p | 318 | rh.R_TPOJ2 | 367 | lh.Accumbens |
| 19 | lh.L_V3B | 69 | lh.L_9m | 119 | lh.L_PreS | 169 | lh.L_p10p | 219 | rh.R_24dd | 269 | rh.R_10pp | 319 | rh.R_TPOJ3 | 368 | lh.VentralDC |
| 20 | lh.L_LO1 | 70 | lh.L_8BL | 120 | lh.L_ProS | 170 | lh.L_p47r | 220 | rh.R_24dv | 270 | rh.R_11l | 320 | rh.R_DVT | 369 | rh.Cerebellum |
| 21 | lh.L_LO2 | 71 | lh.L_9p | 121 | lh.L_PeEc | 171 | lh.L_TGv | 221 | rh.R_7AL | 271 | rh.R_13l | 321 | rh.R_PGp | 370 | rh.Thalamus |
| 22 | lh.L_PIT | 72 | lh.L_10d | 122 | lh.L_STGa | 172 | lh.L_MBelt | 222 | rh.R_SCEF | 272 | rh.R_OFC | 322 | rh.R_IP2 | 371 | rh.Caudate |
| 23 | lh.L_MT | 73 | lh.L_8C | 123 | lh.L_PBelt | 173 | lh.L_LBelt | 223 | rh.R_6ma | 273 | rh.R_47s | 323 | rh.R_IP1 | 372 | rh.Putamen |
| 24 | lh.L_A1 | 74 | lh.L_44 | 124 | lh.L_A5 | 174 | lh.L_A4 | 224 | rh.R_7Am | 274 | rh.R_LIPd | 324 | rh.R_IP0 | 373 | rh.Pallidum |
| 25 | lh.L_PSL | 75 | lh.L_45 | 125 | lh.L_PHA1 | 175 | lh.L_STSva | 225 | rh.R_7PL | 275 | rh.R_6a | 325 | rh.R_PFop | 374 | rh.Hippocampus |
| 26 | lh.L_SFL | 76 | lh.L_47l | 126 | lh.L_PHA3 | 176 | lh.L_TE1m | 226 | rh.R_7PC | 276 | rh.R_i6-8 | 326 | rh.R_PF | 375 | rh.Amygdala |
| 27 | lh.L_PCV | 77 | lh.L_a47r | 127 | lh.L_STSda | 177 | lh.L_PI | 227 | rh.R_LIPv | 277 | rh.R_s6-8 | 327 | rh.R_PFm | 376 | rh.Accumbens |
| 28 | lh.L_STV | 78 | lh.L_6r | 128 | lh.L_STSdp | 178 | lh.L_a32pr | 228 | rh.R_VIP | 278 | rh.R_43 | 328 | rh.R_PGi | 377 | rh.VentralDC |
| 29 | lh.L_7Pm | 79 | lh.L_IFJa | 129 | lh.L_STSvp | 179 | lh.L_p24 | 229 | rh.R_MIP | 279 | rh.R_OP4 | 329 | rh.R_PGs | 378 | Fornix |
| 30 | lh.L_7m | 80 | lh.L_IFJp | 130 | lh.L_TGd | 180 | rh.R_V1 | 230 | rh.R_1 | 280 | rh.R_OP1 | 330 | rh.R_V6A | 379 | CC_Posterior |
| 31 | lh.L_POS1 | 81 | lh.L_IFSp | 131 | lh.L_TE1a | 181 | rh.R_MST | 231 | rh.R_2 | 281 | rh.R_OP2-3 | 331 | rh.R_VMV1 | 380 | CC_Mid_Posterior |
| 32 | lh.L_23d | 82 | lh.L_IFSa | 132 | lh.L_TE1p | 182 | rh.R_V6 | 232 | rh.R_3a | 282 | rh.R_52 | 332 | rh.R_VMV3 | 381 | CC_Central |
| 33 | lh.L_v23ab | 83 | lh.L_p9-46v | 133 | lh.L_TE2a | 183 | rh.R_V2 | 233 | rh.R_6d | 283 | rh.R_RI | 333 | rh.R_PHA2 | 382 | CC_Mid_Anterior |
| 34 | lh.L_d23ab | 84 | lh.L_46 | 134 | lh.L_TF | 184 | rh.R_V3 | 234 | rh.R_6mp | 284 | rh.R_PFcm | 334 | rh.R_V4t | 383 | CC_Anterior |
| 35 | lh.L_31pv | 85 | lh.L_a9-46v | 135 | lh.L_TE2p | 185 | rh.R_V4 | 235 | rh.R_6v | 285 | rh.R_PoI2 | 335 | rh.R_FST |  |  |
| 36 | lh.L_5m | 86 | lh.L_9-46d | 136 | lh.L_PHT | 186 | rh.R_V8 | 236 | rh.R_p24pr | 286 | rh.R_TA2 | 336 | rh.R_V3CD |  |  |
| 37 | lh.L_5mv | 87 | lh.L_9a | 137 | lh.L_PH | 187 | rh.R_4 | 237 | rh.R_33pr | 287 | rh.R_FOP4 | 337 | rh.R_LO3 |  |  |
| 38 | lh.L_23c | 88 | lh.L_10v | 138 | lh.L_TPOJ1 | 188 | rh.R_3b | 238 | rh.R_a24pr | 288 | rh.R_MI | 338 | rh.R_VMV2 |  |  |
| 39 | lh.L_5L | 89 | lh.L_a10p | 139 | lh.L_TPOJ2 | 189 | rh.R_FEF | 239 | rh.R_p32pr | 289 | rh.R_Pir | 339 | rh.R_31pd |  |  |
| 40 | lh.L_24dd | 90 | lh.L_10pp | 140 | lh.L_TPOJ3 | 190 | rh.R_PEF | 240 | rh.R_a24 | 290 | rh.R_AVI | 340 | rh.R_31a |  |  |
| 41 | lh.L_24dv | 91 | lh.L_11l | 141 | lh.L_DVT | 191 | rh.R_55b | 241 | rh.R_d32 | 291 | rh.R_AAIC | 341 | rh.R_VVC |  |  |
| 42 | lh.L_7AL | 92 | lh.L_13l | 142 | lh.L_PGp | 192 | rh.R_V3A | 242 | rh.R_8BM | 292 | rh.R_FOP1 | 342 | rh.R_25 |  |  |
| 43 | lh.L_SCEF | 93 | lh.L_OFC | 143 | lh.L_IP2 | 193 | rh.R_RSC | 243 | rh.R_p32 | 293 | rh.R_FOP3 | 343 | rh.R_s32 |  |  |
| 44 | lh.L_6ma | 94 | lh.L_47s | 144 | lh.L_IP1 | 194 | rh.R_POS2 | 244 | rh.R_10r | 294 | rh.R_FOP2 | 344 | rh.R_pOFC |  |  |
| 45 | lh.L_7Am | 95 | lh.L_LIPd | 145 | lh.L_IP0 | 195 | rh.R_V7 | 245 | rh.R_47m | 295 | rh.R_PFt | 345 | rh.R_PoI1 |  |  |
| 46 | lh.L_7PL | 96 | lh.L_6a | 146 | lh.L_PFop | 196 | rh.R_IPS1 | 246 | rh.R_8Av | 296 | rh.R_AIP | 346 | rh.R_Ig |  |  |
| 47 | lh.L_7PC | 97 | lh.L_i6-8 | 147 | lh.L_PF | 197 | rh.R_FFC | 247 | rh.R_8Ad | 297 | rh.R_EC | 347 | rh.R_FOP5 |  |  |
| 48 | lh.L_LIPv | 98 | lh.L_s6-8 | 148 | lh.L_PFm | 198 | rh.R_V3B | 248 | rh.R_9m | 298 | rh.R_PreS | 348 | rh.R_p10p |  |  |
| 49 | lh.L_VIP | 99 | lh.L_43 | 149 | lh.L_PGi | 199 | rh.R_LO1 | 249 | rh.R_8BL | 299 | rh.R_ProS | 349 | rh.R_p47r |  |  |
| 50 | lh.L_MIP | 100 | lh.L_OP4 | 150 | lh.L_PGs | 200 | rh.R_LO2 | 250 | rh.R_9p | 300 | rh.R_PeEc | 350 | rh.R_TGv |  |  |

Regions of interest for the created parcellation combining HCP-MMP1 and FreeSurfer subcortical areas. Note the inclusion of left and right hemisphere hippocampus for a more accurate segmentation as compared to HCP-MMP1 template used in this study (Kathryn Mills 2016).

**TABLES - TRIPOD checklist**

**Table S7: TRIPOD Checklist: Prediction Model Development and Validation**

| **Section/Topic** | **Item** |  | **Checklist Item** | **Page** |
| --- | --- | --- | --- | --- |
| **Title and abstract** | | | | |
| Title | 1 | D;V | Identify the study as developing and/or validating a multivariable prediction model, the target population, and the outcome to be predicted. | 1 |
| Abstract | 2 | D;V | Provide a summary of objectives, study design, setting, participants, sample size, predictors, outcome, statistical analysis, results, and conclusions. | 1 |
| **Introduction** | | | | |
| Background and objectives | 3a | D;V | Explain the medical context (including whether diagnostic or prognostic) and rationale for developing or validating the multivariable prediction model, including references to existing models. | 3 |
|  | 3b | D;V | Specify the objectives, including whether the study describes the development or validation of the model or both. | 3 |
| **Methods** | | | | |
| Source of data | 4a | D;V | Describe the study design or source of data (e.g., randomized trial, cohort, or registry data), separately for the development and validation data sets, if applicable. | 3-4 |
|  | 4b | D;V | Specify the key study dates, including start of accrual; end of accrual; and, if applicable, end of follow-up. | 4 |
| Participants | 5a | D;V | Specify key elements of the study setting (e.g., primary care, secondary care, general population) including number and location of centres. | 4 |
|  | 5b | D;V | Describe eligibility criteria for participants. | 4 |
|  | 5c | D;V | Give details of treatments received, if relevant. | NA |
| Outcome | 6a | D;V | Clearly define the outcome that is predicted by the prediction model, including how and when assessed. | 6-8 |
|  | 6b | D;V | Report any actions to blind assessment of the outcome to be predicted. | NA |
| Predictors | 7a | D;V | Clearly define all predictors used in developing or validating the multivariable prediction model, including how and when they were measured. | NA |
|  | 7b | D;V | Report any actions to blind assessment of predictors for the outcome and other predictors. | NA |
| Sample size | 8 | D;V | Explain how the study size was arrived at. | 7 |
| Missing data | 9 | D;V | Describe how missing data were handled (e.g., complete-case analysis, single imputation, multiple imputation) with details of any imputation method. | NA |
| Statistical analysis methods | 10a | D | Describe how predictors were handled in the analyses. | NA |
|  | 10b | D | Specify type of model, all model-building procedures (including any predictor selection), and method for internal validation. | 6-8 |
|  | 10c | V | For validation, describe how the predictions were calculated. | 7-8 |
|  | 10d | D;V | Specify all measures used to assess model performance and, if relevant, to compare multiple models. | 6-8 |
|  | 10e | V | Describe any model updating (e.g., recalibration) arising from the validation, if done. | NA |
| Risk groups | 11 | D;V | Provide details on how risk groups were created, if done. | NA |
| Development vs. validation | 12 | V | For validation, identify any differences from the development data in setting, eligibility criteria, outcome, and predictors. | NA |
| **Results** | | | | |
| Participants | 13a | D;V | Describe the flow of participants through the study, including the number of participants with and without the outcome and, if applicable, a summary of the follow-up time. A diagram may be helpful. | Table S2 |
|  | 13b | D;V | Describe the characteristics of the participants (basic demographics, clinical features, available predictors), including the number of participants with missing data for predictors and outcome. | Table S2 |
|  | 13c | V | For validation, show a comparison with the development data of the distribution of important variables (demographics, predictors and outcome). | NA |
| Model development | 14a | D | Specify the number of participants and outcome events in each analysis. | NA |
|  | 14b | D | If done, report the unadjusted association between each candidate predictor and outcome. | NA |
| Model specification | 15a | D | Present the full prediction model to allow predictions for individuals (i.e., all regression coefficients, and model intercept or baseline survival at a given time point). | NA |
|  | 15b | D | Explain how to the use the prediction model. | 3 |
| Model performance | 16 | D;V | Report performance measures (with CIs) for the prediction model. | Table 1 |
| Model-updating | 17 | V | If done, report the results from any model updating (i.e., model specification, model performance). | NA |
| **Discussion** | | | | |
| Limitations | 18 | D;V | Discuss any limitations of the study (such as nonrepresentative sample, few events per predictor, missing data). | 10 |
| Interpretation | 19a | V | For validation, discuss the results with reference to performance in the development data, and any other validation data. | 9-10 |
|  | 19b | D;V | Give an overall interpretation of the results, considering objectives, limitations, results from similar studies, and other relevant evidence. | 9 |
| Implications | 20 | D;V | Discuss the potential clinical use of the model and implications for future research. | 11 |
| **Other information** | | | | |
| Supplementary information | 21 | D;V | Provide information about the availability of supplementary resources, such as study protocol, Web calculator, and data sets. | 11 |
| Funding | 22 | D;V | Give the source of funding and the role of the funders for the present study. | 11 |

**REFERENCES**

Bertels, Jeroen, Tom Eelbode, Maxim Berman, Dirk Vandermeulen, Frederik Maes, Raf Bisschops, and Matthew Blaschko. 2019. “Optimizing the Dice Score and Jaccard Index for Medical Image Segmentation: Theory & Practice.” *Lecture Notes in Computer Science* 11765 LNCS (November): 92–100.

Bullmore, Ed, and Olaf Sporns. 2009. “Complex Brain Networks: Graph Theoretical Analysis of Structural and Functional Systems.” *Nature Reviews. Neuroscience* 10 (3): 186–98.

CJ Neurolab. 2018. “HCP-MMP1.0 Volumetric (NIfTI) Masks in Native Structural Space.” October 2018.

Esteban, Oscar, Christopher J. Markiewicz, Ross W. Blair, Craig A. Moodie, A. Ilkay Isik, Asier Erramuzpe, James D. Kent, et al. 2019. “FMRIPrep: A Robust Preprocessing Pipeline for Functional MRI.” *Nature Methods* 16 (1): 111–16.

Fischl, Bruce. 2012. “FreeSurfer.” *NeuroImage*. https://doi.org/10.1016/j.neuroimage.2012.01.021.

Glasser, Matthew F., Timothy S. Coalson, Emma C. Robinson, Carl D. Hacker, John Harwell, Essa Yacoub, Kamil Ugurbil, et al. 2016. “A Multi-Modal Parcellation of Human Cerebral Cortex.” *Nature* 536 (7615): 171–78.

Glasser, Matthew F., Stamatios N. Sotiropoulos, J. Anthony Wilson, Timothy S. Coalson, Bruce Fischl, Jesper L. Andersson, Junqian Xu, et al. 2013. “The Minimal Preprocessing Pipelines for the Human Connectome Project.” *NeuroImage* 80: 105–24.

Kathryn Mills. 2016. “HCP-MMP1.0 Projected on Fsaverage.” Https://figshare.com/articles/dataset/hcp-mmp1_0_projected_on_fsaverage/3498446. July 2016.

Schlemm, Eckhard, Robert Schulz, Marlene Bönstrup, Lutz Krawinkel, Jens Fiehler, Christian Gerloff, Götz Thomalla, and Bastian Cheng. 2020. “Structural Brain Networks and Functional Motor Outcome after Stroke—a Prospective Cohort Study.” *Brain Communications* 2 (1): 1–13.

Schulz, Robert, Anika Buchholz, Benedikt M. Frey, Marlene Bönstrup, Bastian Cheng, Götz Thomalla, Friedhelm C. Hummel, and Christian Gerloff. 2016. “Enhanced Effective Connectivity between Primary Motor Cortex and Intraparietal Sulcus in Well-Recovered Stroke Patients.” *Stroke; a Journal of Cerebral Circulation* 47 (2): 482–89.

Solodkin, A., U. Hasson, R. Siugzdaite, M. Schiel, E. E. Chen, R. Kotter, and S. L. Small. 2010. “Virtual Brain Transplantation (VBT): A Method for Accurate Image Registration and Parcellation in Large Cortical Stroke.” *Archives Italiennes de Biologie* 148 (3): 219–41.

Taha, Abdel Aziz, and Allan Hanbury. 2015. “Metrics for Evaluating 3D Medical Image Segmentation: Analysis, Selection, and Tool.” *BMC Medical Imaging* 15 (1): 1–28.
