# Supplementary material for: A lesion‐aware automated processing framework for clinical stroke magnetic resonance imaging
Source: Hum Brain Mapp. 2024 Jun 22;45(9):e26701. doi: 10.1002/hbm.26701 (PMC11193092; doi:10.1002/hbm.26701)
Supplement: Supplementary file 2 — Data S2: Supporting Information. [file HBM-45-e26701-s002.docx]

# **SUPPLEMENTARY MATERIAL - VISUALIZATIONS**

A lesion-aware automated processing framework for clinical stroke MRI

Patrik Bey^1,2^, Kiret Dhindsa^1,2^, Amrit Kashyap^1^, Michael Schirner^1,2,3,4,5^, Jan Feldheim^6^, Marlene Bönstrup^6(*)^, Robert Schulz^6^, Bastian Cheng^6^, Götz Thomalla^6^, Christian Gerloff^6^, Petra Ritter^1,2,3,4,5^

^1^Berlin Institute of Health at Charité – Universitätsmedizin Berlin, Berlin, Germany, ^2^ Department of Neurology with Experimental Neurology, Brain Simulation Section, Charité – Universitätsmedizin Berlin, corporate member of Freie Universität Berlin and Humboldt-Universität zu Berlin, Berlin, Germany, ^3^Bernstein Focus State Dependencies of Learning and Bernstein Center for Computational Neuroscience, Berlin, Germany, ^4^Einstein Center for Neuroscience Berlin, ^5^Einstein Center Digital Future, ^6^Klinik und Poliklinik für Neurologie, Kopf- und Neurozentrum, University Medical Center, Hamburg-Eppendorf, Germany

**Lesion based intensity abnormalities**

**
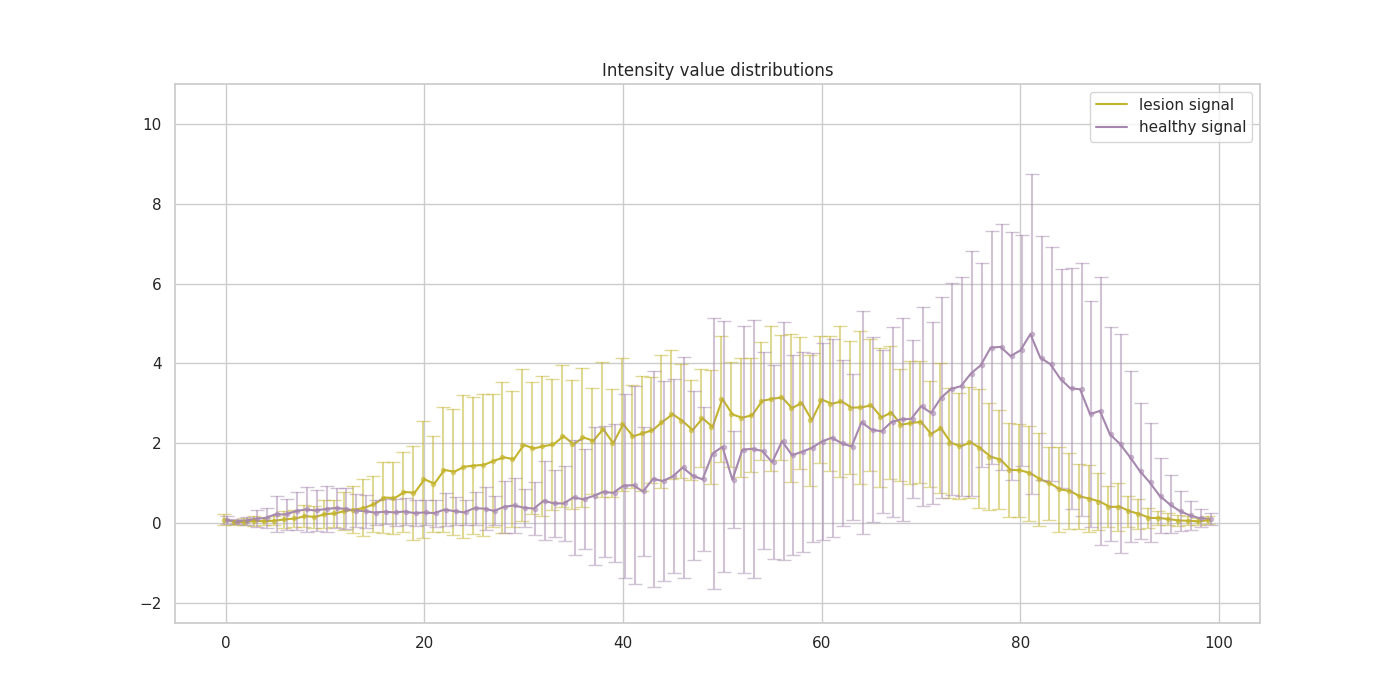
**The extent of abnormality of lesion voxel intensity was investigated by extracting intensity values from T1 weighted images at the lesion location, using the extracted lesion signal and healthy transplant signal created during virtual brain transplant (**Figure S2 (F)**) step of the *structural pipeline* of LeAPP. The subject based comparison showed a significant difference between healthy and lesion intensity distributions (Fisher’s combined test: statistic = 17662.35, p-value = 0.0)

**Figure SV1 Intensity distributions** of T1 images of healthy and lesioned tissue at lesion location and contra hemispheric location from acute stroke patients. We see a significantly flatter curve for lesion signal values, indicating a broader spectrum of intensity values compared to healthy brain signal as evident by increased skewness (lesion signal = 0.56, healthy signal = -0.75) and kurtosis (lesion signal = 1.96, healthy signal = 0.1). We hypothesize this abnormality to lead to less defined intensity gradients, potentially causing a reduction of accuracy for e.g., tissue type segmentation algorithms and surface reconstruction.

**Functional Magnetic Resonance Imaging data quality**


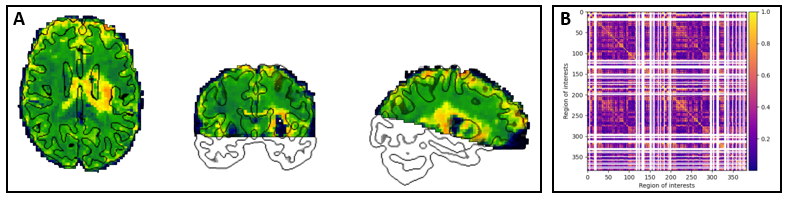
The present fMRI data used during development of this study highlights several challenges for processing fMRI data in the clinical context of stroke. The most prominent aspect is the reduced Field-of-view (FOV) during acquisition which significantly limits the extent of the recorded brain signal only capturing the cerebrum from apex to Sylvian fissure (**Figure SV2 (A)**). This aspect limits, for example, the ability to perform surface-based fMRI analysis as performed during HCPs fMRISurface pipeline (Glasser et al. (2013)). We therefore did not include surface-based processing of functional MRI data in the present study. Furthermore, this led to empty time series during extraction of average time series per ROI to compute the functional connectome (**Figure SV2 (B)**) as data was not acquired for several brain regions outside of the FOV.

**Figure SV2 FMRI data challenges** The data used in the current study shows a significantly reduced FOV for fMRI sequences. Therefore, fMRI time series of some brain regions are missing. The outline of an exemplary subject specific parcellation image overlayed to the corresponding T1w registered fMRI reference image (**A**) shows the missing regions including temporal cortex areas, cerebellum, and large parts of the occipital cortex. This leads to empty rows and columns in the resulting functional connectome (**B**).

**Lesion masks**

All individual lesion masks are shown below. The visualization is the individual lesion mask in subject specific T1w space, as created during the structural processing pipeline of LeAPP, overlayed to a MNI based template glass brain. This might cause minor inaccuracies in the overlap but provides a good approximation of the lesion extent at each timepoint for each patient.


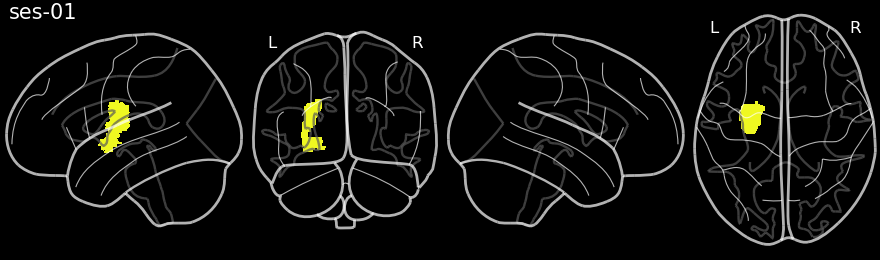
sub-P007


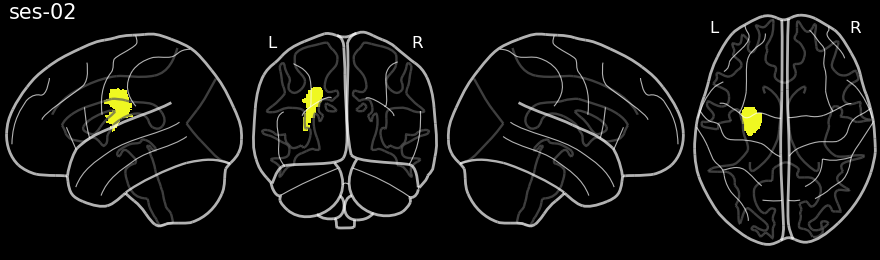

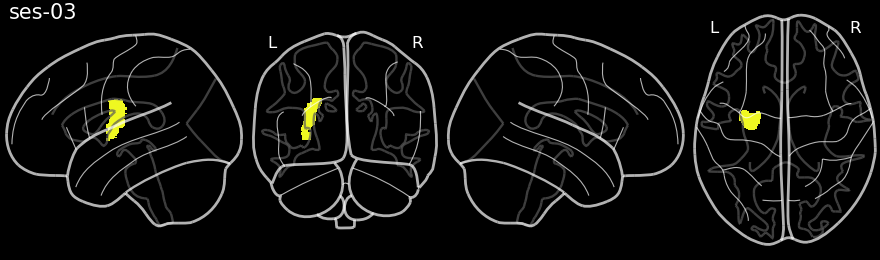

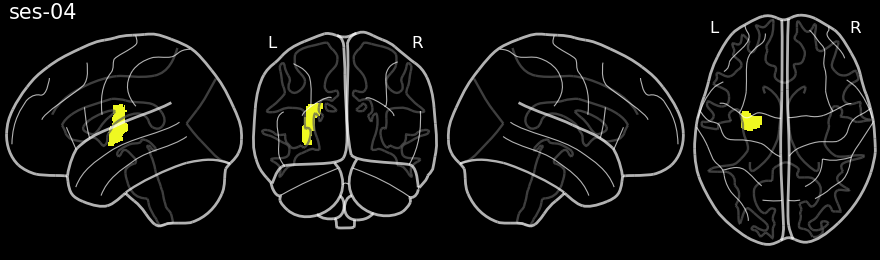


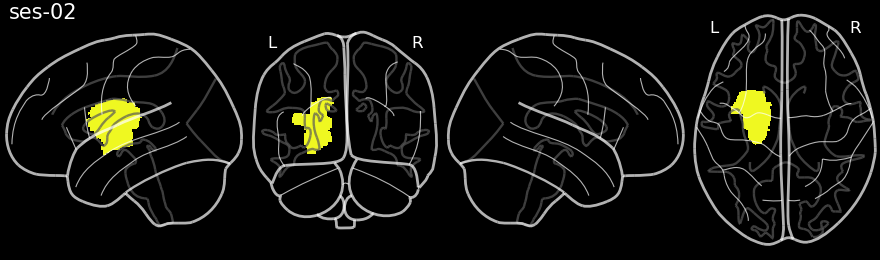

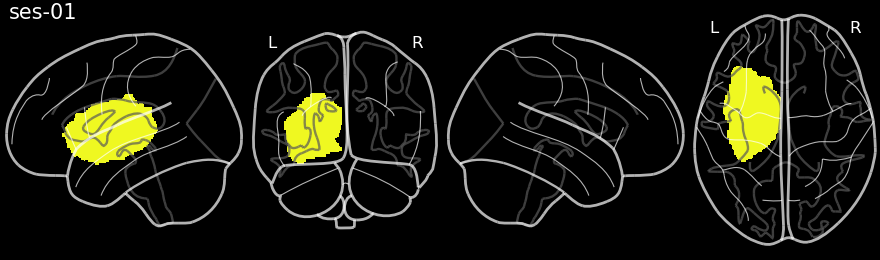
sub-P009


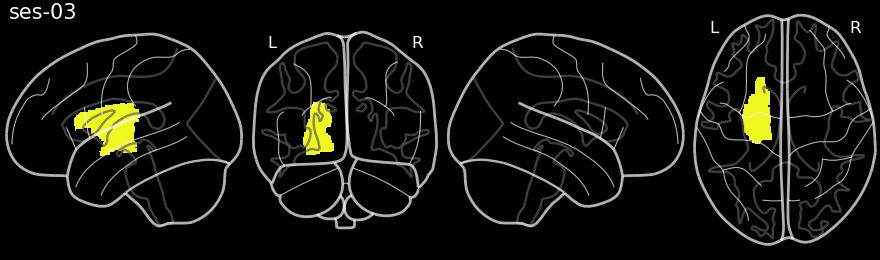

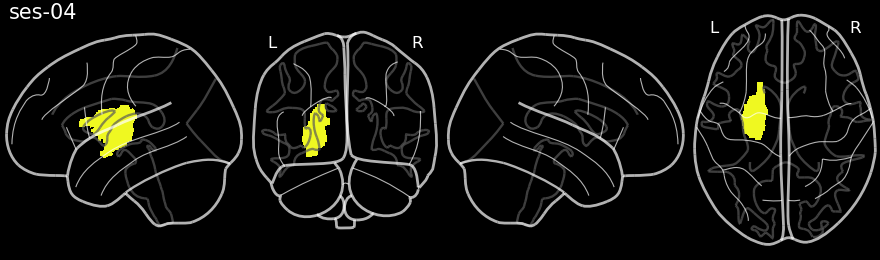


sub-P012


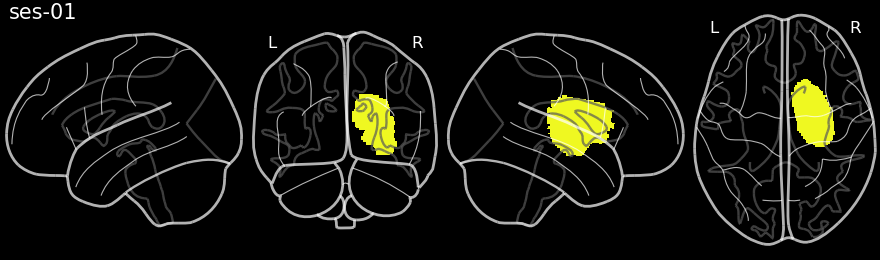

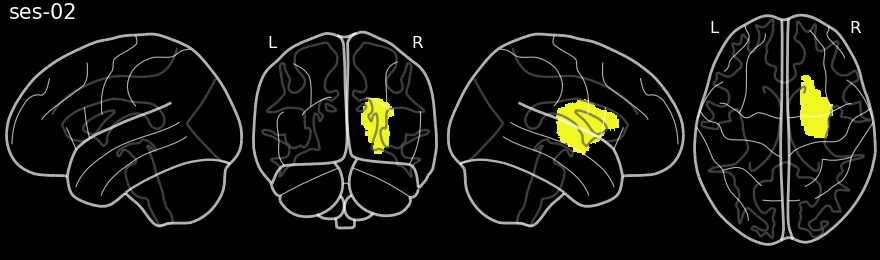

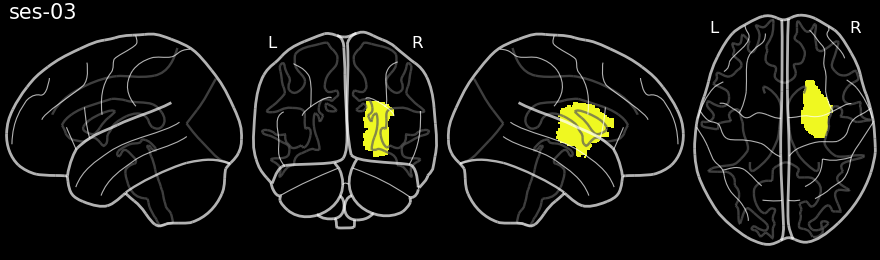

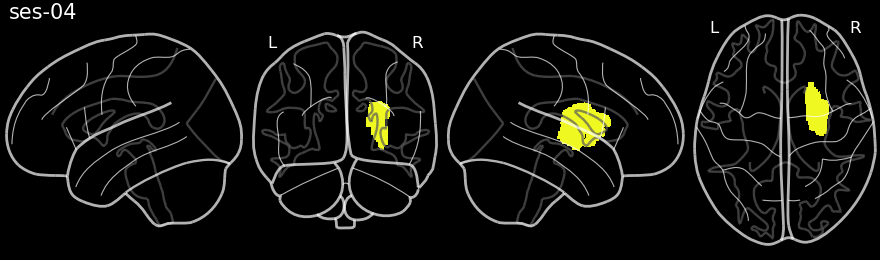


sub-P015


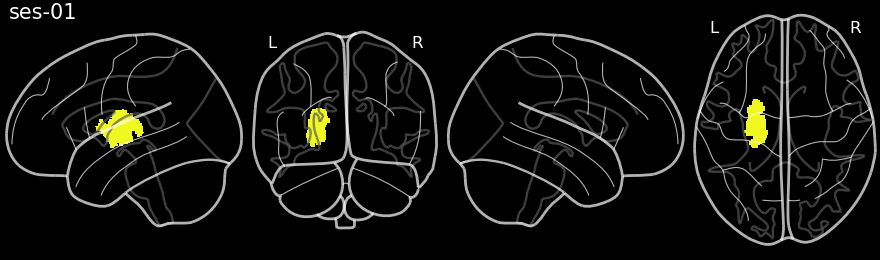

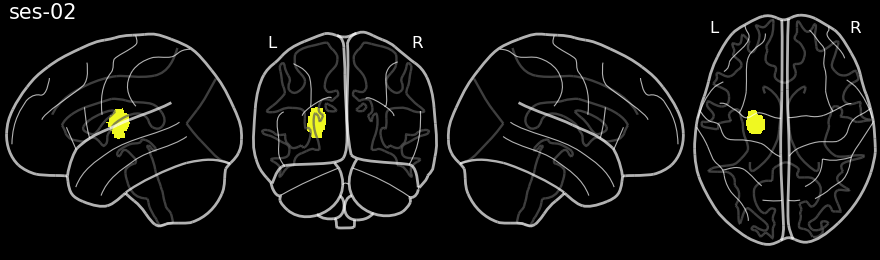

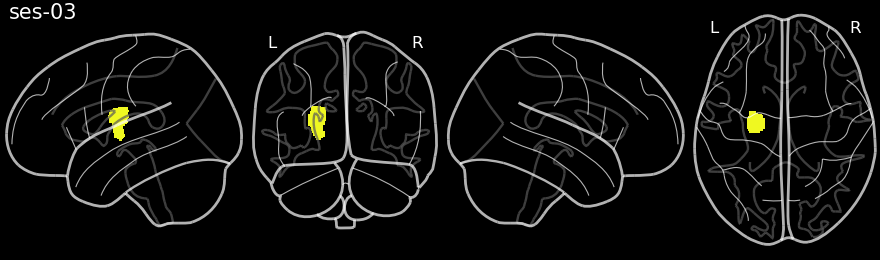

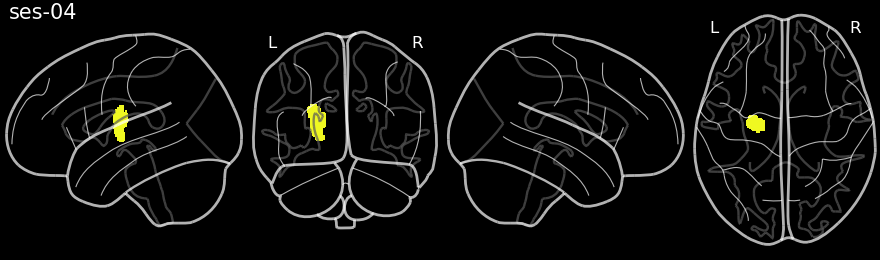


sub-P016


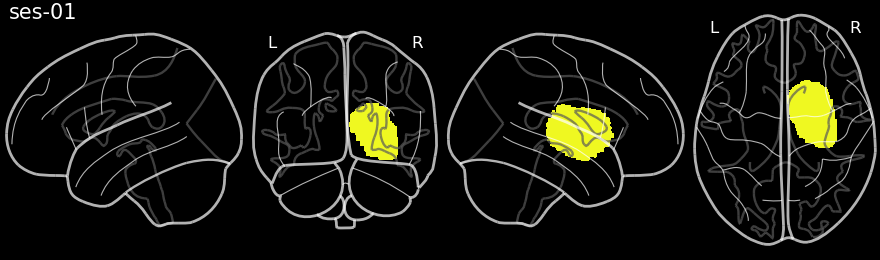

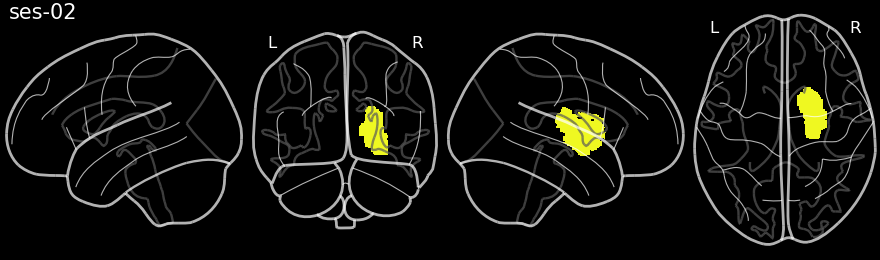

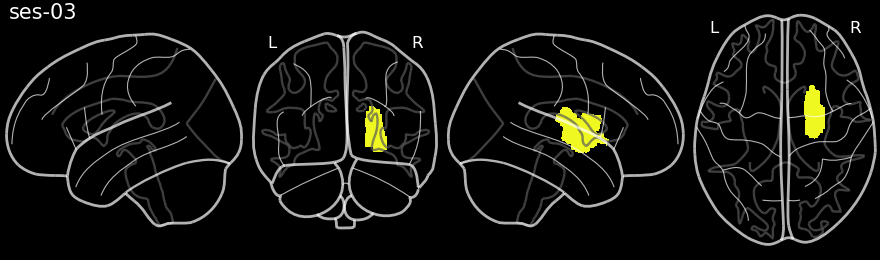

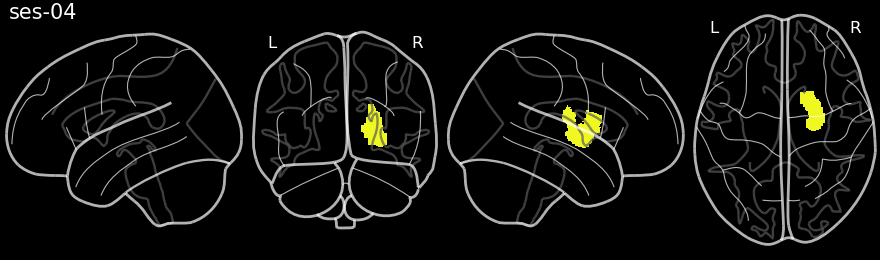


sub-P019


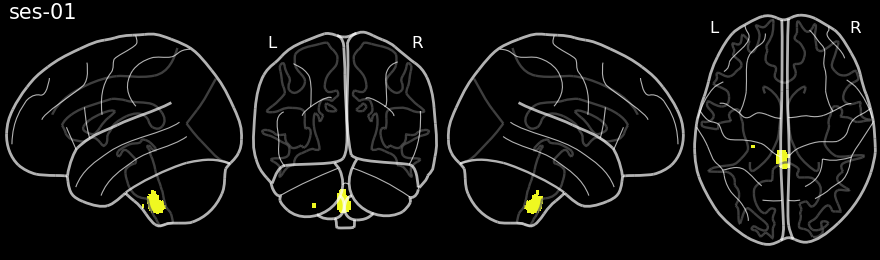


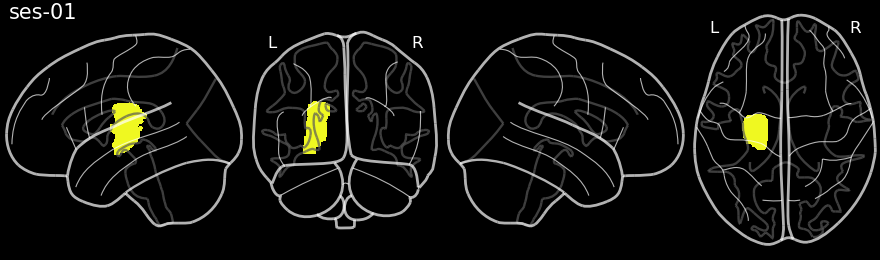
sub-P020


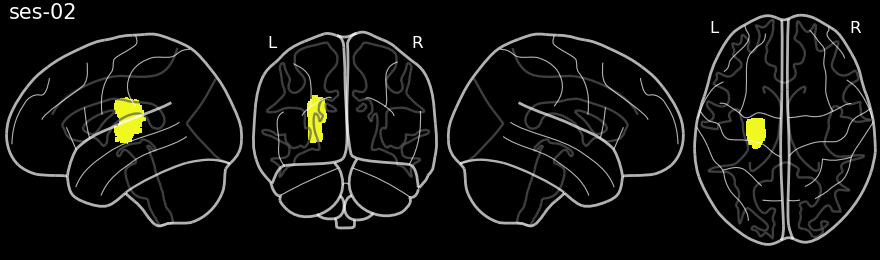

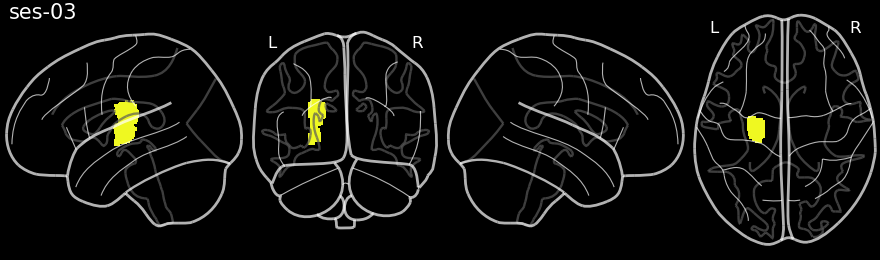

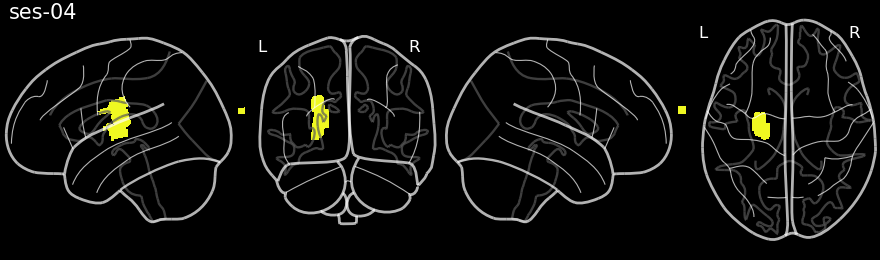


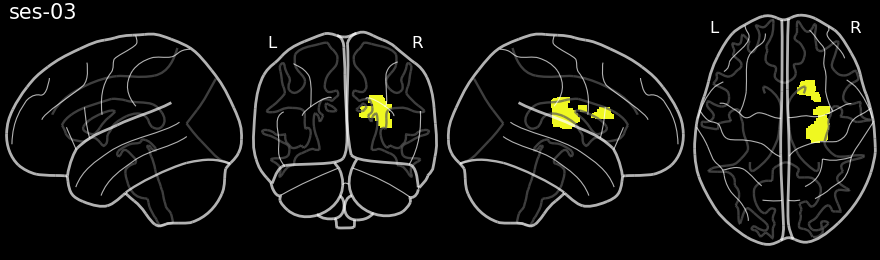

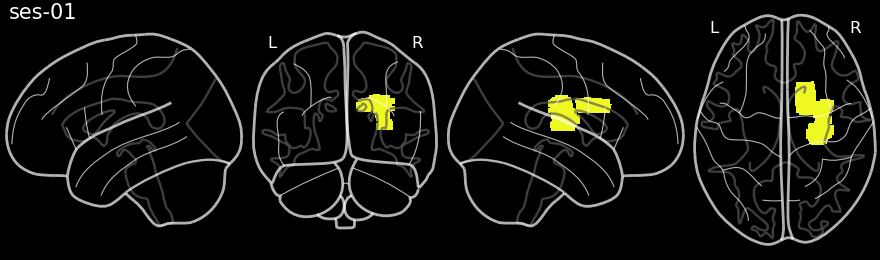
sub-P022

sub-P023


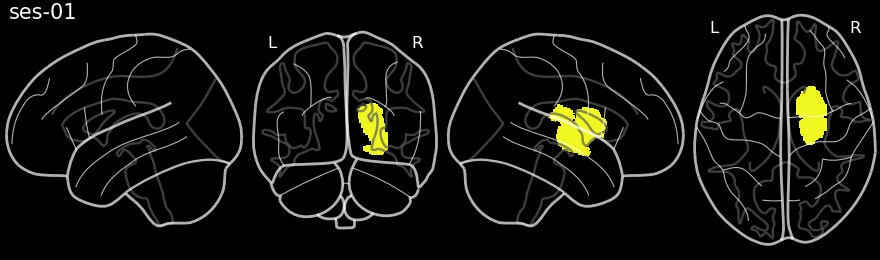

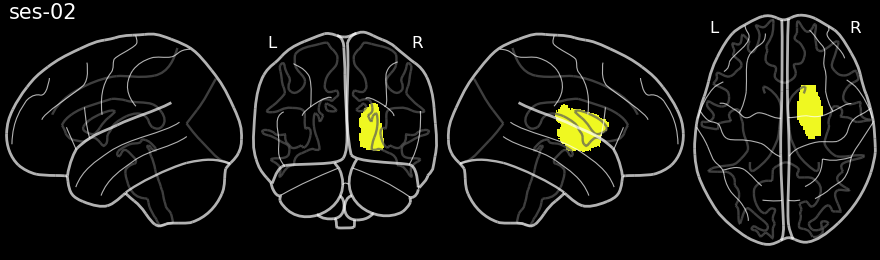


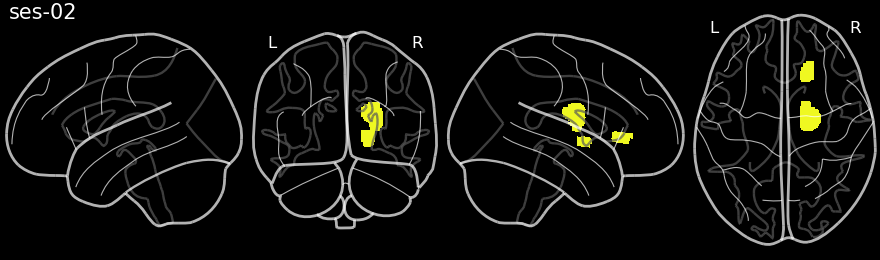

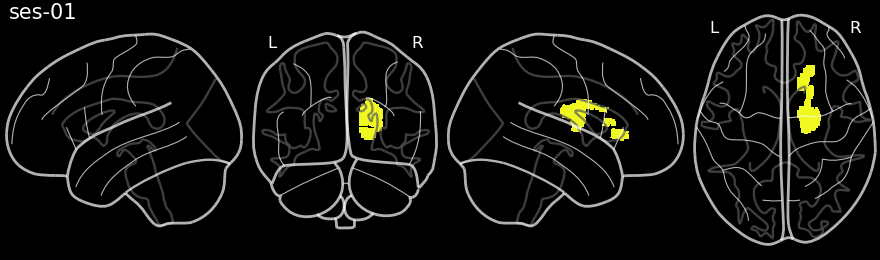
sub-P024


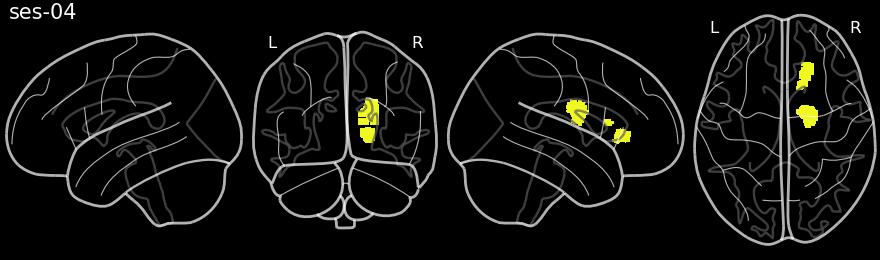


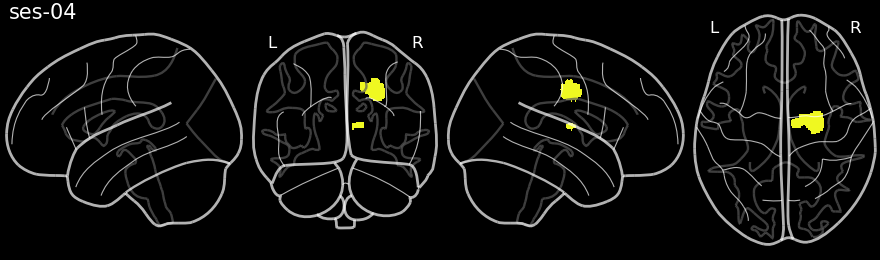

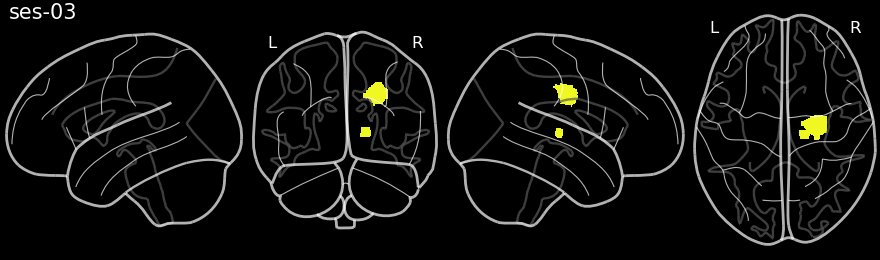
sub-P025
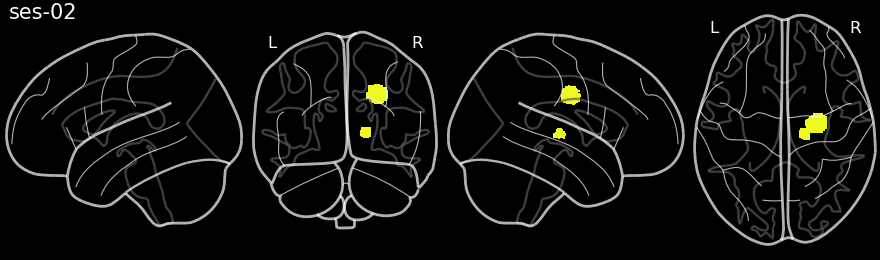

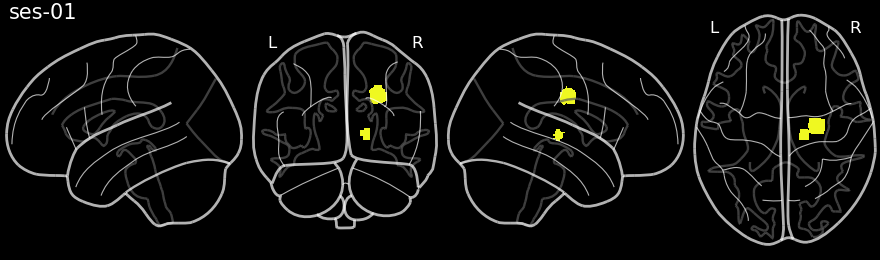


sub-P026


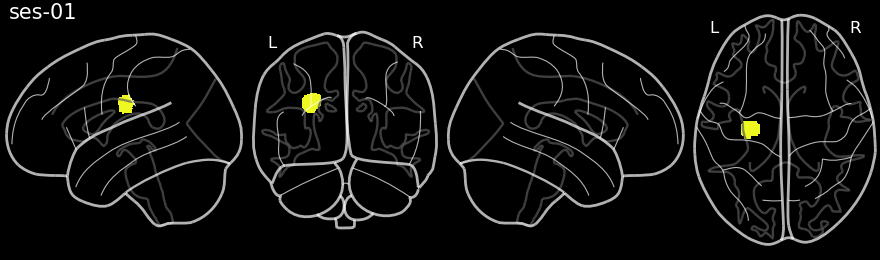

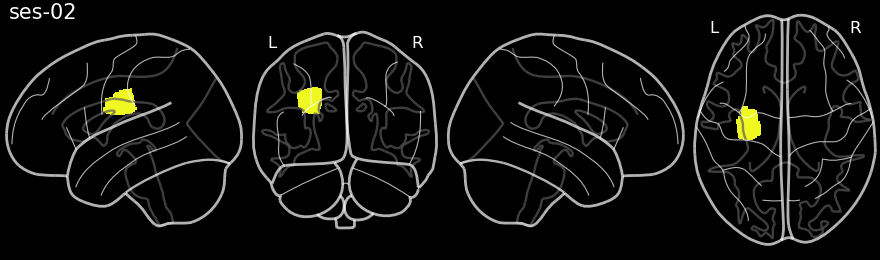

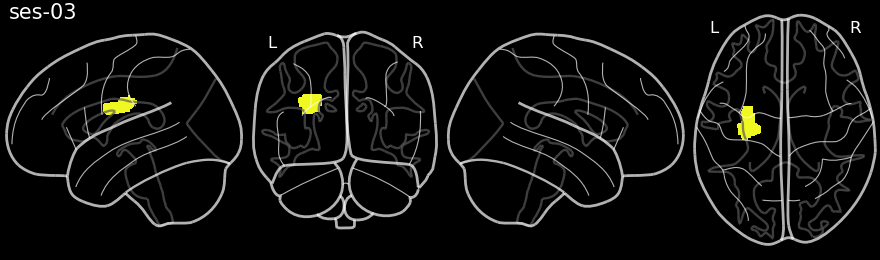

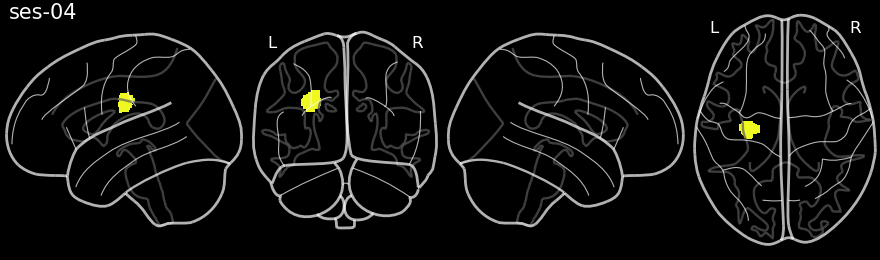


sub-P027


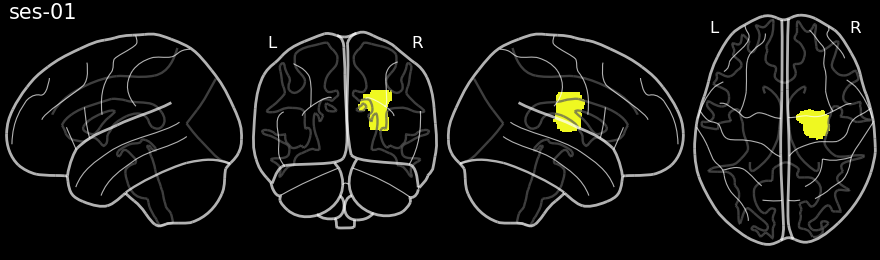


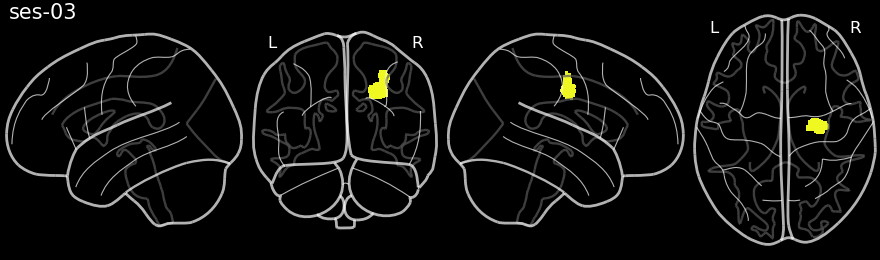
sub-P030
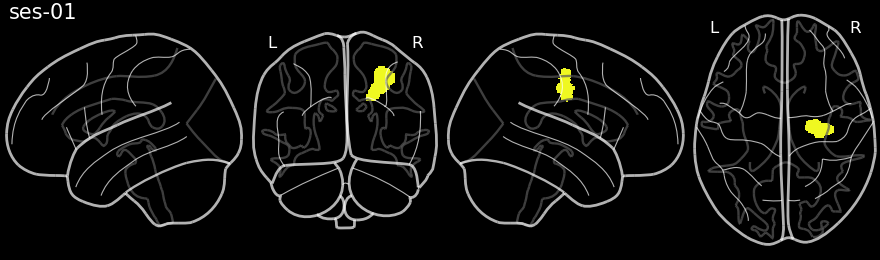

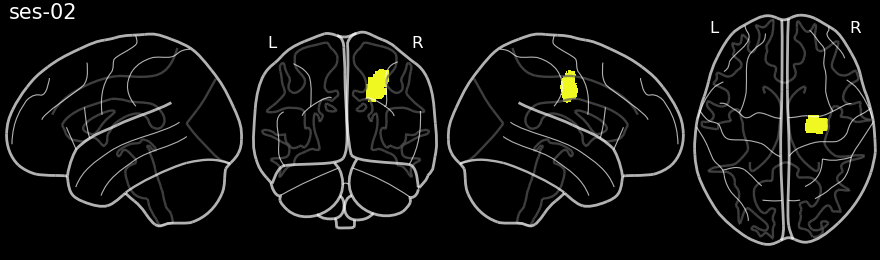


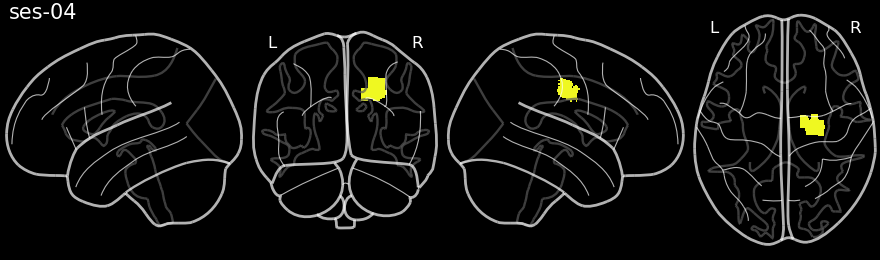


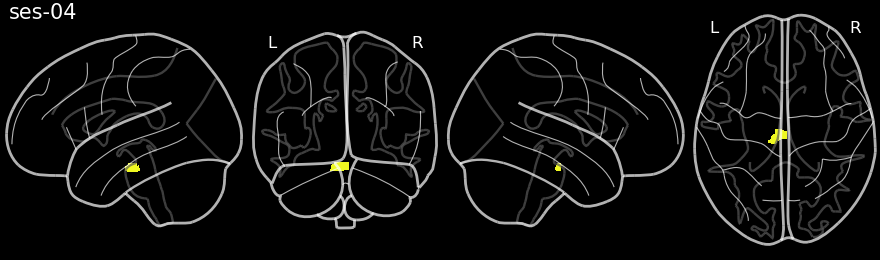

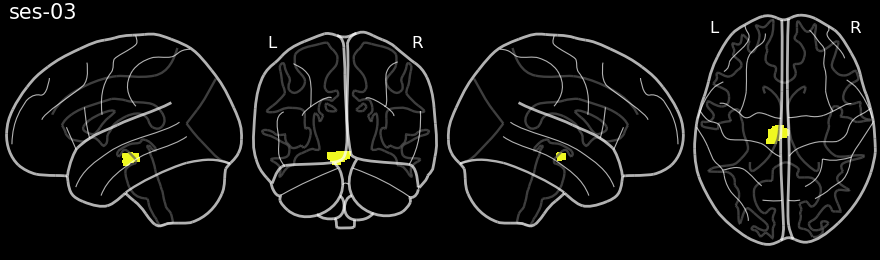
sub-P031
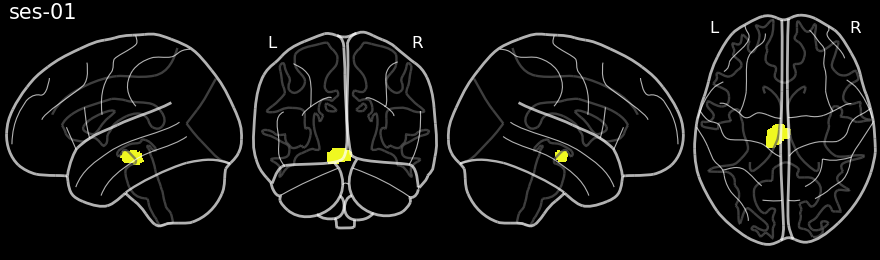

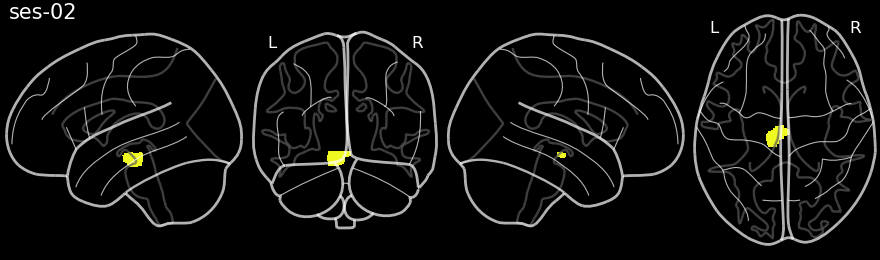


sub-P032


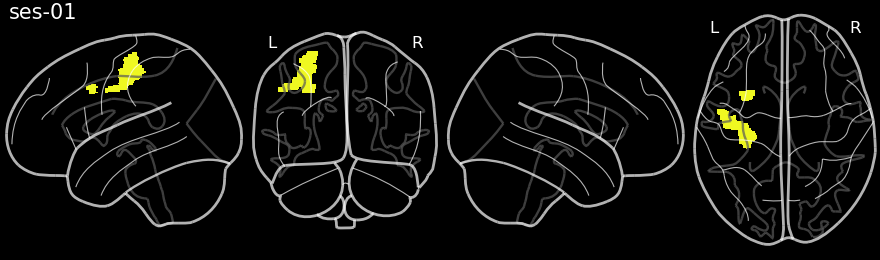

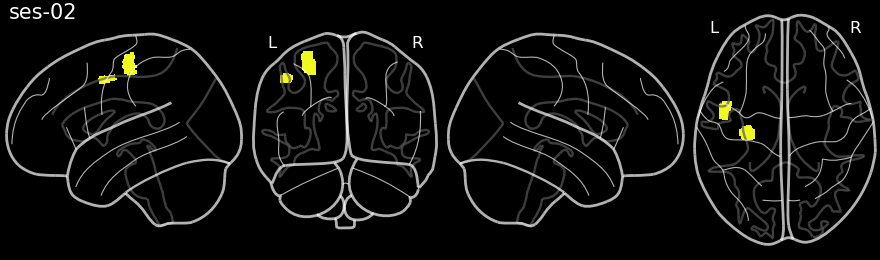

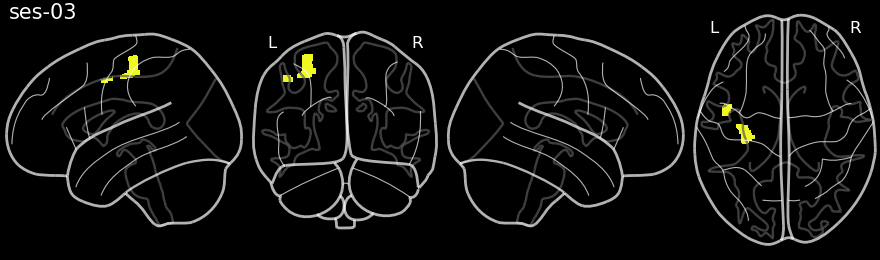


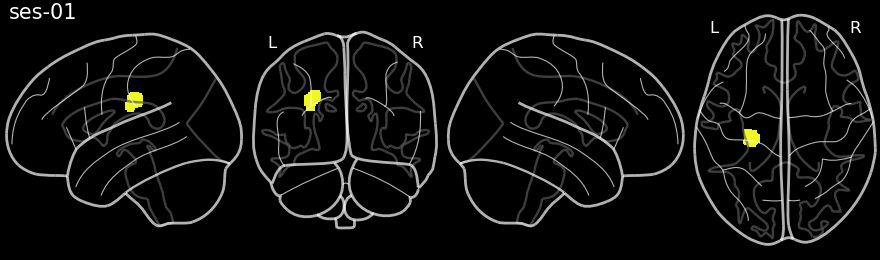
sub-P033


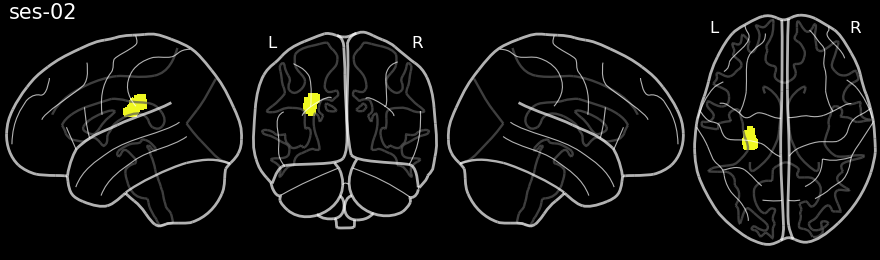

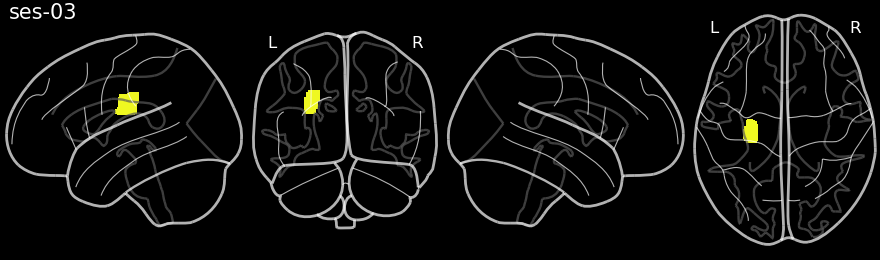

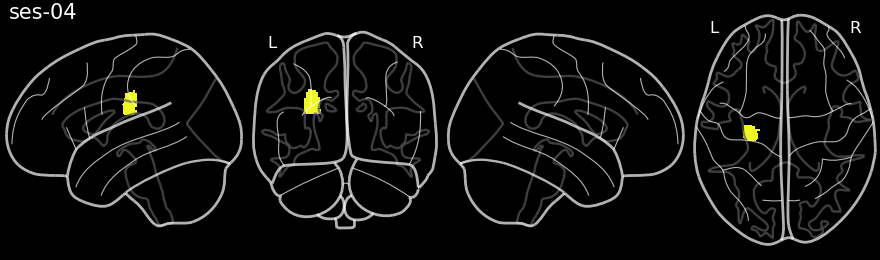


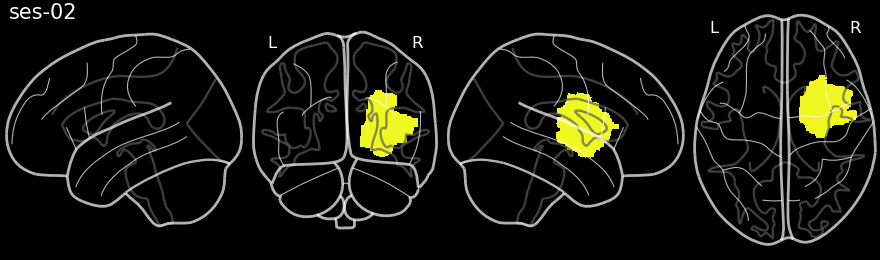

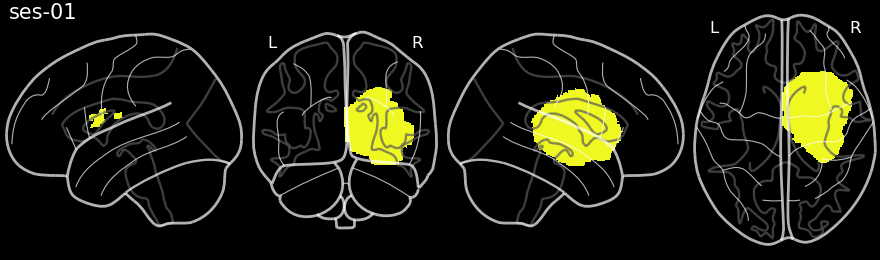
sub-P034


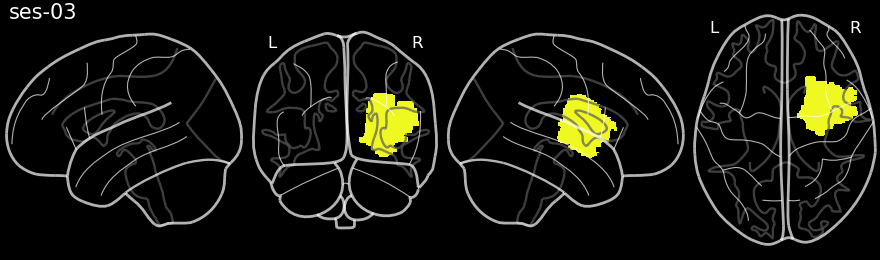

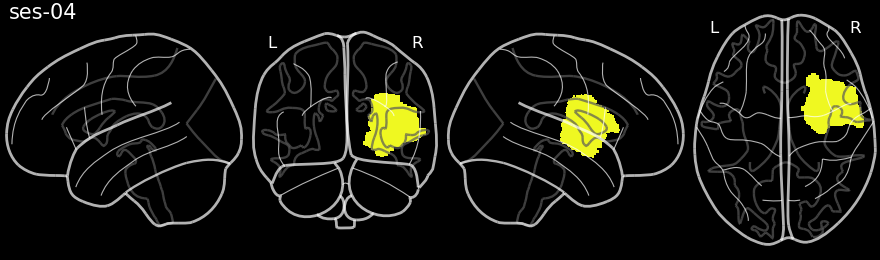


sub-P035


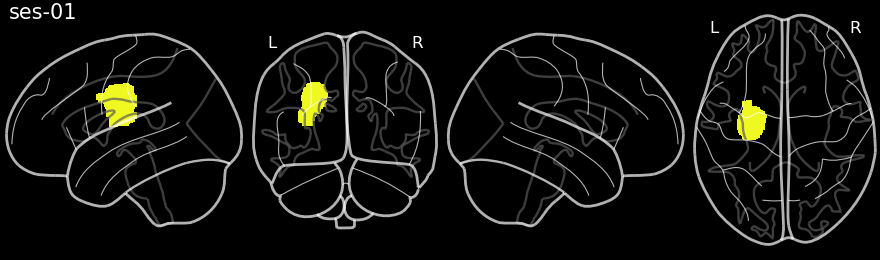

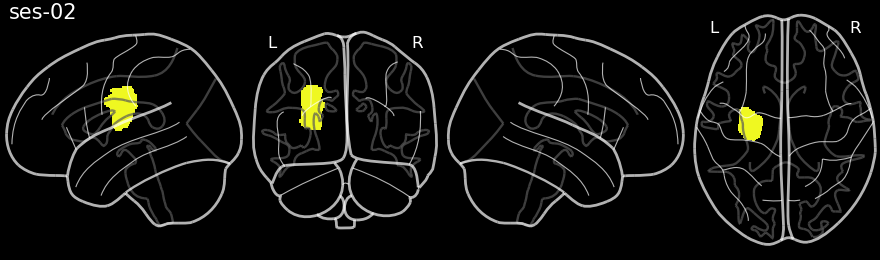

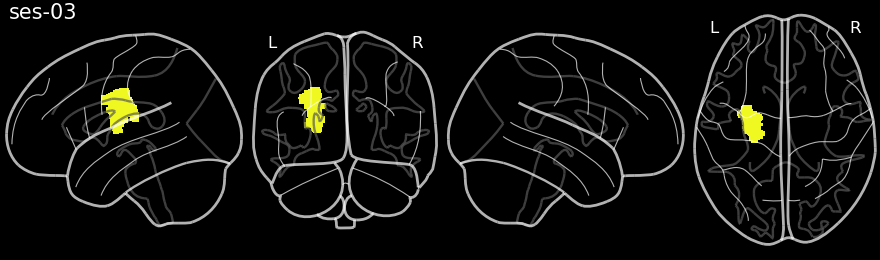

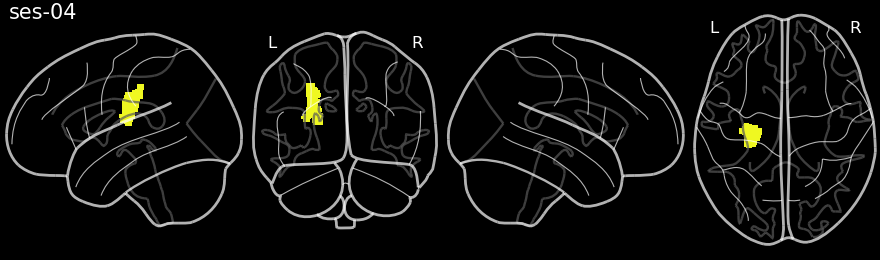


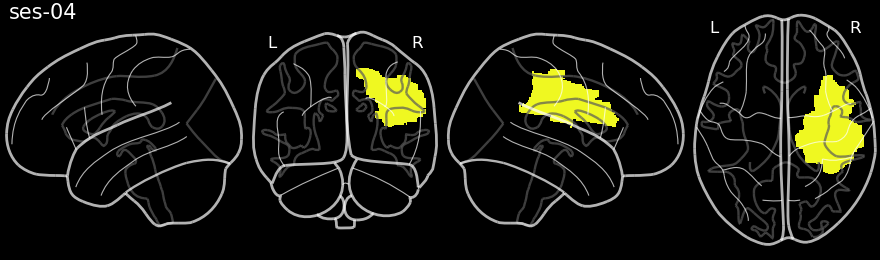

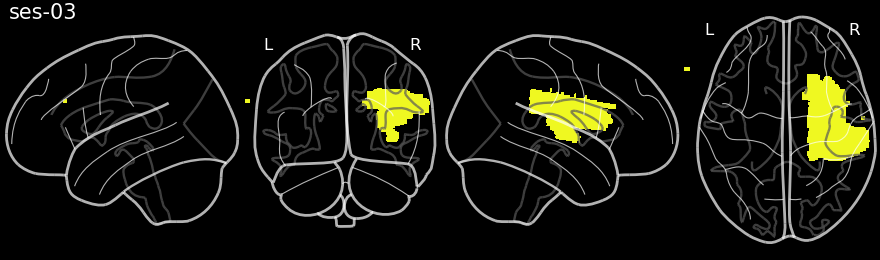

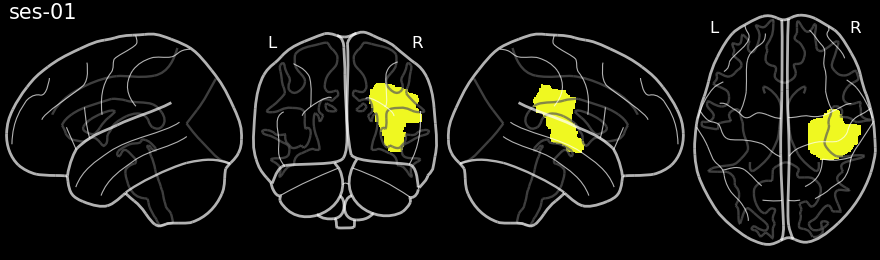
sub-P036

sub-P037


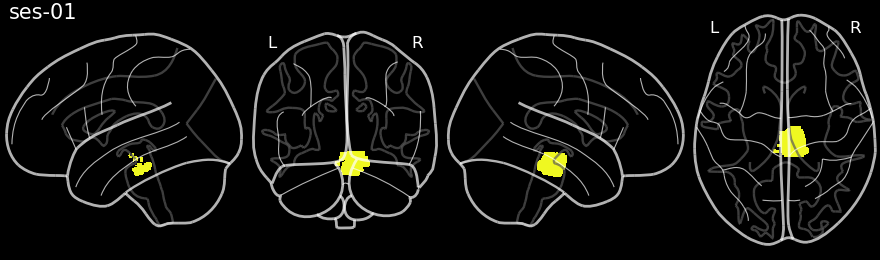


sub-P038


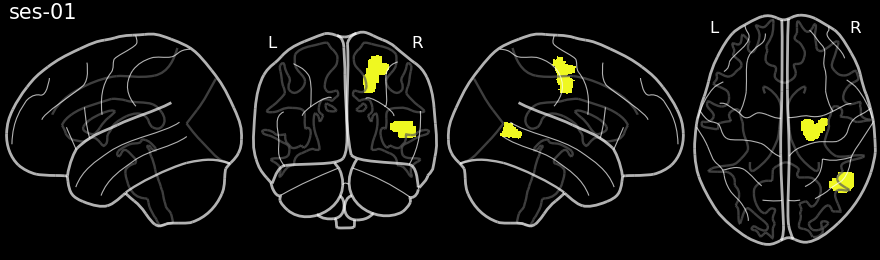


sub-P039


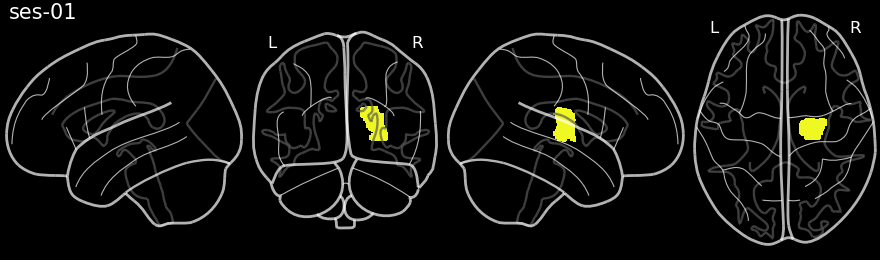


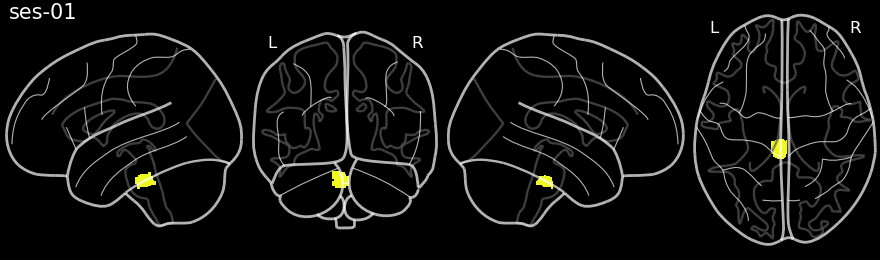
sub-P041


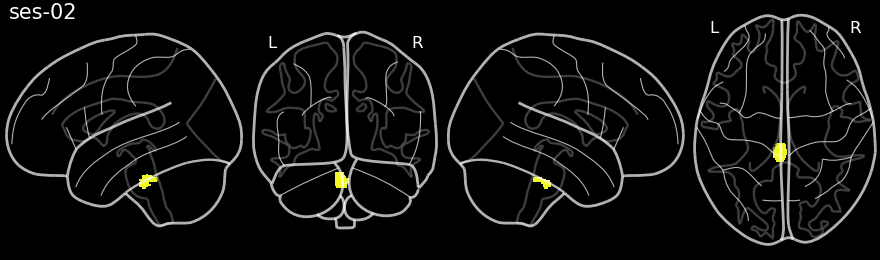

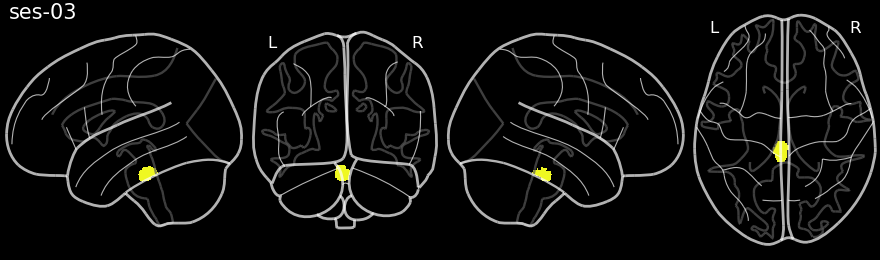

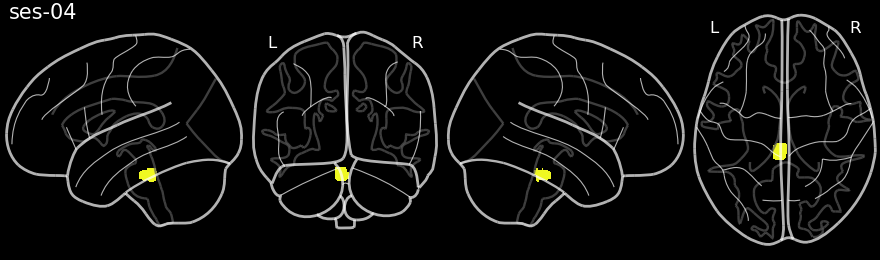


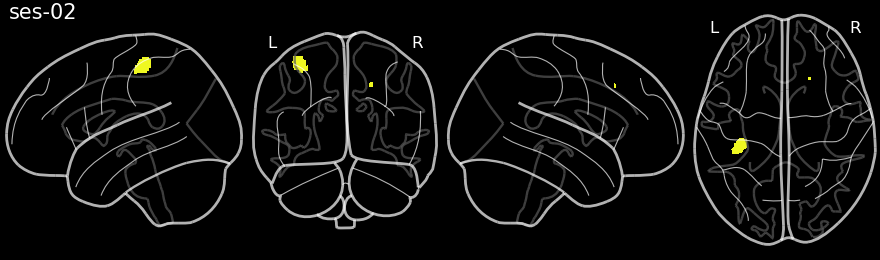

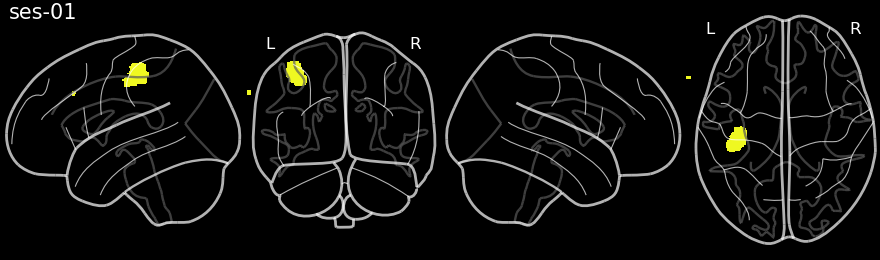
sub-P042

sub-P043


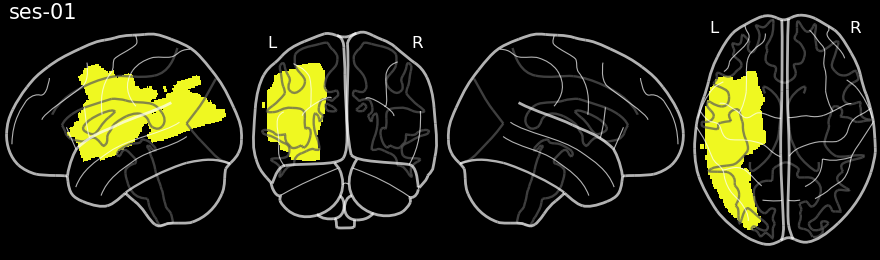

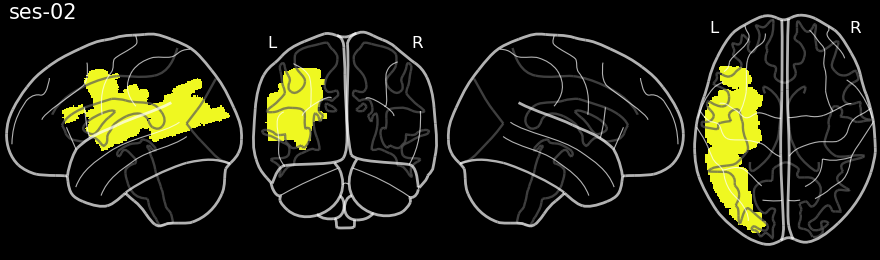

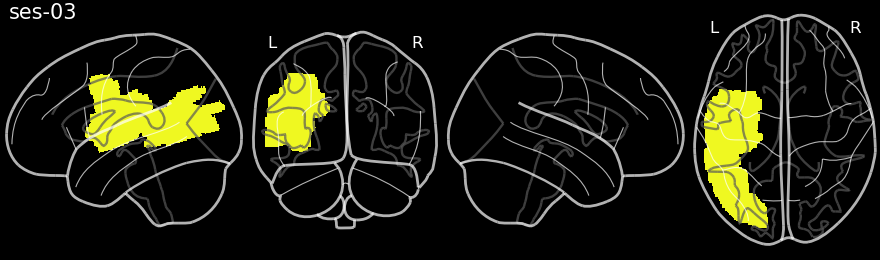

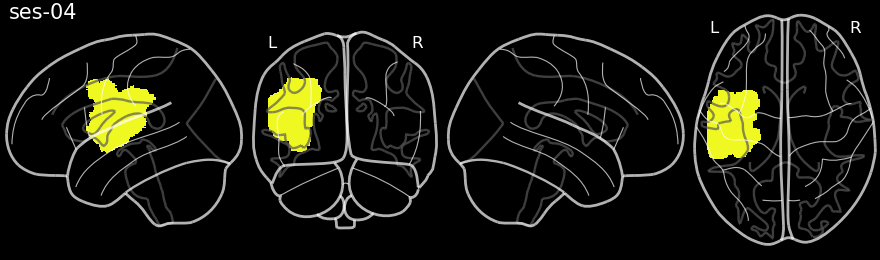


sub-P044


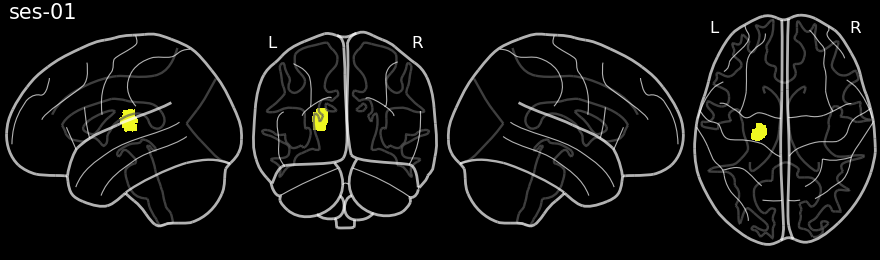


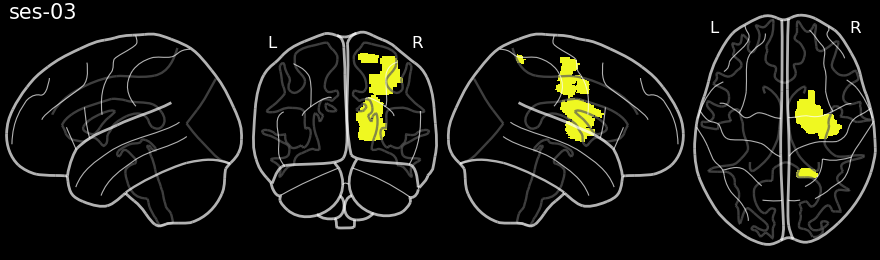
sub-P045
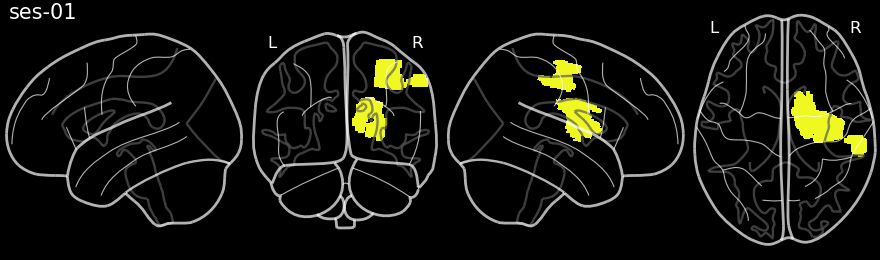

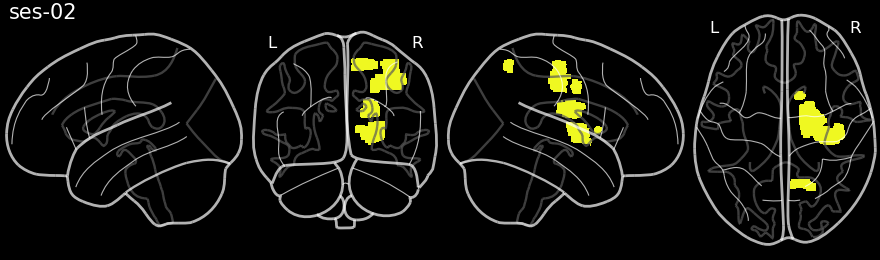


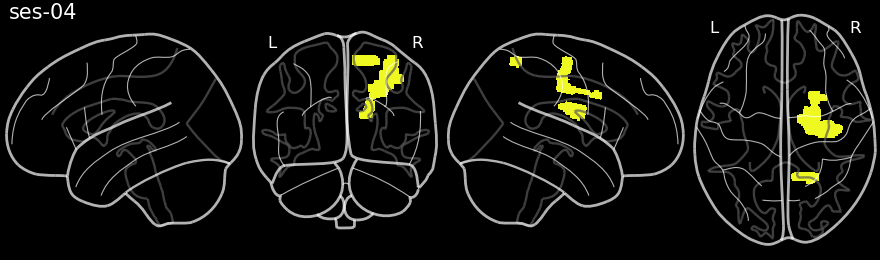


sub-P046


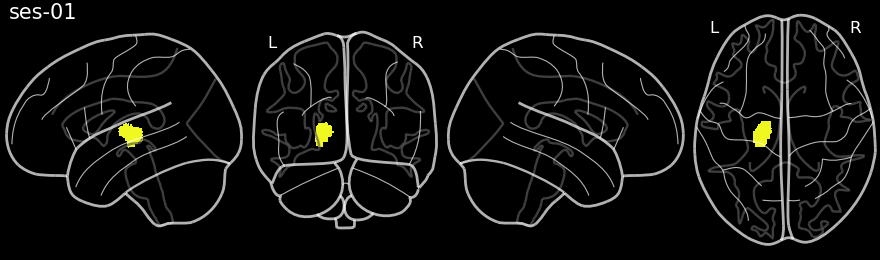

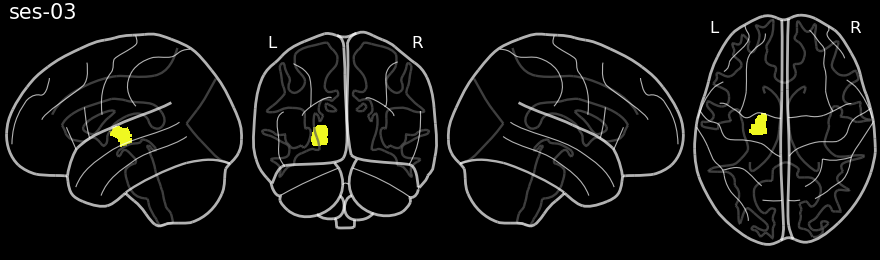

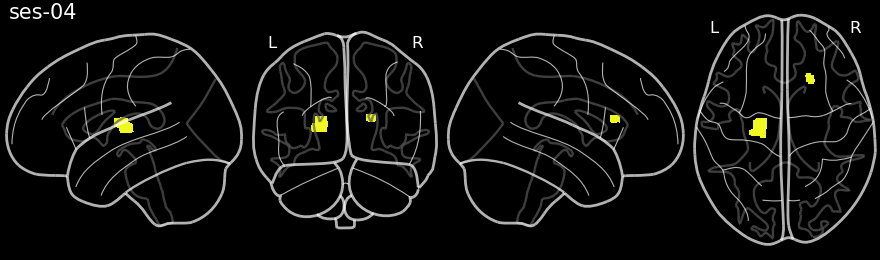


sub-P048


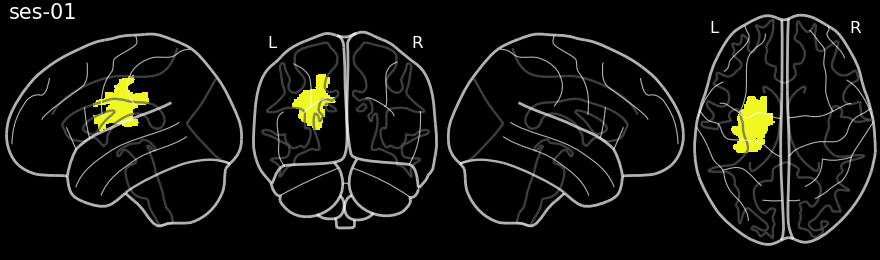


sub-P053


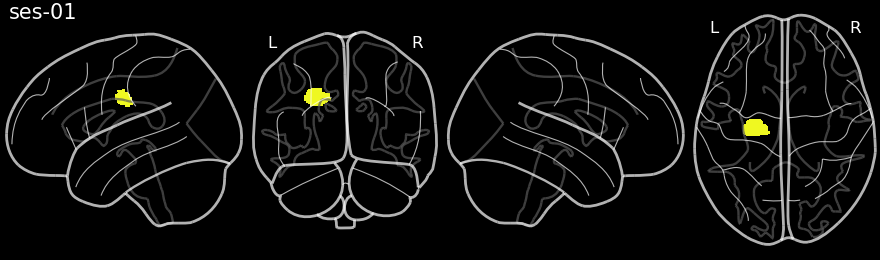

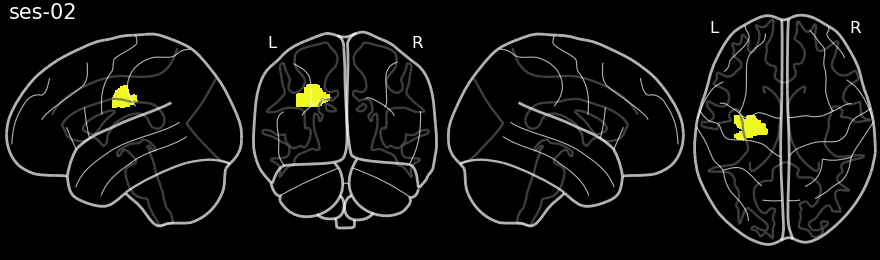


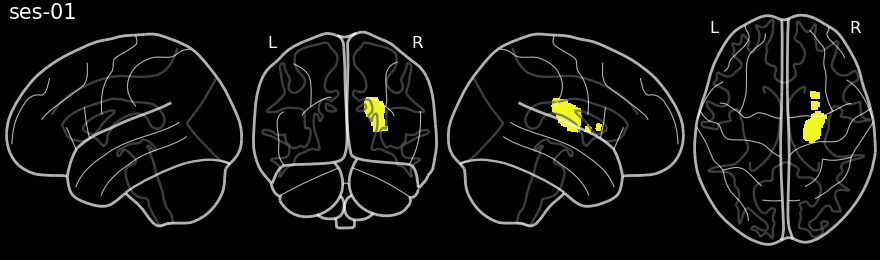
sub-P055


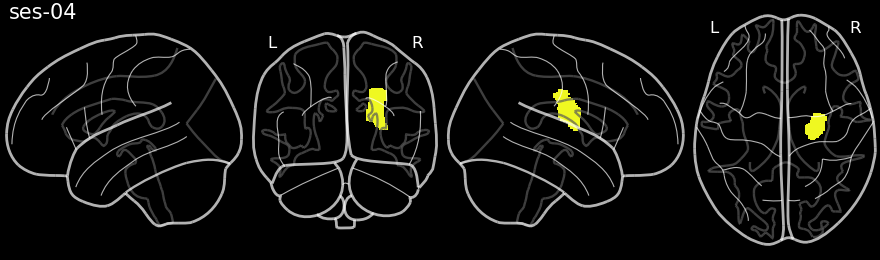


sub-P056


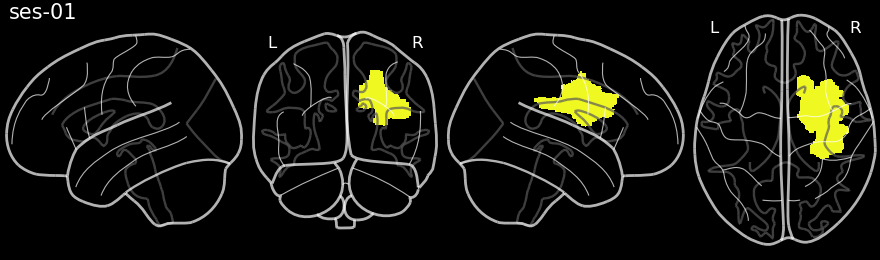

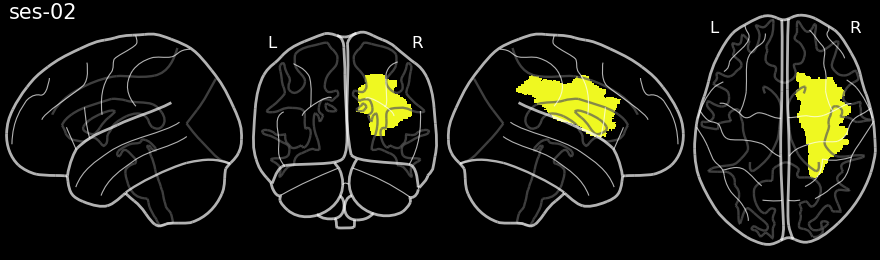


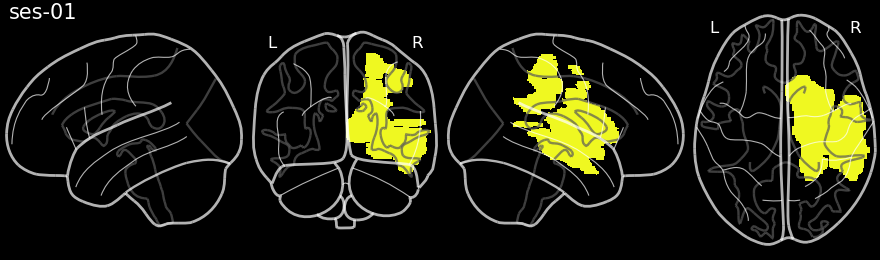
sub-P057


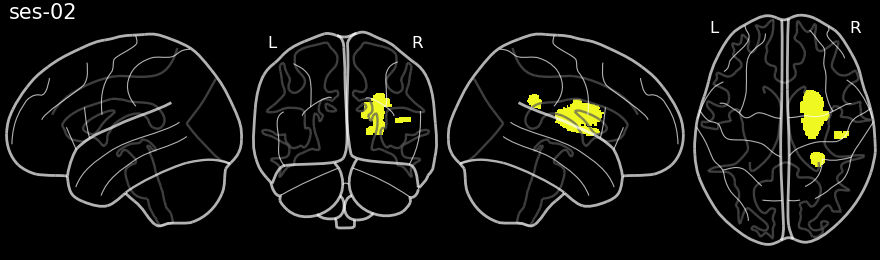


sub-P058


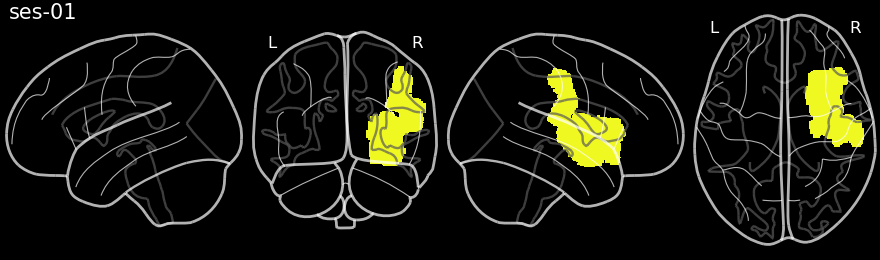


sub-P059

**Processing results**

All included subjects were processed and the results are displayed below. The overview includes the initial T1w image volume centered at the center-of-mass of the lesion (purple cross) and lesion outline overlay (**A**), the fully processed T1w image with the corresponding individual brain parcellation overlay (**B**), the first image of the fully processed fMRI volume with binarized brain parcellation overlay (**C**), the extracted functional connectome (FC) for the full time series for an exemplary task (**D**) as well as the structural connectome weights rescaled to 95% quantile for visualization (**E**).

*Healthy controls*

sub-H001

sub-H002

sub-H003

sub-H004

sub-H006

sub-H008

sub-H009

sub-H010

sub-H011

sub-H012

sub-H014

sub-H015

sub-H018

sub-H020

sub-H023

*Patients*

sub-P007

sub-P009

sub-P012

sub-P015

sub-P016

sub-P019

sub-P020

sub-P022

sub-P023

sub-P024

sub-P025

sub-P026

sub-P027

sub-P030

sub-P031

sub-P032

sub-P033

sub-P034

sub-P035

sub-P036

sub-P037

sub-P038

sub-P039

sub-P041

sub-P042

sub-P043

sub-P044

sub-P045

sub-P046

sub-P048

sub-P053

sub-P055

sub-P056

sub-P057

sub-P058

sub-P059
